# Iron-mediated ligand-to-metal charge transfer enables 1,2-diazidation of alkenes

**DOI:** 10.1038/s41467-022-35344-9

**Published:** 2022-12-23

**Authors:** Muliang Zhang, Jinghui Zhang, Qingyao Li, Yumeng Shi

**Affiliations:** 1 International Collaborative Laboratory of 2D Materials for Optoelectronics Science and Technology of Ministry of Education, Institute of Microscale Optoelectronics, Shenzhen University, 518060 Shenzhen, People’s Republic of China; 2 Institut für Chemie, Technische Universität Berlin, Strasse des 17. Juni 115, 10623 Berlin, Germany; 3 Department of Chemistry, National University of Singapore, 3 Science Drive 3, 117543 Singapore, Republic of Singapore

**Keywords:** Photocatalysis, Synthetic chemistry methodology

## Abstract

Given the widespread significance of vicinal diamine units in organic synthesis, pharmaceuticals and functional materials, as well as in privileged molecular catalysts, an efficient and practical strategy that avoids the use of stoichiometric strong oxidants is highly desirable. We herein report the application of ligand-to-metal charge transfer (LMCT) excitation to 1,2-diazidation reactions from alkenes and TMSN_3_ via a coordination-LMCT-homolysis process with more abundant and greener iron salt as the catalyst. Such a LMCT-homolysis mode allows the generation of electrophilic azidyl radical intermediate from Fe–N_3_ complexes poised for subsequent radical addition into carbon–carbon double bond. The generated carbon radical intermediate is further captured by iron-mediated azidyl radical transfer, enabling dual carbon–nitrogen bond formation. This protocol provides a versatile platform to access structurally diverse diazides with high functional group compatibility from readily available alkenes without the need of chemical oxidants.

## Abstract (web summary)

Alkene diazidations represent a promising strategy for the synthesis of 1,2-diamines. Here, the authors report a protocol that enables alkene diazidation via iron-mediated ligand-to-metal charge transfer, providing a versatile platform to access structurally diverse diazides without using external oxidants.

## Introduction

**Fig. 1:**
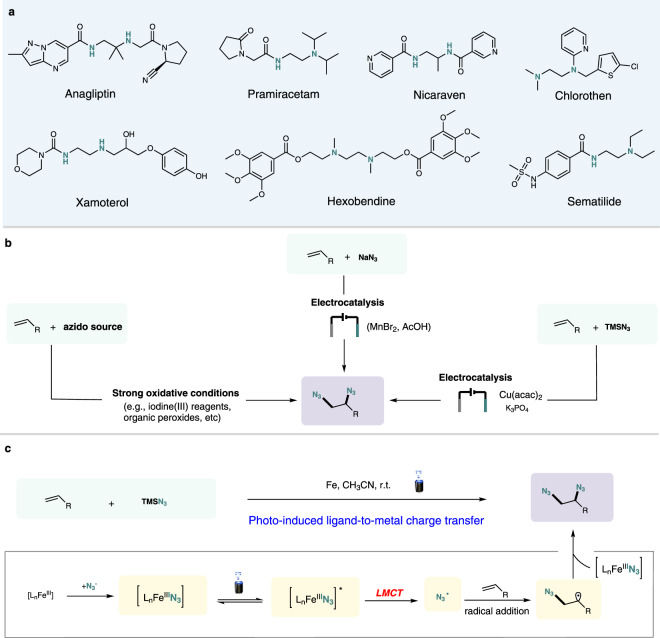
Representative pharmaceutical compounds and common strategies. **a** Representative pharmaceutical compounds containing vicinal diamine moieties. **b** Prior state of the art of alkenes diazidation reactions. **c** Photo-induced ligand-to-metal charge transfer enables alkene diazidation. ligand-to-metal charge transfer (LMCT).

Vicinal diamine units are ubiquitous in nature, and a variety of prevalent natural and synthetic medicines contain diamine functional group structures, which can be easily found in top-selling drugs such as Anagliptin, Pramiracetam, Nicaraven, Hexobendine and so on (Fig. 1a)^1,2^. The vital importance of diamine structures in chemistry, pharmaceuticals and biology has driven the development of different synthetic pathways to access them from readily available starting materials^3–5^. Although huge efforts have been made to develop straightforward and efficient alkene diamination strategies, it remains a big challenge to directly incorporate two amino groups into carbon–carbon double bonds, giving rise to 1,2-diamines, particularly free primary 1,2-diamines.

1,2-Diazidation reaction of alkenes represents a very promising alternative strategy for synthesis of 1,2-diamine compounds for the reason that the resulting vicinal diazide can be readily reduced to free primary 1,2-diamines^6,7^. Moreover, organic azides have found notable applications in 1,3-dipolar cycloaddition^8^, inert C–H bond amination^9,10^, the aza-Wittig reaction^11^ and Staudinger ligation^12^, owing to the unique reactivity. The conventional methods of alkene diazidation require stoichiometric quantities of strong oxidizing reagents^13–21^ such as hypervalent iodines and organic peroxides (Fig. 1b, left). The use of strong and indiscriminate oxidizing agents is incompatible with many sensitive functional groups, limiting their further application in the modern organic synthesis and pharmaceutically relevant studies. As a mild alternative, electrochemistry has provided an attractive strategy for chemical transformations in recent years owing to the avoidance of stoichiometric oxidant or reductant^22–24^. In this context, Lin and co-workers reported an electrocatalytic 1,2-diazidiation of alkenes, using MnBr_2_ as the catalyst under acidic conditions in 2017 (Fig. 1b, middle)^25^. Later, Lin and colleagues discovered that diazidiation of alkenes can be promoted under metal-free conditions by using an aminoxyl catalyst instead of metal catalyst and acidic conditions^26^. Very recently, Cu-electrocatalytic alkene diazidiation has been developed by Xu group, and the copper catalyst loading could be reduced to the ppm level in this system (Fig. 1b, right)^27^.

Developing operationally simple and mechanistically distinct catalytic reactivity modes for alkene diazidation remain highly desirable, which would offer more efficient and environmentally sustainable alternatives to established strategies. From the green chemistry points of perspective^28–30^, there is a longstanding interest in replacing those harmful metals by more and greener earth-abundant elements. Iron is one of the most abundant and safest metal on Earth, and application of Fe complexes in organic synthesis has attracted considerable attention from chemists^31–35^. Recent research has unlocked ligand-to-metal charge transfer (LMCT) process of Fe complexes through visible-light irradiation^36–38^. However, this LMCT excitation mode^39^ remains underexplored in the field of synthetic organic chemistry, in spite of holding great promise for the development of novel and valuable photo-induced transformations. Inspiration for the design of new catalytic modes originates from the throughout understanding of the fundamental reactivity principles of reactive intermediates. Recent work has demonstrated that the use of radicals generated from redox active precursors offers a convenient pathway to alkyl trifluoromethylation by interception of alkyl radical to CuCF_3_ complexes^40–42^. we wondered whether the Fe–N_3_ complexes were capable of a similar reactivity of trapping a radical intermediate. Based on iron catalysis and basic theories of photocatalysis^43^, we further envisioned that Fe–N_3_-based complex generated from readily available Fe salts and azido sources could be easily photoexcited by visible-light irradiation and subsequently could undergo Fe(III)−N_3_ homolysis to release an azide radical through LMCT process. The generated azide radical will readily add to carbon–carbon bond to provide a carbon radical intermediate, followed by interception of Fe(III)−N_3_ complex in analogy to recent reports in copper catalysis^40–42,44–46^(Fig. 1c).

Herein, we develop an effective strategy for alkene diazidation via iron-mediated LMCT mode, which provides a versatile platform to access structurally diverse diazides without external oxidants. This diazidation transformation proceeds under mild conditions and the reaction is characterized by its broad substrate scope, good functional group compatibility and operational simplicity.

## Results and discussion

Drawing inspiration from photo-induced vicinal dichlorination of alkenes through LMCT excitation of CuCl_2_^47^, where homolysis of an excited state CuCl_2_ could generate chlorine atom radicals, we first established the optimum reaction conditions, starting from the identification of the appropriate metal catalysts as shown in Table 1. We initially examined the application of CuCl_2_ to diazidation reaction of aliphatic alkene **1a** with TMSN_3_. However, none of the desired diazide product was obtained upon visible-light irradiation from blue LEDs (*λ*_max_ = 440 nm). Only vicinal dichlorination product was generated. Other copper salts such as CuBr, Cu(OAc)_2_ and Cu(acac)_2_ further were investigated, and couldn’t catalyze alkene diazidation reaction at all. We next screened a series of cobalt salts (e.g., CoBr_2_, Co(acac)_2_, CoCl_2_-dppe, Co(salen)Cl) or manganese salts (e.g., Mn(OAc)_2_, Mn(OTf)_2_, MnBr_2_, Mn(CO)_5_Br), respectively. However, no reaction occurred with these metal salts as the photocatalyst. We further studied iron salts and Fe(NO_3_)_3_·9H_2_O proved to be the ideal catalyst, delivering the desired diazide product **2a** in 80% isolated yield using CH_3_CN as solvent at room temperature under irradiation of a 40 W blue kessil light after 24 h (Table 1, entry 1). The use of other solvents such as CH_2_Cl_2_, dioxane completely inhibited this reaction (Table 1, entries 2 and 3) and EtOAc led to inferior reaction yield (Table 1, entry 4). The use of other azido sources such as TsN_3_ and CF_3_SO_2_N_3_ was useless (Table 1, entries 5 and 6). When 38 W White LEDs or 5 W blue LEDs replaced 40 W Blue kessil lamp as light source, this led to decreased reaction yield (Table 1, entries 7 and 8). Control experiments demonstrated that either Fe catalyst or light was essential for this olefin diazidation reaction (entries 9 and 10).

**Table 1:** Optimization of reaction conditions^a^



Entry	Variation from standard conditions	Yield^b^
1	None	84% (80%)
2	CH_2_Cl_2_ instead of MeCN	0%
3	dioxane instead of MeCN	0%
4	EtOAc instead of MeCN	53%
5	TsN_3_ instead of TMSN_3_	0%
6	CF_3_SO_2_N_3_ instead of TMSN_3_	0%
7	38 W White LEDs instead of 40 W Blue kessil lamp	44%
8	5 W blue LEDs instead of 40 W Blue kessil lamp	56%
9	Without Fe catalyst	0%
10	Without light	0%

*LEDs* light-emitting diodes.

^a^Standard reaction conditions: **1a** (0.2 mmol, 1.0 equiv.), TMSN_3_ (1.0 mmol, 5.0 equiv.), Fe(NO_3_)_3_·9H_2_O (0.24 mmol), MeCN (2 mL), Ar atmosphere, r.t., 24 h.

^b^Isolated yield.

**Fig. 2:**
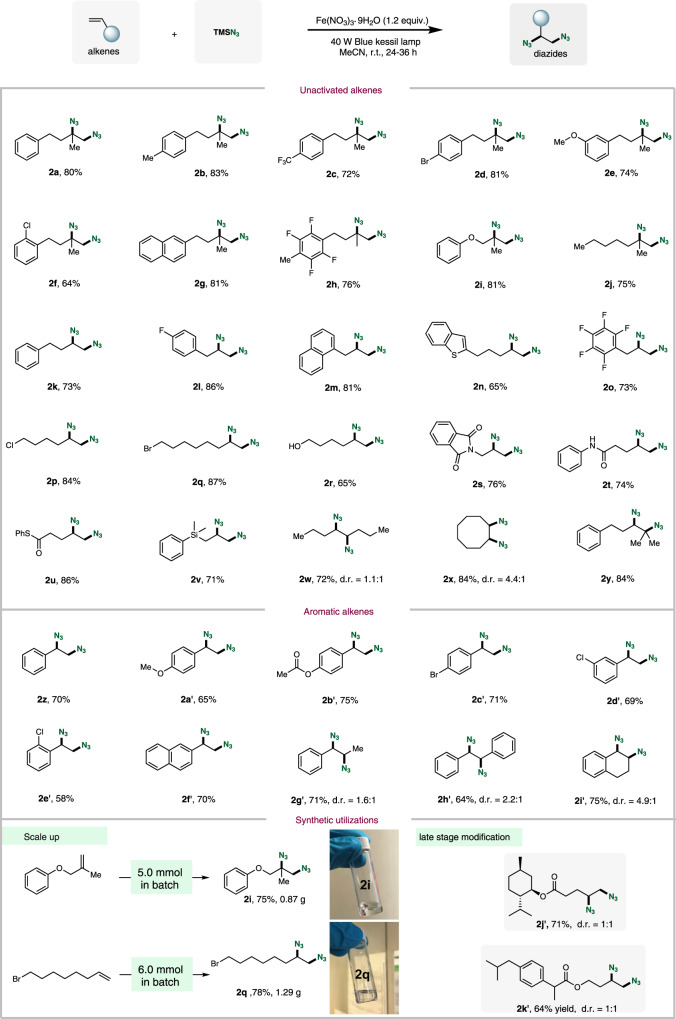
Scope of alkenes and synthetic utilization. Standard conditions: alkenes (0.20 mmol, 1.0 equiv), TMSN_3_ (1.0 mmol, 5.0 equiv), Fe(NO_3_)_3_·9H_2_O (0.24 mmol, 1.2 equiv) and MeCN (2 mL), blue LEDs, 24–36 h, isolated yield. d.r. diastereomeric ratios.

Having established optimized reaction conditions, we set out to explore the scope of alkenes for iron-mediated 1,2-diazidation reaction. A variety of diazide products were effectively synthesized in moderate to high yields from the corresponding alkenes with various functional groups compatible as shown in Fig. 2. First, 1,1-disubstituted type of alkenes were investigated under the optimized reaction conditions. For example, both electron-rich (-CH_3_, -MeO) and -electron-poor (-CF_3_) as well as halogen atoms (-Br, -Cl) substituents on the aromatic ring were well tolerated, and the corresponding diazides **2a**–**f** were obtained in good yields. It was found that the substituents of aryl ring on *ortho-*, *meta*-, and *para*-position on the phenyl rings can all work in this transformation. When the aryl ring in 1,1-disubstituted alkenes was replaced by other functional groups (e.g., naphthalene, 2,3,5,6-tetrafluoro-4-methylbenzene, phenol, alkyl group), the diazidation reaction could smoothly occur to provide the desired diazide products **2g**–**j**. Different substitutions (e.g., aromatics, benzothiophene, perfluoroaryl) in α-olefins were well tolerated to give the corresponding diazide products **2k**–**o** in good yields. The diazidation reaction can also tolerate various C(sp^3^)-bound functional groups, including halogen substituents (**2p**, **2q**), free alcohol (**2r**), phthalimide (**2s**), amide (**2t**), thioester (**2u**) and silyl group (**2v**). Internal as well as cyclic alkenes were performed, furnishing the corresponding diazides **2w** and **2x** with poor diastereomeric ratio. Trisubstituted alkene underwent diazidation reaction to generate the diazide **2y** in 84% yield. Various substituted styrenes were also assessed with regarding to this diazidation reaction, smoothly giving rise to the desired products **2z**–**i’** in good yield. Internal as well as cyclic styrenes smoothly delivered the vicinal diazide products **2g**–**i’** with low diastereoselectivity.

With a widespread exploration of the scope of alkenes in hand, we shifted our attention to the synthetic potential of this diazidation reaction. A gram-scale reaction was performed, and **2i** (5.0 mmol, 0.87 g, 75% yield) and **2q** (6.0 mmol, 1.29 g 78% yield) were obtained under the respective standard conditions as shown in Fig. 2. It is worth noting that this reaction was found to be broadly applicable to complex alkene substrates such as *L*-menthol and ibuprofen to deliver vicinal diazide products **2j’** and **2k’**, indicating the suitability of this strategy for late-stage modification.

**Fig. 3:**
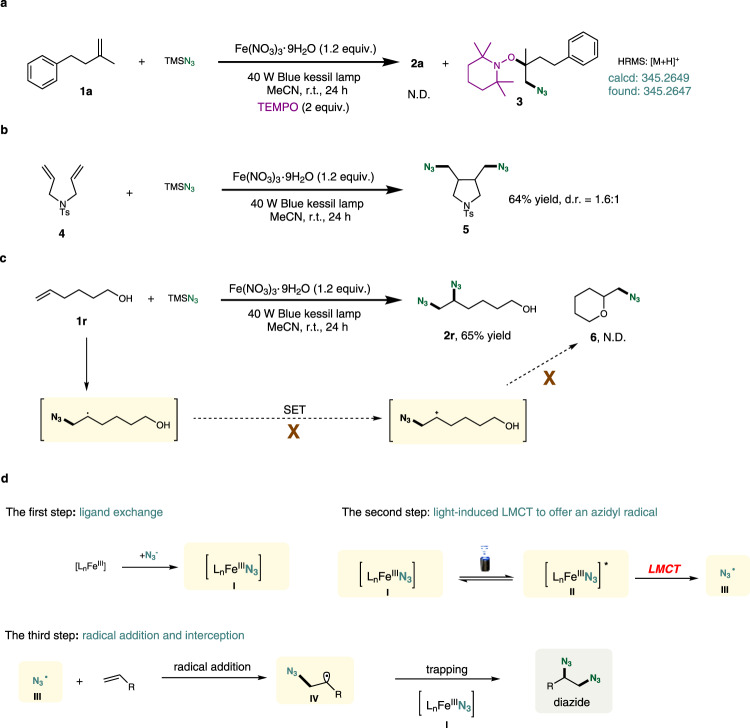
Mechanistic investigations and proposed reaction mechanism. **a** Radical trapping experiments. **b** Radical clock experiments. **c** Investigation of possible intermediates. **d** Plausible reaction mechanism. N.D. not detected.

A series of experimental studies were conducted to explore the mechanism of this olefin diazidation reaction (Fig. 3). First, the reaction of **1a** with TMSN_3_ was performed in the presence of a radical scavenger TEMPO (2,2,6,6-tetramethylpiperidine-1-oxyl), under otherwise identical to standard conditions (Fig. 3a). The reaction was completely inhibited without generation of the desired product **2a**. Interestingly, the compound **3** was successfully trapped by TEMPO with HRMS analysis, indicating the involvement of radical nature in the reaction process. In a radical clock experiment, *N*-tosyl diallylamine **4** underwent cyclization upon subjection to the standard condition, delivering radical addition/cyclization cascade product **5** in 64% yield with diastereomeric ratio of 1.6:1 (Fig. 3b). We subsequently conducted diazidation reaction using alkene **1r** decorated with free alcohol as the substrate for many times and no product **6** by trapping of a carbocation intermediate was observed by MS analysis, which suggested a radical-polar pathway was impossible. These experimental results further proved the reaction proceeded in a radical pathway.

Based on the above-mentioned control experiments, a plausible mechanism was presented in Fig. 3d. First, the key group transfer complex **I**, Fe^III^–N_3_, was formed through ligand exchange from Fe salt and TMSN_3_. The generated Fe^III^–N_3_ will become the excited state **II** by visible-light irradiation, providing an azidyl radical **III** through LMCT process. The azidyl radical **III** could undergo radical addition into alkene to furnish carbon radical intermediate **IV**, followed by interception of **I**. The iron-mediated azidyl radical transfer finally offered the desired diazides.

In summary, we have developed a mild and practical protocol for alkene diazidation via iron-mediated LMCT mode. The key group transfer agent, Fe^III^–N_3_, provides a novel pathway to generate an azidyl radical intermediate through LMCT process without the oxidation conditions. This protocol shows broad alkene scope with high functional group tolerance. Notably, the diazidation reaction represents a nice extension of iron photochemistry into synthetic organic chemistry.

## Methods

### General procedure for alkene diazidation reaction

A 10 mL Schlenk tube equipped with a magnetic stir bar was charged with Fe(NO_3_)_3_·9H_2_O (0.24 mmol, 100 mg). Then, the tube was evacuated and backfilled with Ar (three times). Alkenes (0.20 mmol, 1.0 equiv.) and TMSN_3_ (1.0 mmol) in CH_3_CN (2 mL) were added by syringe under Ar atmosphere. The reaction tube was then sealed and was placed at a distance (app. 5 cm) from a 40 W blue kessil lamp (Fig. S1). The reaction mixture was stirred for 24–36 h at room temperature. After the reaction, the resulting solution was filtered through a cotton plug and washed with EtOAc. The filtrate was removed under reduced pressure and the residue was purified by silica gel column chromatography (ethyl acetate/*n-*pentane) to afford desired diazides.

## Supplementary information

Supplementary information

Peer Review File

## Data Availability

The authors declare that all other data supporting the findings of this study are available within the article and Supplementary Information files, and also are available from the corresponding author upon request.

## References

[1] Lucet D, Le Gall T, Mioskowski C (1998). The chemistry of vicinal diamines. Angew. Chem. Int. Ed..

[2] Cardona F, Goti A (2009). Metal-catalysed 1, 2-diamination reactions. Nat. Chem..

[3] Makai S, Falk E, Morandi B (2020). Direct synthesis of unprotected 2-azidoamines from alkenes via an iron-catalyzed difunctionalization reaction. J. Am. Chem. Soc..

[4] Muniz K, Barreiro L, Romero RM, Martinez C (2017). Catalytic asymmetric diamination of styrenes. J. Am. Chem. Soc..

[5] Zhu Y, Cornwall RG, Du H, Zhao B, Shi Y (2014). Catalytic diamination of olefins via N–N bond activation. Acc. Chem. Res..

[6] Minisci F (1975). Free-radical additions to olefins in the presence of redox systems. Acc. Chem. Res..

[7] Yuan YA, Lu DF, Chen YR, Xu H (2016). Iron‐catalyzed direct diazidation for a broad range of olefins. Angew. Chem. Int. Ed..

[8] Kolb HC, Finn M, Sharpless KB (2001). Click chemistry: diverse chemical function from a few good reactions. Angew. Chem. Int. Ed..

[9] Hennessy ET, Betley TA (2013). Complex N-heterocycle synthesis via iron-catalyzed, direct C–H bond amination. Science.

[10] Jin L-M, Xu P, Xie J, Zhang XP (2020). Enantioselective intermolecular radical C–H amination. J. Am. Chem. Soc..

[11] Palacios F, Alonso C, Aparicio D, Rubiales G, Jesús M (2007). The aza-Wittig reaction: an efficient tool for the construction of carbon–nitrogen double bonds. Tetrahedron.

[12] Schilling CI, Jung N, Biskup M, Schepers U, Bräse S (2011). Bioconjugation via azide–Staudinger ligation: an overview. Chem. Soc. Rev..

[13] Arimoto M, Yamaguchi H, Fujita E, Nagao Y, Ochiai M (1989). Diazidation of allylsilanes with a combination of iodosylbenzene and trimethylsilyl azide, and synthesis of allyl azides. Chem. Pharm. Bull..

[14] Fristad WE, Brandvold TA, Peterson JR, Thompson SR (1985). Conversion of alkenes to 1, 2-diazides and 1, 2-diamines. J. Org. Chem..

[15] Fumagalli G, Rabet PT, Boyd S, Greaney MF (2015). Three‐component azidation of styrene‐type double bonds: light‐switchable behavior of a copper photoredox catalyst. Angew. Chem. Int. Ed..

[16] Liu W (2021). Iron-catalyzed enantioselective radical carboazidation and diazidation of α, β-unsaturated carbonyl compounds. J. Am. Chem. Soc..

[17] Lu M-Z, Wang C-Q, Loh T-P (2015). Copper-catalyzed vicinal oxyazidation and diazidation of styrenes under mild conditions: access to alkyl azides. Org. Lett..

[18] Shee, M. & Singh, N. P. Chemical versatility of azide radical: journey from a transient species to synthetic accessibility in organic transformations. *Chem. Soc. Rev*. **51**, 2255–2312 (2022). 10.1039/d1cs00494h 35229836

[19] Xu L, Chen J, Chu L (2019). Solvent-tuned chemoselective carboazidation and diazidation of alkenes via iron catalysis. Org. Chem. Front..

[20] Zhou H (2017). Copper-catalyzed ligand-free diazidation of olefins with TMSN_3_ in CH_3_CN or in H_2_O. Org. Lett..

[21] Lv D (2021). Iron‐catalyzed radical asymmetric aminoazidation and diazidation of styrenes. Angew. Chem. Int. Ed..

[22] Yan M, Kawamata Y, Baran PS (2017). Synthetic organic electrochemical methods since 2000: on the verge of a renaissance. Chem. Rev..

[23] Ma C (2021). Recent advances in organic electrosynthesis employing transition metal complexes as electrocatalysts. Sci. Bull..

[24] Savéant J-M (2008). Molecular catalysis of electrochemical reactions. Mechanistic aspects. Chem. Rev..

[25] Fu N, Sauer GS, Saha A, Loo A, Lin S (2017). Metal-catalyzed electrochemical diazidation of alkenes. Science.

[26] Siu JC, Parry JB, Lin S (2019). Aminoxyl-catalyzed electrochemical diazidation of alkenes mediated by a metastable charge-transfer complex. J. Am. Chem. Soc..

[27] Cai C-Y, Zheng Y-T, Li J-F, Xu H-C (2022). Cu-electrocatalytic diazidation of alkenes at ppm catalyst loading. J. Am. Chem. Soc..

[28] Li C-J, Anastas PT (2012). Green chemistry: present and future. Chem. Soc. Rev..

[29] Li C-J, Trost BM (2008). Green chemistry for chemical synthesis. Proc. Natl Acad. Sci. USA.

[30] Han J (2022). Photoinduced manganese-catalysed hydrofluorocarbofunctionalization of alkenes. Nat. Synth..

[31] Cheng L (2021). Iron-catalyzed arene C-H hydroxylation. Science.

[32] Liu L (2021). General method for iron-catalyzed multicomponent radical cascades–cross-couplings. Science.

[33] Ma W (2019). Iron-catalyzed anti-Markovnikov hydroamination and hydroamidation of allylic alcohols. J. Am. Chem. Soc..

[34] Wang Q (2022). Iron-catalysed reductive cross-coupling of glycosyl radicals for the stereoselective synthesis of C-glycosides. Nat. Synth..

[35] Kang YC, Treacy SM, Rovis T (2021). Iron-catalyzed photoinduced LMCT: A 1 °C–H abstraction enables skeletal rearrangements and C (sp^3^)–H alkylation. ACS Catal..

[36] Kjær KS (2019). Luminescence and reactivity of a charge-transfer excited iron complex with nanosecond lifetime. Science.

[37] Zhang Q (2022). Iron-catalyzed photoredox functionalization of methane and heavier gaseous alkanes: scope, kinetics, and computational studies. Org. Lett..

[38] Wang S (2022). Decarboxylative tandem C-N coupling with nitroarenes via SH2 mechanism. Nat. Commun..

[39] Hu A (2018). δ-Selective functionalization of alkanols enabled by visible-light-induced ligand-to-metal charge transfer. J. Am. Chem. Soc..

[40] Xiao H (2019). Copper-catalyzed late-stage benzylic C (sp3)–H trifluoromethylation. Chem.

[41] Guo S, AbuSalim DI, Cook SP (2018). Aqueous benzylic C–H trifluoromethylation for late-stage functionalization. J. Am. Chem. Soc..

[42] Xiao H, Shen H, Zhu L, Li C (2019). Copper-catalyzed radical aminotrifluoromethylation of alkenes. J. Am. Chem. Soc..

[43] Prier CK, Rankic DA, MacMillan DW (2013). Visible light photoredox catalysis with transition metal complexes: applications in organic synthesis. Chem. Rev..

[44] Zeng X (2019). Copper-catalyzed decarboxylative difluoromethylation. J. Am. Chem. Soc..

[45] Zeng X (2020). Copper-catalyzed deaminative difluoromethylation. Angew. Chem. Int. Ed..

[46] Zeng XJ (2019). Copper-catalyzed, chloroamide-directed benzylic C–H diflfluoromethylation. J. Am. Chem. Soc..

[47] Lian P, Long W, Li J, Zheng Y, Wan X (2020). Visible‐light‐induced vicinal dichlorination of alkenes through LMCT excitation of CuCl_2_. Angew. Chem. Int. Ed..

