# Supplementary material for: Iron-mediated ligand-to-metal charge transfer enables 1,2-diazidation of alkenes
Source: Nat Commun. 2022 Dec 23;13:7880. doi: 10.1038/s41467-022-35344-9 (PMC9789131; doi:10.1038/s41467-022-35344-9)

Supplementary information

Iron-mediated ligand-to-metal charge transfer enables 1,2-diazidation of alkenes

Zhang et al.

Table of Contents

1. Supplementary Methods..3

2. Supplementary Discussion...4

2.1 Reaction condition optimization...4

2.2 General procedure for alkene diazidation reaction...8

2.3 Synthetic utilizations..25

2.4. Mechanistic studies...28

3. Supplementary Notes……………………………………………………….31

3.1 Copies of ^{1}H NMR, ^{13}C NMR, ^{19}F NMR spectra..31

1. Supplementary Methods

All reactions were performed in flame-dried glassware using conventional Schlenk techniques under a static pressure of nitrogen unless stated otherwise. Liquids and solutions were transferred with syringes. Solvents were dried and purified following standard procedures. Technical grade solvents for extraction or chromatography (*n*-pentane, ethyl acetate, and ethanol) were distilled prior to use. Analytical thin layer chromatography (TLC) was performed on ALUGRAM® Xtra SIL G/UV_{254} TLC-Sheets by Macherey-Nagel. Flash column chromatography was performed on silica gel 60 (40-63 µm, 230-400 mesh, ASTM) by Grace using the indicated solvents. ^{1}H, ^{13}C, and ^{19}F NMR spectra were recorded in $CDCl_3$ on Bruker AV400 or AV500 instruments. Chemical shifts are reported to 0.01 ppm for ^{1}H NMR to 0.01 ppm for ^{13}C NMR and ^{19}F NMR spectra. Reference peaks for chloroform in ^{1}H NMR and ^{13}C NMR spectra were set at 7.26 ppm and 77.0 ppm, respectively. Data were reported as follows: chemical shift, multiplicity (s = singlet, d = doublet, t = triplet, q = quartet, m = multiplet), coupling constants (Hz), and integration. Gas liquid chromatography (GLC) was performed on an Agilent Technologies 7820A gas chromatograph equipped with a HP-5 capillary column (30 m × 0.32 mm, 0.25 µm film thickness) by Agilent Technologies/CS-Chromatographie Service using the following program: N_2 carrier gas, injection temperature 250 °C, detector temperature 300 °C, flow rate: 1.7 mL/min; temperature program: start temperature 40 °C, heating rate 10 °C/min, end temperature 280 °C for 10 min.

2. Supplementary Discussion

2.1 Reaction condition optimization

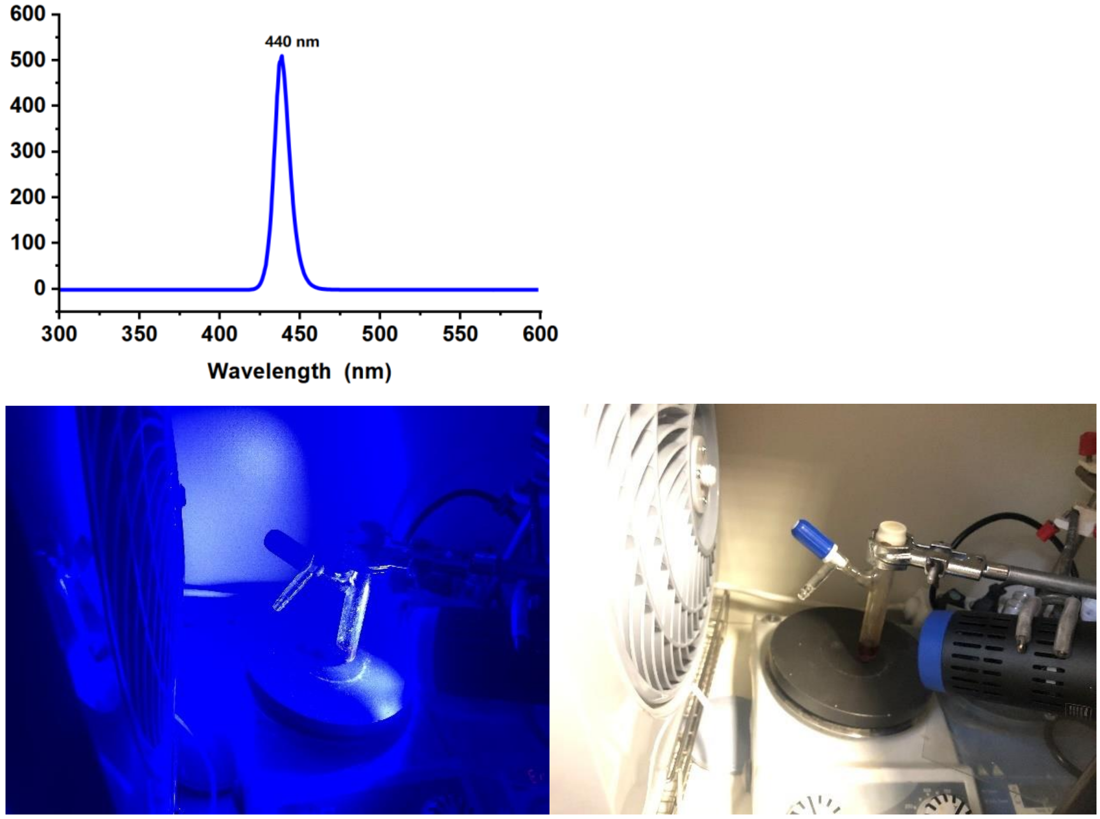

Supplementary Figure 1. Emission spectra of the blue LED light and typical reaction setup.

Supplementary Table 1. Cu catalyst screening

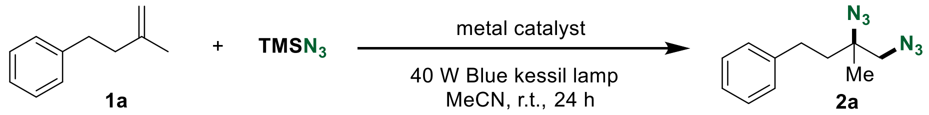

entry	copper salt	yield(%) [b]
1	$CuCl_2$	0
2	CuCl	0
3	$CuBr_2$	0
4	CuBr	0
5	$Cu(OAc)_2$	0
6	$Cu(acac)_2$	0
7	CuCN	0

[a] Reaction conditions: **1a** (0.20 mmol, 1.0 equiv), $TMSN_3$ (1.0 mmol, 5.0 equiv), Cu catalyst (0.24 mmol, 1.2 equiv) and CH_3CN (2 mL), blue

LEDs, 24 h. [b] Yields were determined by analysis of the crude ^1H NMR spectra using CH_2Br_2 as an internal standard.

Supplementary Table 2. Mn catalyst screening

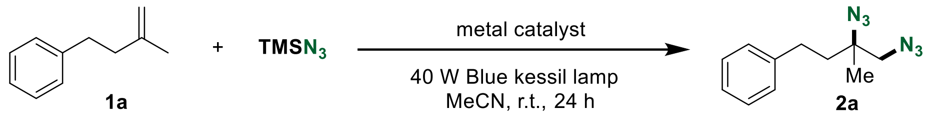

entry	manganese salt	yield(%) [b]
1	$Mn_2(CO)_{10}$	0
2	$Mn(CO)_5Br$	0
3	$MnBr_2$	0
4	$Mn(OAc)_3$	21
5	$Mn(OAc)_2$	0
6	$Mn(CO)_5I$	0
7	$Mn(OTf)_2$	0

[a] Reaction conditions: **1a** (0.20 mmol, 1.0 equiv), $TMSN_3$ (1.0 mmol, 5.0 equiv), Mn catalyst (0.24 mmol, 1.2 equiv) and CH_3CN (2 mL), blue LEDs, 24 h. [b] Yields were determined by analysis of the crude ^1H NMR spectra using CH_2Br_2 as an internal standard.

Supplementary Table 3. Co catalyst screening

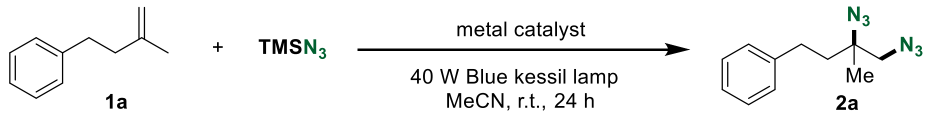

entry	cobalt salt	yield(%) [b]
1	$CoCl_2$	0
2	$CoBr_2$	0
3	CoI_2	0
4	$CoCl_2$·dppe	0
5	$Co(OAc)_2 \cdot 4H_2O$	0
6	Co(salen)Cl	0
7	$Cu(acac)_2$	0

[a] Reaction conditions: **1a** (0.20 mmol, 1.0 equiv), $TMSN_3$ (1.0 mmol, 5.0 equiv), Co catalyst (0.24 mmol, 1.2 equiv) and CH_3CN (2 mL), blue LEDs, 24 h. [b] Yields were determined by analysis of the crude ^1H

NMR spectra using CH_2Br_2 as an internal standard.

Supplementary Table 4. Fe catalyst screening

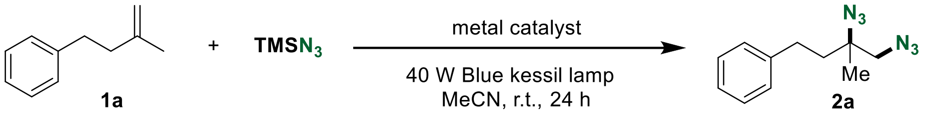

entry	iron salt	yield(%) [b]
1	$FeCl_3$	6
2	$Fe(acac)_3$	8
3	$Fe(NO_3)_3\cdot 9H_2O$	85
4	$FeCl_2$	5

[a] Reaction conditions: **1a** (0.20 mmol, 1.0 equiv), $TMSN_3$ (1.0 mmol, 5.0 equiv), Fe catalyst (0.24 mmol, 1.2 equiv) and CH_3CN (2 mL), blue LEDs, 24 h. [b] Yields were determined by analysis of the crude ^1H NMR spectra using CH_2Br_2 as an internal standard.

Supplementary Table 5. Solvent screening

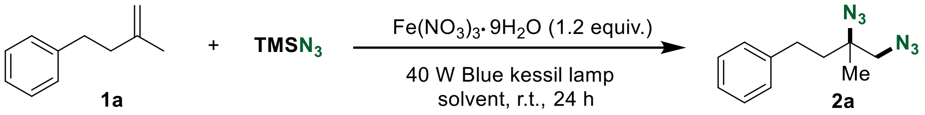

entry	Solvent (0.10 M)	yield(%) [b]
1	Et_2O	0
2	THF	0
3	CH_2Cl_2	0
4	$ClCH_2CH_2Cl$	0
5	EtOAc	67
7	Dioxane	0
8	CH_3CN	85

[a] Reaction conditions: **1a** (0.20 mmol, 1.0 equiv), $TMSN_3$ (1.0 mmol, 5.0 equiv), $Fe(NO_3)_3\cdot 9H_2O$ (0.24 mmol, 1.2 equiv) and solvent (2 mL), blue LEDs, 24 h. [b] Yields were determined by analysis of the crude ^1H NMR spectra using CH_2Br_2 as an internal standard.

Supplementary Table 6. Azido sources screening

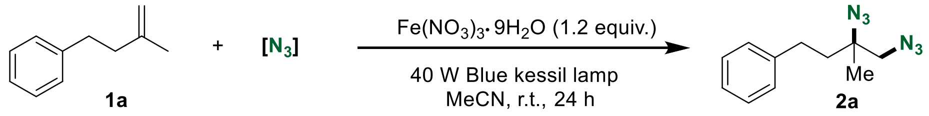

entry	azido sources	yield(%) ^{b}
1	TsN_3	0
2	$CF_3SO_2N_3$	0

[a] Reaction conditions: **1a** (0.20 mmol, 1.0 equiv), [N_3] (1.0 mmol, 5.0 equiv), $Fe(NO_3)_3 \cdot 9H_2O$ (0.24 mmol, 1.2 equiv) and solvent (2 mL), blue LEDs, 24 h. [b] Yields were determined by analysis of the crude ^{1}H NMR spectra using CH_2Br_2 as an internal standard.

Supplementary Table 7. Amount screening

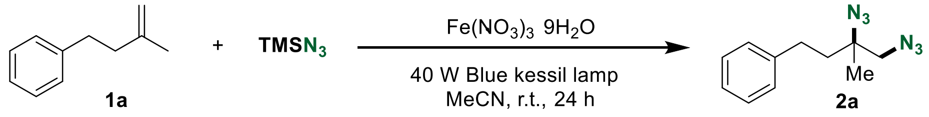

entry	Variation of standard conditions	yield(%) ^{b}
1	0.5 equiv. $Fe(NO_3)_3 \cdot 9H_2O$ was used	52
2	4.0 equiv. $TMSN_3$ was used	76
3	3.0 equiv. $TMSN_3$ was used	58
4	1 mL CH_3CN was used	76
5	4 mL CH_3CN was used	81

[a] Reaction conditions: **1a** (0.20 mmol, 1.0 equiv), $TMSN_3$ (1.0 mmol, 5.0 equiv), $Fe(NO_3)_3 \cdot 9H_2O$ (0.24 mmol, 1.2 equiv) and CH_3CN (2 mL), blue LEDs, 24 h. [b] Yields were determined by analysis of the crude ^{1}H NMR spectra using CH_2Br_2 as an internal standard.

Supplementary Table 8. Control experiments

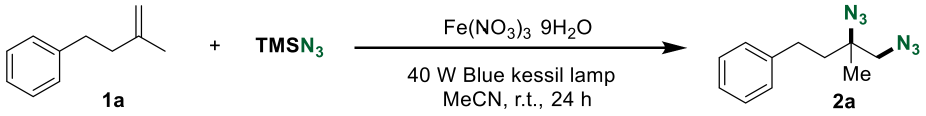

entry	deviation from standard conditions	yield(%) [b]
1	none	85(80)[c]
2	no Fe	0
3	no light	0

[a] Reaction conditions: **1a** (0.20 mmol, 1.0 equiv), $TMSN_3$ (1.0 mmol, 5.0 equiv), $Fe(NO_3)_3 \cdot 9H_2O$ (0.24 mmol, 1.2 equiv) and solvent (2 mL), blue LEDs, 24 h. [b] Yields were determined by analysis of the crude ^1H NMR spectra using CH_2Br_2 as an internal standard. [c] Isolated yield.

2.2 General procedure for alkene diazidation reaction

A 10 mL Schlenk tube equipped with a magnetic stir bar was charged with $Fe(NO_3)_3 \cdot 9H_2O$ (0.24 mmol, 100 mg). Then, the tube was evacuated and backfilled with Ar (three times). Alkenes (0.20 mmol, 1.0 equiv.) and $TMSN_3$ (1.0 mmol) in CH_3CN (2 mL) were added by syringe under Ar atmosphere. The reaction tube was then sealed and was placed at a distance (app. 5 cm) from a 40 W blue kessil lamp (Figure S1). The reaction mixture was stirred for 24-36 h at room temperature. After the reaction, the resulting solution was filtered through a cotton plug and washed with EtOAc. The filtrate was removed under reduced pressure and the residue was purified by silica gel column chromatography (ethyl acetate/*n*-pentane) to afford the desired diazides.

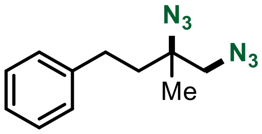

(3,4-diazido-3-methylbutyl)benzene

The reaction was carried out according to **the general alkene diazidation procedure** on 0.20-mmol scale (24 h). The residue was purified by flash column chromatography (ethyl acetate/*n*-pentane) to afford the diazide product **(3,4-diazido-3-methylbutyl)benzene** (**2a**) (36.8 mg, 80% yield) as an oil. **^{1}H NMR** (500 MHz, Chloroform-*d*) δ 7.32 – 7.28 (m, 2H), 7.24 – 7.18 (m, 3H), 3.39 – 3.28 (m, 2H), 2.68 (t, *J* = 8.2 Hz, 2H), 1.92 – 1.81 (m, 2H), 1.39 (s, 3H). **^{13}C NMR** (101 MHz, Chloroform-*d*) δ 141.05, 128.57, 128.26, 126.19, 63.54, 59.09, 39.41, 30.16, 21.30.

HRMS for $C_{11}H_{15}N_6^+$-N_2 (ESI) $[M]^+$: calc.: 203.1291, found: 203.1294.

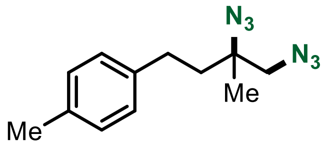

1-(3,4-diazido-3-methylbutyl)-4-methylbenzene

The reaction was carried out according to **the general alkene diazidation procedure** on 0.20-mmol scale (24 h). The residue was purified by flash column chromatography (ethyl acetate/*n*-pentane) to afford the diazide product **1-(3,4-diazido-3-methylbutyl)-4-methylbenzene** (**2b**) (40.6 mg, 83% yield) as an oil. **^{1}H NMR** (500 MHz, Chloroform-*d*) δ 7.12 (d, *J* = 7.9 Hz, 2H), 7.09 (d, *J* = 8.2 Hz, 2H), 3.36 – 3.28 (m, 2H), 2.68 – 2.62 (m, 2H), 2.33 (s, 3H), 1.92 – 1.78 (m, 2H), 1.38 (s, 3H). **^{13}C NMR** (101 MHz, Chloroform-*d*) δ 137.95, 135.70, 129.24, 128.12, 63.56, 59.09, 39.55, 29.70, 21.28, 20.96.

HRMS for $C_{12}H_{17}N_6^+$-N_2 (ESI) $[M]^+$: calc.: 217.1448, found: 217.1449.

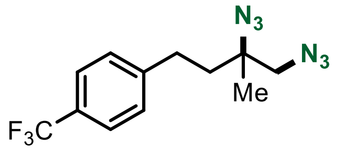

1-(3,4-diazido-3-methylbutyl)-4-(trifluoromethyl)benzene

The reaction was carried out according to **the general alkene diazidation procedure** on 0.20-mmol scale (24 h). The residue was purified by flash column chromatography (ethyl acetate/*n*-pentane) to afford the diazide product **1-(3,4-diazido-3-**

methylbutyl)-4-(trifluoromethyl)benzene (**2c**) (42.9 mg, 72% yield) as an oil. **^{1}H NMR** (500 MHz, Chloroform-*d*) δ 7.56 (d, *J* = 8.0 Hz, 2H), 7.31 (d, *J* = 7.9 Hz, 2H), 3.35 (s, 2H), 2.74 (ddd, *J* = 9.7, 6.9, 2.1 Hz, 2H), 1.92 – 1.78 (m, 2H), 1.40 (s, 3H). **^{13}C NMR** (101 MHz, Chloroform-*d*) δ 145.18, 128.67 (q, *J* = 32.4 Hz), 128.62, 125.51 (q, *J* = 3.9 Hz), 124.16 (q, *J* = 272.0 Hz), 63.36, 59.05, 39.08, 30.02, 21.29. **^{19}F NMR** (471 MHz, Chloroform-*d*) δ -62.41. **HRMS** for $C_{12}H_{14}F_3N_6^+$-N_2 (ESI) $[M]^+$: calc.: 271.1165, found: 271.1166.

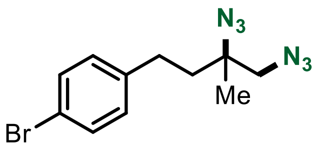

1-bromo-4-(3,4-diazido-3-methylbutyl)benzene

The reaction was carried out according to **the general alkene diazidation procedure** on 0.20-mmol scale (24 h). The residue was purified by flash column chromatography (ethyl acetate/*n*-pentane) to afford the diazide product **1-bromo-4-(3,4-diazido-3-methylbutyl)benzene** (**2d**) (50.1 mg, 81% yield) as an oil. **^{1}H NMR** (500 MHz, Chloroform-*d*) δ 7.42 (d, *J* = 8.4 Hz, 2H), 7.07 (d, *J* = 8.4 Hz, 2H), 3.32 (d, *J* = 1.4 Hz, 2H), 2.63 (t, *J* = 8.7 Hz, 2H), 1.93 – 1.72 (m, 2H), 1.38 (s, 3H). **^{13}C NMR** (101 MHz, Chloroform-*d*) δ 140.01, 131.64, 130.02, 119.97, 63.40, 59.06, 39.22, 29.59, 21.30.

HRMS for $C_{11}H_{14}BrN_6^+$-N_2 (ESI) $[M]^+$: calc.: 281.0396, found: 281.0396.

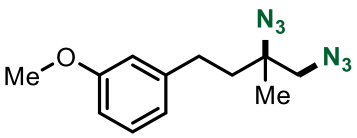

1-(3,4-diazido-3-methylbutyl)-3-methoxybenzene

The reaction was carried out according to **the general alkene diazidation procedure** on 0.20-mmol scale (24 h). The residue was purified by flash column chromatography (ethyl acetate/*n*-pentane) to afford the diazide product **1-(3,4-diazido-3-methylbutyl)-3-methoxybenzene** (**2e**) (38.5 mg, 74% yield) as an oil. **^{1}H NMR** (500 MHz, Chloroform-*d*) δ 7.22 (t, *J* = 7.8 Hz, 1H), 6.81 – 6.73 (m, 3H), 3.81 (s, 3H),

3.37 – 3.28 (m, 2H), 2.65 (ddd, J = 9.5, 7.8, 1.9 Hz, 2H), 1.92 – 1.78 (m, 2H), 1.38 (s, 3H). **^{13}C NMR** (101 MHz, Chloroform-d) δ 159.81, 142.68, 129.56, 120.63, 114.12, 111.47, 63.53, 59.09, 55.17, 39.31, 30.21, 21.29.

HRMS for $C_{12}H_{17}N_6O^+$-N_2 (ESI) $[M]^+$: calc.: 233.1397, found: 233.1393.

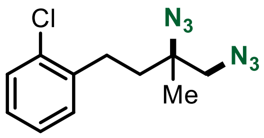

1-chloro-2-(3,4-diazido-3-methylbutyl)benzene

The reaction was carried out according to **the general alkene diazidation procedure** on 0.20-mmol scale (24 h). The residue was purified by flash column chromatography (ethyl acetate/n-pentane) to afford the diazide product **1-chloro-2-(3,4-diazido-3-methylbutyl)benzene** (**2f**) (33.9 mg, 64% yield) as an oil. **^{1}H NMR** (500 MHz, Chloroform-d) δ 7.35 (d, J = 7.5 Hz, 1H), 7.25 – 7.14 (m, 3H), 3.40 – 3.31 (m, 2H), 2.87 – 2.74 (m, 2H), 1.89 – 1.79 (m, 2H), 1.41 (s, 3H). **^{13}C NMR** (101 MHz, Chloroform-d) δ 138.66, 133.78, 130.35, 129.63, 127.78, 127.07, 63.49, 59.05, 37.58, 28.17, 21.15.

HRMS for $C_{11}H_{14}ClN_6^+$-N_2 (ESI) $[M]^+$: calc.: 237.0902, found: 237.0906.

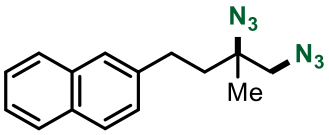

2-(3,4-diazido-3-methylbutyl)naphthalene

The reaction was carried out according to **the general alkene diazidation procedure** on 0.20-mmol scale (36 h). The residue was purified by flash column chromatography (ethyl acetate/n-pentane) to afford the diazide product **2-(3,4-diazido-3-methylbutyl)naphthalene** (**2g**) (45.4 mg, 81% yield) as an oil. **^{1}H NMR** (500 MHz, Chloroform-d) δ 7.84 – 7.77 (m, 3H), 7.64 (s, 1H), 7.49 – 7.40 (m, 2H), 7.33 (d, J = 9.6 Hz, 1H), 3.40 – 3.32 (m, 2H), 2.85 (t, J = 8.7 Hz, 2H), 2.00 – 1.88 (m, 2H), 1.42 (s, 3H). **^{13}C NMR** (101 MHz, Chloroform-d) δ 138.51, 133.63, 132.10, 128.21, 127.63, 127.39, 126.95, 126.36, 126.09, 125.39, 63.59, 59.13, 39.32, 30.33, 21.34.

HRMS for $C_{15}H_{17}N_6^+$-N_2 (ESI) $[M]^+$: calc.: 253.1448, found: 253.1449.

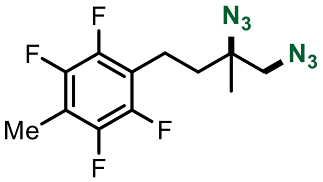

1-(3,4-diazido-3-methylbutyl)-2,3,5,6-tetrafluoro-4-methylbenzene

The reaction was carried out according to **the general alkene diazidation procedure** on 0.20-mmol scale (24 h). The residue was purified by flash column chromatography (ethyl acetate/*n*-pentane) to afford the diazide product **1-(3,4-diazido-3-methylbutyl)-2,3,5,6-tetrafluoro-4-methylbenzene** (**2h**) (48.0 mg, 76% yield) as an oil. **^{1}H NMR** (500 MHz, Chloroform-*d*) δ 3.39 – 3.32 (m, 2H), 2.77 (ddd, *J* = 9.2, 7.4, 1.5 Hz, 2H), 2.24 (t, *J* = 2.1 Hz, 3H), 1.84 – 1.75 (m, 2H), 1.40 (s, 3H). **^{13}C NMR** (101 MHz, Chloroform-*d*) δ 146.33 – 145.51 (m), 143.95 – 143.01 (m), 116.24 (t, *J* = 18.3 Hz), 114.08 (t, *J* = 19.1 Hz), 63.13, 58.99, 36.68, 31.24, 20.96, 17.24. **^{19}F NMR** (471 MHz, Chloroform-*d*) δ -144.04 – -144.93 (m), -146.63 (dd, *J* = 22.9, 13.5 Hz). **HRMS** for $C_{12}H_{13}F_4N_6^+$-N_2 (ESI) $[M]^+$: calc.: 289.1071, found: 289.1075.

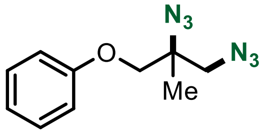

(2,3-diazido-2-methylpropoxy)benzene

The reaction was carried out according to **the general alkene diazidation procedure** on 0.20-mmol scale (24 h). The residue was purified by flash column chromatography (ethyl acetate/*n*-pentane) to afford the diazide product **(2,3-diazido-2-methylpropoxy)benzene** (**2i**) (35.3 mg, 81% yield) as an oil. **^{1}H NMR** (500 MHz, Chloroform-*d*) δ 7.31 (dd, *J* = 8.6, 7.4 Hz, 2H), 7.00 (t, *J* = 7.4 Hz, 1H), 6.93 (d, *J* = 8.7 Hz, 2H), 3.99 – 3.93 (m, 2H), 3.54 – 3.46 (m, 2H), 1.46 (s, 3H). **^{13}C NMR** (101 MHz, Chloroform-*d*) δ 158.07, 129.57, 121.61, 114.62, 71.33, 62.85, 56.26, 19.25. **HRMS** for $C_{10}H_{13}N_6O^+$-N_2 (ESI) $[M]^+$: calc.: 205.1084, found: 205.1086.

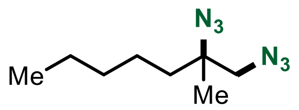

1,2-diazido-2-methylheptane

The reaction was carried out according to **the general alkene diazidation procedure** on 0.20-mmol scale (24 h). The residue was purified by flash column chromatography (ethyl acetate/*n*-pentane) to afford the diazide product **1,2-diazido-2-methylheptane** (**2j**) (29.4 mg, 75% yield) as an oil. **^{1}H NMR** (500 MHz, Chloroform-*d*) δ 3.29 – 3.21 (m, 2H), 1.57 – 1.52 (m, 2H), 1.38 – 1.25 (m, 9H), 0.90 (t, *J* = 6.9 Hz, 3H). **^{13}C NMR** (101 MHz, Chloroform-*d*) δ 63.82, 59.11, 37.33, 31.97, 23.33, 22.46, 21.22, 13.92.
HRMS for $C_8H_{17}N_6^+$-N_2 (ESI) [M]$^+$: calc.: 169,1448, found: 169,1449.

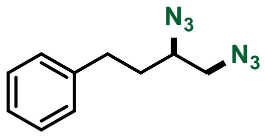

3,4-diazidobutyl)benzene

The reaction was carried out according to **the general alkene diazidation procedure** on 0.20-mmol scale (24 h). The residue was purified by flash column chromatography (ethyl acetate/*n*-pentane) to afford the diazide product **3,4-diazidobutyl)benzene** (**2k**) (31.5 mg, 73% yield) as an oil. **^{1}H NMR** (500 MHz, Chloroform-*d*) δ 7.31 (t, *J* = 7.5 Hz, 2H), 7.21 (dd, *J* = 18.8, 7.2 Hz, 3H), 3.48 – 3.33 (m, 3H), 2.85 – 2.78 (m, 1H), 2.75 – 2.67 (m, 1H), 1.91 – 1.80 (m, 2H). **^{13}C NMR** (101 MHz, Chloroform-*d*) δ 140.35, 128.65, 128.37, 126.34, 61.13, 54.94, 33.40, 32.01.
HRMS for $C_{10}H_{13}N_6^+$-N_2 (ESI) [M]$^+$: calc.: 189.1135, found: 189.1138.

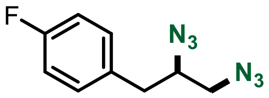

1-(2,3-diazidopropyl)-4-fluorobenzene

The reaction was carried out according to **the general alkene diazidation procedure** on 0.20-mmol scale (24 h). The residue was purified by flash column chromatography (ethyl acetate/*n*-pentane) to afford the diazide product **1-(2,3-diazidopropyl)-4-fluorobenzene** (**2l**) (37.8 mg, 86% yield) as an oil. **^{1}H NMR** (500 MHz, Chloroform-

d) δ 7.19 (dd, *J* = 8.7, 5.3 Hz, 2H), 7.03 (t, *J* = 8.7 Hz, 2H), 3.71 – 3.65 (m, 1H), 3.40 (dd, *J* = 12.7, 4.2 Hz, 1H), 3.30 (dd, *J* = 12.7, 6.8 Hz, 1H), 2.90 – 2.79 (m, 2H). **^{13}C NMR** (101 MHz, Chloroform-*d*) δ 162.01 (d, *J* = 245.8 Hz), 132.02 (d, *J* = 3.7 Hz), 130.76 (d, *J* = 7.6 Hz), 115.67 (d, *J* = 21.6 Hz), 62.88, 53.85, 37.19. **^{19}F NMR** (471 MHz, Chloroform-*d*) δ -115.41.

HRMS for $C_9H_{10}FN_6^+$-N_2 (ESI) $[M]^+$: calc.: 193.0884, found: 193.0886.

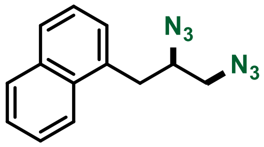

1-(2,3-diazidopropyl)naphthalene

The reaction was carried out according to **the general alkene diazidation procedure** on 0.20-mmol scale (24 h). The residue was purified by flash column chromatography (ethyl acetate/*n*-pentane) to afford the diazide product **1-(2,3-diazidopropyl)naphthalene** (**2m**) (40.8 mg, 81% yield) as an oil. **^{1}H NMR** (500 MHz, Chloroform-*d*) δ 7.99 (d, *J* = 8.4 Hz, 1H), 7.90 (d, *J* = 8.0 Hz, 1H), 7.81 (d, *J* = 8.1 Hz, 1H), 7.60 – 7.50 (m, 2H), 7.46 – 7.38 (m, 2H), 3.91 (qd, *J* = 7.0, 4.1 Hz, 1H), 3.44 (dd, *J* = 12.7, 4.1 Hz, 1H), 3.40 – 3.32 (m, 3H). **^{13}C NMR** (101 MHz, Chloroform-*d*) δ 134.01, 132.39, 131.70, 129.11, 128.12, 127.96, 126.48, 125.85, 125.50, 123.02, 61.97, 54.13, 35.13.

HRMS for $C_{13}H_{13}N_6^+$-N_2 (ESI) $[M]^+$: calc.: 225.1135, found: 225.1138.

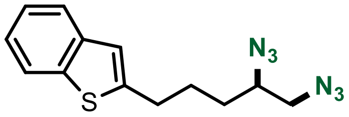

2-(4,5-diazidopentyl)benzo[*b*]thiophene

The reaction was carried out according to **the general alkene diazidation procedure** on 0.20-mmol scale (24 h). The residue was purified by flash column chromatography (ethyl acetate/*n*-pentane) to afford the diazide product **2-(4,5-diazidopentyl)benzo[b]thiophene** (**2n**) (37.2 mg, 65% yield) as an oil. **^{1}H NMR** (500 MHz, Chloroform-*d*) δ 7.77 (d, *J* = 8.3 Hz, 1H), 7.68 (d, *J* = 7.7 Hz, 1H), 7.34 –

7.30 (m, 1H), 7.29 – 7.25 (m, 1H), 7.02 (s, 1H), 3.50 (dt, *J* = 12.5, 5.5 Hz, 1H), 3.39 (dd, *J* = 12.7, 4.1 Hz, 1H), 3.33 (dd, *J* = 12.7, 7.3 Hz, 1H), 2.96 (t, *J* = 7.7 Hz, 2H), 1.98 – 1.90 (m, 1H), 1.88 – 1.78 (m, 1H), 1.67 – 1.59 (m, 2H). **^{13}C NMR** (101 MHz, Chloroform-*d*) δ 145.06, 140.06, 139.30, 124.19, 123.63, 122.80, 122.15, 121.01, 61.82, 54.84, 31.11, 30.32, 27.29.

HRMS for $C_{13}H_{15}N_6S^+$-N_2 (ESI) [M]$^+$: calc.: 259.1012, found: 259.1013.

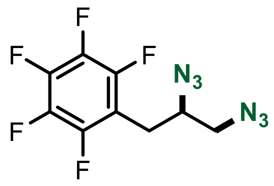

1-(2,3-diazidopropyl)-2,3,4,5,6-pentafluorobenzene

The reaction was carried out according to **the general alkene diazidation procedure** on 0.20-mmol scale (24 h). The residue was purified by flash column chromatography (ethyl acetate/*n*-pentane) to afford the diazide product **1-(2,3-diazidopropyl)-2,3,4,5,6-pentafluorobenzene** (**2o**) (42.6 mg, 73% yield) as an oil. **^{1}H NMR** (500 MHz, Chloroform-*d*) δ 3.80 – 3.74 (m, 1H), 3.49 (dd, *J* = 12.8, 4.4 Hz, 1H), 3.43 (dd, *J* = 12.8, 7.0 Hz, 1H), 2.94 (dt, *J* = 7.0, 1.7 Hz, 2H). **^{13}C NMR** (101 MHz, Chloroform-*d*) δ 149.33 – 140.82 (m), 140.13 – 137.24 (m), 110.23 – 109.79 (m), 60.61, 54.23, 25.17. **^{19}F NMR** (471 MHz, Chloroform-*d*) δ -142.20 (dd, *J* = 23.6, 10.1 Hz), -154.68 (t, *J* = 21.2 Hz), -158.60 – -170.67 (m).

HRMS for $C_9H_6F_5N_6^+$-N_2 (ESI) [M]$^+$: calc.: 265.0507, found: 265.0509.

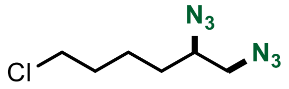

1,2-diazido-6-chlorohexane

The reaction was carried out according to **the general alkene diazidation procedure** on 0.20-mmol scale (24 h). The residue was purified by flash column chromatography (ethyl acetate/*n*-pentane) to afford the diazide product **1,2-diazido-6-chlorohexane** (**2p**) (33.9 mg, 84% yield) as an oil. **^{1}H NMR** (500 MHz, Chloroform-*d*) δ 3.55 (t, *J* = 6.5 Hz, 2H), 3.48 (ddd, *J* = 8.5, 3.9, 2.0 Hz, 1H), 3.43 – 3.39 (m, 1H), 3.34 (dd, *J* =

12.6, 7.2 Hz, 1H), 1.85 – 1.78 (m, 2H), 1.65 – 1.49 (m, 4H). **^{13}C NMR** (101 MHz, Chloroform-*d*) δ 61.82, 54.76, 44.45, 32.10, 31.07, 23.26.

HRMS for $C_6H_{12}ClN_6^+$-N_2 (ESI) [M]$^+$: calc.: 175.0745, found: 175.0749.

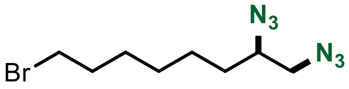

1,2-diazido-8-bromooctane

The reaction was carried out according to **the general alkene diazidation procedure** on 0.20-mmol scale (24 h). The residue was purified by flash column chromatography (ethyl acetate/*n*-pentane) to afford the diazide product **1,2-diazido-8-bromooctane** (**2q**) (47.9 mg, 87% yield) as an oil. **^{1}H NMR** (500 MHz, Chloroform-*d*) δ 3.49 – 3.43 (m, 1H), 3.43 – 3.37 (m, 3H), 3.32 (dd, *J* = 12.6, 7.3 Hz, 1H), 1.90 – 1.83 (m, 2H), 1.58 – 1.52 (m, 2H), 1.51 – 1.33 (m, 6H). **^{13}C NMR** (101 MHz, Chloroform-*d*) δ 61.96, 54.82, 33.68, 31.66, 28.43, 27.90, 25.71.

HRMS for $C_8H_{16}BrN_6^+$-N_2 (ESI) [M]$^+$: calc.: 247.0553, found: 247.0555.

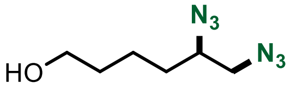

5,6-diazidohexan-1-ol

The reaction was carried out according to **the general alkene diazidation procedure** on 0.20-mmol scale (24 h). The residue was purified by flash column chromatography (ethyl acetate/*n*-pentane) to afford the diazide product **5,6-diazidohexan-1-ol** (**2r**) (23.9 mg, 65% yield) as an oil. **^{1}H NMR** (400 MHz, Chloroform-*d*) δ 3.68 (t, *J* = 6.0 Hz, 2H), 3.49 (d, *J* = 13.2 Hz, 1H), 3.42 – 3.38 (m, 1H), 3.36 – 3.31 (m, 1H), 2.04 (br s, 1H), 1.65 – 1.56 (m, 6H). **^{13}C NMR** (101 MHz, Chloroform-*d*) δ 62.51, 61.98, 54.80, 32.19, 31.56, 22.25.

HRMS for $C_6H_{13}N_6O^+$-N_2 (ESI) [M]$^+$: calc.: 157.1084, found: 157.1084.

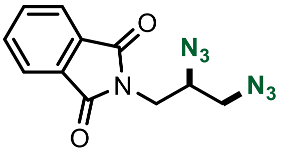

2-(2,3-diazidopropyl)isoindoline-1,3-dione

The reaction was carried out according to **the general alkene diazidation procedure** on 0.20-mmol scale (36 h). The residue was purified by flash column chromatography (ethyl acetate/*n*-pentane) to afford the diazide product **2-(2,3-diazidopropyl)isoindoline-1,3-dione** (**2s**) (38.5 mg, 76% yield) as an oil. **^{1}H NMR** (500 MHz, Chloroform-*d*) δ 7.89 (dd, *J* = 5.5, 3.1 Hz, 2H), 7.76 (dd, *J* = 5.5, 3.1 Hz, 2H), 3.98 – 3.92 (m, 1H), 3.88 (dd, *J* = 14.0, 7.4 Hz, 1H), 3.77 (dd, *J* = 13.9, 5.5 Hz, 1H), 3.54 (dd, *J* = 12.9, 4.2 Hz, 1H), 3.44 (dd, *J* = 12.9, 7.1 Hz, 1H). **^{13}C NMR** (101 MHz, Chloroform-*d*) δ 167.94, 134.37, 131.72, 123.64, 59.61, 52.94, 38.90.
HRMS for $C_{11}H_{10}N_7O_2^+$-N_2 (ESI) $[M]^+$: calc.: 244.0829, found: 244.0829.

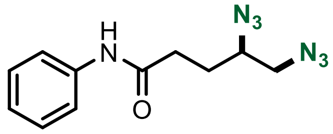

4,5-diazido-*N*-phenylpentanamide

The reaction was carried out according to **the general alkene diazidation procedure** on 0.20-mmol scale (36 h). The residue was purified by flash column chromatography (ethyl acetate/*n*-pentane) to afford the diazide product **4,5-diazido-N-phenylpentanamide** (**2t**) (38.3 mg, 74% yield) as an oil. ^{1}H NMR (400 MHz, Chloroform-*d*) δ 7.54 – 7.45 (m, 2H), 7.36 – 7.28 (m, 3H), 7.18 – 7.06 (m, 1H), 3.67 – 3.60 (m, 1H), 3.49 (dd, *J* = 12.8, 4.0 Hz, 1H), 3.38 (dd, *J* = 12.7, 7.3 Hz, 1H), 2.52 (ddt, *J* = 17.7, 15.5, 7.4 Hz, 2H), 2.04 (dtd, *J* = 15.0, 7.6, 4.0 Hz, 1H), 1.88 – 1.74 (m, 1H). ^{13}C NMR (126 MHz, Chloroform-*d*) δ 169.60, 137.51, 129.05, 124.55, 119.89, 61.13, 54.97, 33.17, 27.28.
HRMS for $C_{11}H_{14}N_7O^+$-N_2 (ESI) $[M]^+$: calc.: 232.1193, found: 232.1198.

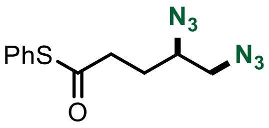

S-phenyl-4,5-diazidopentanethioate

The reaction was carried out according to **the general alkene diazidation procedure** on 0.20-mmol scale (36 h). The residue was purified by flash column chromatography (ethyl acetate/*n*-pentane) to afford the diazide product **S-phenyl-4,5-**

diazidopentanethioate (**2u**) (47.5 mg, 86% yield) as an oil. **^{1}H NMR** (500 MHz, Chloroform-*d*) δ 7.42 (d, *J* = 1.3 Hz, 5H), 3.64 – 3.57 (m, 1H), 3.45 (dd, *J* = 12.8, 4.2 Hz, 1H), 3.36 (dd, *J* = 12.7, 7.2 Hz, 1H), 2.83 (t, *J* = 7.1 Hz, 2H), 2.02 – 1.93 (m, 1H), 1.85 – 1.76 (m, 1H).**^{13}C NMR** (101 MHz, Chloroform-*d*) δ 196.46, 134.46, 129.60, 129.28, 127.22, 60.86, 54.85, 39.37, 27.34.

HRMS for $C_{11}H_{13}N_6OS^+$-N_2 (ESI) $[M]^+$: calc.: 249.0805, found: 249.0809.

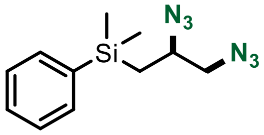

(2,3-diazidopropyl)dimethyl(phenyl)silane

The reaction was carried out according to **the general alkene diazidation procedure** on 0.20-mmol scale (24 h). The residue was purified by flash column chromatography (ethyl acetate/*n*-pentane) to afford the diazide product **(2,3-diazidopropyl)dimethyl(phenyl)silane** (**2v**) (36.9 mg, 71% yield) as an oil. **^{1}H NMR** (400 MHz, Chloroform-*d*) δ 7.54 – 7.49 (m, 2H), 7.41 – 7.36 (m, 3H), 3.51 – 3.43 (m, 1H), 3.29 (dd, *J* = 12.6, 3.8 Hz, 1H), 3.21 (dd, *J* = 12.6, 7.3 Hz, 1H), 1.20 – 0.99 (m, 2H), 0.40 (s, 3H), 0.38 (s, 3H). **^{13}C NMR** (126 MHz, Chloroform-*d*) δ 137.37, 133.44, 129.46, 128.08, 59.54, 57.03, 19.16, -2.35, -2.87.

HRMS for $C_{11}H_{17}N_6Si^+$-N_2 (ESI) $[M]^+$: calc.: 233.1217, found: 233.1219.

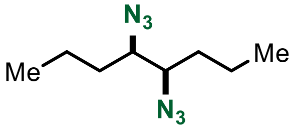

4,5-diazidooctane

The reaction was carried out according to **the general alkene diazidation procedure** on 0.20-mmol scale (24 h). The residue was purified by flash column chromatography (ethyl acetate/*n*-pentane) to afford the diazide product **4,5-diazidooctane** (**2w**) (28.6 mg, 72% yield, d.r. = 1.1:1) as an oil. **^{1}H NMR** (500 MHz, Chloroform-*d*) δ 3.37 – 3.32 (m, 1H), 3.30 – 3.25 (m, 1H), 1.69 – 1.50 (m, 6H), 1.46 – 1.37 (m, 2H), 1.02 – 0.94 (m, 6H). **^{13}C NMR** (101 MHz, Chloroform-*d*) δ 65.63, 65.03, 33.36, 32.45,

19.59, 19.47, 13.79.

HRMS for $C_8H_{17}N_6^+$-N_2 (ESI) $[M]^+$: calc.: 169.1448, found: 169.1445.

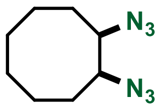

1,2-diazidocyclooctane

The reaction was carried out according to **the general alkene diazidation procedure** on 0.20-mmol scale (24 h). The residue was purified by flash column chromatography (ethyl acetate/*n*-pentane) to afford the diazide product **1,2-diazidocyclooctane** (**2x**) (32.6 mg, 84% yield, d.r. = 4.4:1) as an oil. **^{1}H NMR** (500 MHz, Chloroform-*d*) δ 3.75 (dd, *J* = 8.1, 3.8 Hz, 0.37H), 3.50 (dd, *J* = 4.8, 2.2 Hz, 1.63H), 1.98 – 1.90 (m, 2H), 1.84 – 1.70 (m, 4H), 1.69 – 1.62 (m, 2H), 1.61 – 1.50 (m, 2H), 1.48 – 1.38 (m, 2H). **^{13}C NMR** (101 MHz, Chloroform-*d*) δ 66.57, 63.43, 29.27, 28.14, 26.45, 25.59, 24.72, 23.47.

HRMS for $C_8H_{15}N_6^+$-N_2 (ESI) $[M]^+$: calc.: 167.1291, found: 167.1292.

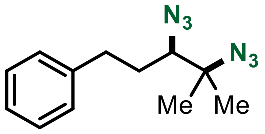

(3,4-diazido-4-methylpentyl)benzene

The reaction was carried out according to **the general alkene diazidation procedure** on 0.20-mmol scale (24 h). The residue was purified by flash column chromatography (ethyl acetate/*n*-pentane) to afford the diazide product **(3,4-diazido-4-methylpentyl)benzene** (**2y**) (50.0 mg, 84% yield) as an oil. **^{1}H NMR** (500 MHz, Chloroform-*d*) δ 7.33 (d, *J* = 7.8 Hz, 2H), 7.24 – 7.19 (m, 3H), 3.07 (dd, *J* = 11.2, 2.3 Hz, 1H), 2.94 (ddd, *J* = 13.8, 9.3, 4.7 Hz, 1H), 2.67 (ddd, *J* = 13.8, 8.9, 7.8 Hz, 1H), 1.98 – 1.90 (m, 1H), 1.77 – 1.70 (m, 1H), 1.31 (s, 3H), 1.28 (s, 3H). **^{13}C NMR** (101 MHz, Chloroform-*d*) δ 140.63, 128.44, 126.27, 69.73, 64.57, 33.05, 31.30, 22.95, 22.83.

HRMS for $C_{12}H_{17}N_6^+$-N_2 (ESI) $[M]^+$: calc.: 217.1448, found: 217.1445.

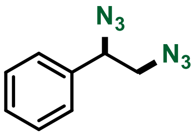

(1,2-diazidoethyl)benzene

The reaction was carried out according to **the general alkene diazidation procedure** on 0.20-mmol scale (24 h). The residue was purified by flash column chromatography (ethyl acetate/*n*-pentane) to afford the diazide product **(1,2-diazidoethyl)benzene** (**2z**) (26.3 mg, 70% yield) as an oil. **^{1}H NMR** (500 MHz, Chloroform-*d*) δ 7.44 – 7.32 (m, 5H), 4.67 (dd, *J* = 8.3, 4.9 Hz, 1H), 3.51 (dd, *J* = 12.8, 8.3 Hz, 1H), 3.45 (dd, *J* = 12.7, 4.9 Hz, 1H). **^{13}C NMR** (101 MHz, Chloroform-*d*) δ 136.32, 129.08, 129.04, 126.93, 65.52, 55.96.

HRMS for $C_8H_9N_6^+$-N_2 (ESI) $[M]^+$: calc.: 161.0822, found: 161.0822.

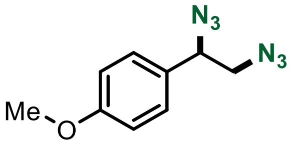

1-(1,2-diazidoethyl)-4-methoxybenzene

The reaction was carried out according to **the general alkene diazidation procedure** on 0.20-mmol scale (24 h). The residue was purified by flash column chromatography (ethyl acetate/*n*-pentane) to afford the diazide product **1-(1,2-diazidoethyl)-4-methoxybenzene** (**2a'**) (28.3 mg, 65% yield) as an oil. **^{1}H NMR** (500 MHz, Chloroform-*d*) δ 7.26 (d, *J* = 8.7 Hz, 2H), 6.93 (d, *J* = 8.7 Hz, 2H), 4.62 (dd, *J* = 8.3, 5.0 Hz, 1H), 3.82 (s, 3H), 3.49 (dd, *J* = 12.7, 8.3 Hz, 1H), 3.41 (dd, *J* = 12.7, 5.1 Hz, 1H). **^{13}C NMR** (101 MHz, Chloroform-*d*) δ 128.27, 127.55, 114.45, 113.68, 65.04, 55.89, 55.33.

HRMS for $C_9H_{11}N_6O^+$ (ESI) $[M]^+$: calc.: 191.0927, found: 191.0927.

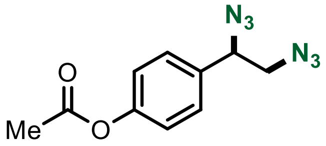

4-(1,2-diazidoethyl)phenyl acetate

The reaction was carried out according to **the general alkene diazidation procedure**

on 0.20-mmol scale (24 h). The residue was purified by flash column chromatography (ethyl acetate/*n*-pentane) to afford the diazide product **4-(1,2-diazidoethyl)phenyl acetate** (**2b'**) (36.9 mg, 75% yield) as an oil. **^{1}H NMR** (500 MHz, Chloroform-*d*) δ 7.35 (d, *J* = 8.5 Hz, 2H), 7.15 (d, *J* = 8.6 Hz, 2H), 4.67 (dd, *J* = 8.4, 4.8 Hz, 1H), 3.49 (dd, *J* = 12.8, 8.3 Hz, 1H), 3.43 (dd, *J* = 12.8, 4.8 Hz, 1H), 2.31 (s, 3H). **^{13}C NMR** (101 MHz, Chloroform-*d*) δ 169.18, 151.01, 133.92, 128.03, 122.25, 64.99, 56.03, 21.10.

HRMS for $C_{10}H_{11}N_6O_2^+$ (ESI) $[M]^+$: calc.: 219.0877, found: 219.0872.

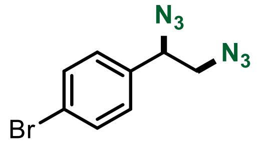

1-bromo-4-(1,2-diazidoethyl)benzene

The reaction was carried out according to **the general alkene diazidation procedure** on 0.20-mmol scale (24 h). The residue was purified by flash column chromatography (ethyl acetate/*n*-pentane) to afford the diazide product **1-bromo-4-(1,2-diazidoethyl)benzene** (**2c'**) (37.9 mg, 71% yield) as an oil. **^{1}H NMR** (500 MHz, Chloroform-*d*) δ 7.55 (d, *J* = 8.4 Hz, 2H), 7.22 (d, *J* = 8.4 Hz, 2H), 4.63 (dd, *J* = 8.0, 5.0 Hz, 1H), 3.48 (dd, *J* = 12.7, 8.0 Hz, 1H), 3.43 (dd, *J* = 12.8, 5.0 Hz, 1H). **^{13}C NMR** (101 MHz, Chloroform-*d*) δ 135.39, 132.25, 128.58, 123.08, 64.83, 55.80.

HRMS for $C_8H_8BrN_6^+$-N_2 (ESI) $[M]^+$: calc.: 238.9927, found: 238.9923.

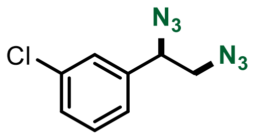

1-chloro-3-(1,2-diazidoethyl)benzene

The reaction was carried out according to **the general alkene diazidation procedure** on 0.20-mmol scale (24 h). The residue was purified by flash column chromatography (ethyl acetate/*n*-pentane) to afford the diazide product **1-chloro-3-(1,2-diazidoethyl)benzene** (**2d'**) (30.6 mg, 69% yield) as an oil. **^{1}H NMR** (500 MHz, Chloroform-*d*) δ 7.37 – 7.33 (m, 3H), 7.24 – 7.21 (m, 1H), 4.64 (dd, *J* = 8.0, 5.0 Hz,

1H), 3.49 (dd, J = 12.8, 8.0 Hz, 1H), 3.44 (dd, J = 12.8, 5.0 Hz, 1H). **^{13}C NMR** (101 MHz, Chloroform-*d*) δ 138.42, 135.04, 130.35, 129.21, 127.13, 125.07, 64.88, 55.89.
HRMS for $C_8H_8ClN_6^+$-N_2 (ESI) $[M]^+$: calc.: 195.0432, found: 195.0434.

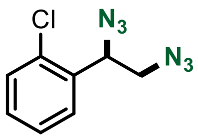

1-chloro-2-(1,2-diazidoethyl)benzene

The reaction was carried out according to **the general alkene diazidation procedure** on 0.20-mmol scale (24 h). The residue was purified by flash column chromatography (ethyl acetate/*n*-pentane) to afford the diazide product **1-chloro-2-(1,2-diazidoethyl)benzene** (**2e'**) (25.8 mg, 58% yield) as an oil. **^{1}H NMR** (500 MHz, Chloroform-*d*) δ 7.50 (dd, J = 7.6, 1.8 Hz, 1H), 7.42 (dd, J = 7.7, 1.5 Hz, 1H), 7.38 – 7.29 (m, 2H), 5.22 (dd, J = 8.3, 3.8 Hz, 1H), 3.55 (dd, J = 12.8, 3.8 Hz, 1H), 3.41 (dd, J = 12.8, 8.3 Hz, 1H). **^{13}C NMR** (101 MHz, Chloroform-*d*) δ 134.10, 132.64, 129.95, 128.08, 127.55, 62.10, 54.83.
HRMS for $C_8H_8ClN_6^+$-N_2 (ESI) $[M]^+$: calc.: 195.0432, found: 195.0435.

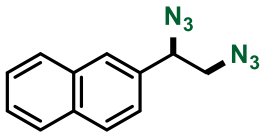

2-(1,2-diazidoethyl)naphthalene

The reaction was carried out according to **the general alkene diazidation procedure** on 0.20-mmol scale (24 h). The residue was purified by flash column chromatography (ethyl acetate/*n*-pentane) to afford the diazide product **2-(1,2-diazidoethyl)naphthalene** (**2f'**) (33.3 mg, 70% yield) as an oil. **^{1}H NMR** (500 MHz, Chloroform-*d*) δ 7.92 – 7.85 (m, 3H), 7.82 (d, J = 1.7 Hz, 1H), 7.57 – 7.52 (m, 2H), 7.43 (dd, J = 8.5, 1.9 Hz, 1H), 4.84 (dd, J = 8.3, 4.9 Hz, 1H), 3.61 (dd, J = 12.8, 8.3 Hz, 1H), 3.53 (dd, J = 12.8, 4.9 Hz, 1H). **^{13}C NMR** (101 MHz, Chloroform-*d*) δ 133.65, 133.42, 133.14, 129.13, 128.04, 127.78, 126.72, 126.55, 123.97, 65.72, 55.90.
HRMS for $C_{12}H_{11}N_6^+$-N_2 (ESI) $[M]^+$: calc.: 211.0978, found: 211.0979.

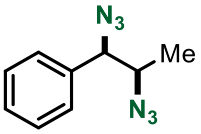

1,2-diazidopropyl)benzene

The reaction was carried out according to **the general alkene diazidation procedure** on 0.20-mmol scale (24 h). The residue was purified by flash column chromatography (ethyl acetate/*n*-pentane) to afford the diazide product **1,2-diazidopropyl)benzene (2g')** (28.7 mg, 71% yield, d.r. = 1.6:1) as an oil. **^{1}H NMR** (500 MHz, Chloroform-*d*) δ 7.45 – 7.30 (m, 5H), 4.52 (d, *J* = 5.8 Hz, 0.62H), 4.37 (d, *J* = 7.7 Hz, 0.39H), 3.75 – 3.63 (m, 1H), 1.26 (d, *J* = 6.6 Hz, 2.36H), 1.11 (d, *J* = 6.7 Hz, 1.21H). **^{13}C NMR** (101 MHz, Chloroform-*d*) δ 136.52, 136.29, 129.23, 129.09, 129.04, 127.80, 71.03, 69.89, 61.76, 61.28, 16.99, 15.29.

HRMS for $C_9H_{11}N_6^+$-N_2 (ESI) [M]$^+$: calc.: 175.0978, found: 175.0979.

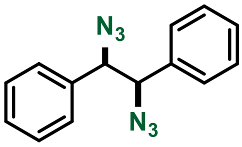

1,2-diazido-1,2-diphenylethane

The reaction was carried out according to **the general alkene diazidation procedure** on 0.20-mmol scale (24 h). The residue was purified by flash column chromatography (ethyl acetate/*n*-pentane) to afford the diazide product **1,2-diazido-1,2-diphenylethane (2h')** (33.8 mg, 64% yield, d.r. = 2.2:1) as an oil. **^{1}H NMR** (500 MHz, Chloroform-*d*) δ 7.30 (dd, *J* = 5.4, 1.9 Hz, 2H), 7.21 – 7.12 (m, 5.46H), 6.99 (dd, *J* = 6.6, 3.0 Hz, 2.71H), 4.61 (s, 0.62H), 4.56 (s, 1.37H). **^{13}C NMR** (101 MHz, Chloroform-*d*) δ 135.81, 135.75, 128.92, 128.66, 128.63, 128.52, 127.92, 127.63, 70.71, 69.65.

HRMS for $C_{14}H_{13}N_6^+$-N_2 (ESI) [M]$^+$: calc.: 237.1135, found: 237.1139.

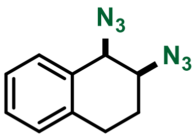

1,2-diazido-1,2,3,4-tetrahydronaphthalene

The reaction was carried out according to **the general alkene diazidation procedure** on 0.20-mmol scale (24 h). The residue was purified by flash column chromatography (ethyl acetate/*n*-pentane) to afford the diazide product **1,2-diazido-1,2,3,4-tetrahydronaphthalene** (**2i'**) (32.1 mg, 75% yield, d.r. = 4.9:1) as an oil. **^{1}H NMR** (500 MHz, Chloroform-*d*) δ 7.30 (dd, *J* = 5.6, 3.6 Hz, 1H), 7.21 – 7.18 (m, 2H), 7.07 (dd, *J* = 5.4, 3.6 Hz, 1H), 4.59 – 4.55 (m, 0.17H), 4.36 (d, *J* = 6.6 Hz, 0.84H), 3.80 (ddd, *J* = 8.8, 6.7, 3.2 Hz, 0.87H), 3.73 (dt, *J* = 11.5, 3.4 Hz, 0.17H), 2.90 – 2.78 (m, 2H), 2.20 – 2.14 (m, 1H), 1.95 – 1.87 (m, 1H). **^{13}C NMR** (101 MHz, Chloroform-*d*) δ 135.64, 135.35, 131.69, 131.51, 129.59, 129.30, 129.00, 128.96, 128.53, 126.71, 126.57, 63.49, 62.52, 61.54, 59.73, 27.37, 25.97, 25.10, 22.83.

HRMS for $C_{10}H_{11}N_6^{+}$-N_2 (ESI) $[M]^{+}$: calc.: 187.0978, found: 187.0976.

2.3 Synthetic utilization

General Procedure for Scaling Up in Batch Reactor

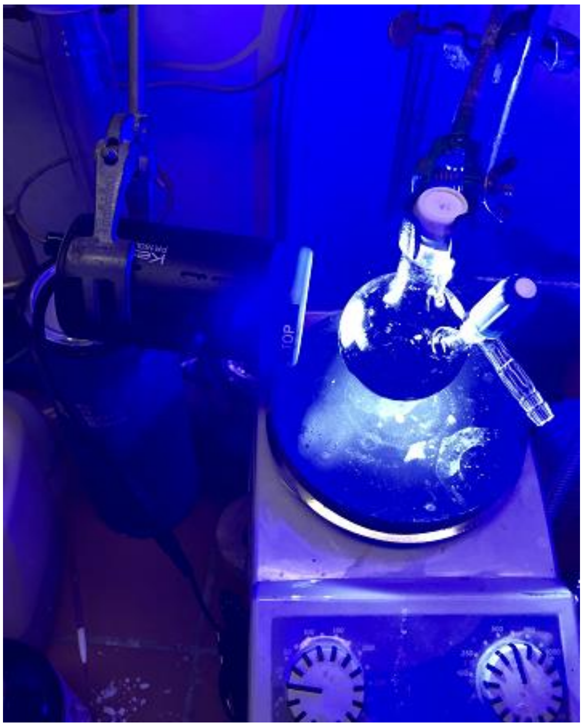

Supplementary Figure 2. Batch reactor set-up with light irradiation

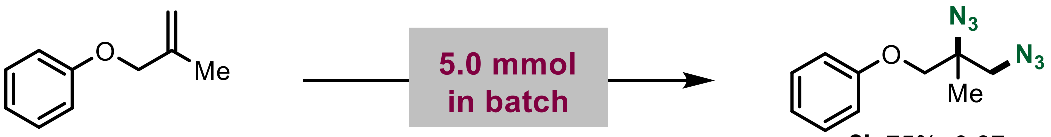

2i, 75%, 0.87 g

A 100 mL Schlenk tube equipped with a magnetic stir bar was charged with $Fe(NO_3)_3 \cdot 9H_2O$ (5.5 mmol, 2.22 g). Then, the tube was evacuated and backfilled with Ar (three times). ((2-methylallyl)oxy)benzene (0.74 g, 5.0 mmol, 1.0 equiv.) and $TMSN_3$ (22 mmol, 2.53 g) in CH_3CN (50 mL) were added by syringe under Ar atmosphere. The reaction tube was then sealed and was placed at a distance (app. 5 cm) from a 40 W blue kessil lamp (Figure S2). The reaction mixture was stirred for 36 h at room temperature. After the reaction, the resulting solution was filtered through a cotton plug and washed with EtOAc. The filtrate was removed under reduced pressure and the residue was purified by silica gel column chromatography (ethyl acetate/*n*-pentane) to afford the desired diazide **2i** (0.87 g, 75%).

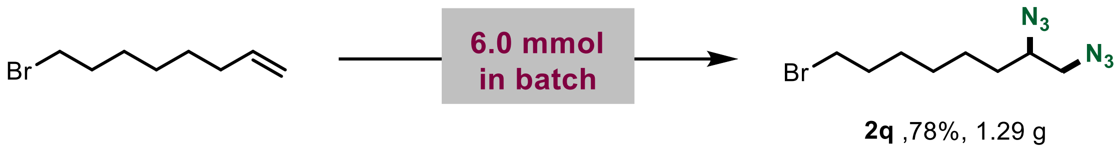

A 100 mL Schlenk tube equipped with a magnetic stir bar was charged with $Fe(NO_3)_3·9H_2O$ (6.6 mmol, 2.45 g). Then, the tube was evacuated and backfilled with Ar (three times). 8-bromooct-1-ene (1,14 g, 6.0 mmol, 1.0 equiv.) and $TMSN_3$ (25 mmol, 2.88 g) in CH_3CN (60 mL) were added by syringe under Ar atmosphere. The reaction tube was then sealed and was placed at a distance (app. 5 cm) from a 40 W blue kessil lamp (Figure S2). The reaction mixture was stirred for 36 h at room temperature. After the reaction, the resulting solution was filtered through a cotton plug and washed with EtOAc. The filtrate was removed under reduced pressure and the residue was purified by silica gel column chromatography (ethyl acetate/*n*-pentane) to afford the desired diazide **2q** (1.29 g, 75%).

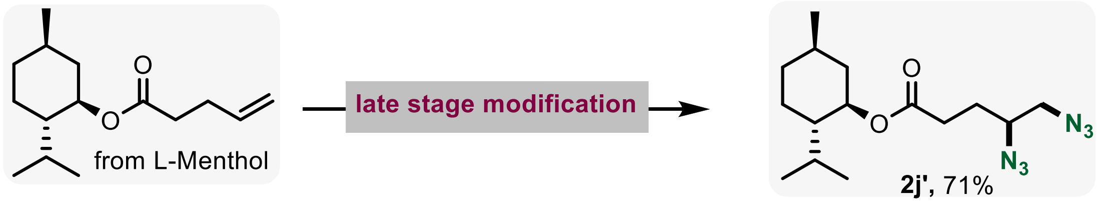

A 10 mL Schlenk tube equipped with a magnetic stir bar was charged with $Fe(NO_3)_3·9H_2O$ (0.12 mmol, 50 mg). Then, the tube was evacuated and backfilled with Ar (three times). Alkene (23.8 mg, 0.10 mmol, 1.0 equiv.) and $TMSN_3$ (0.44 mmol) in CH_3CN (2 mL) were added by syringe under Ar atmosphere. The reaction tube was then sealed and was placed at a distance (app. 5 cm) from a 40 W blue kessil lamp (Figure S1). The reaction mixture was stirred for 24 h at room temperature. After the reaction, the resulting solution was filtered through a cotton plug and washed with EtOAc. The filtrate was removed under reduced pressure and the residue was purified by silica gel column chromatography (ethyl acetate/*n*-pentane) to afford the desired diazide **(1*R*,2*S*,5*R*)-2-isopropyl-5-methylcyclohexyl-4,5-diazidopentanoate 2j'** (22.9 mg, 71% yield, d.r. = 1:1). ^{1}H NMR (500 MHz,

Chloroform-*d*) δ 4.74 – 4.66 (m, 1H), 3.60 – 3.53 (m, 1H), 3.44 (dd, *J* = 12.7, 4.1 Hz, 1H), 3.35 (dd, *J* = 12.8, 7.5 Hz, 1H), 2.48 – 2.41 (m, 2H), 2.00 – 1.95 (m, 1H), 1.94 – 1.87 (m, 1H), 1.85 – 1.81 (m, 1H), 1.79 – 1.72 (m, 1H), 1.70 – 1.66 (m, 2H), 1.52 – 1.45 (m, 1H), 1.41 – 1.33 (m, 1H), 1.10 – 1.03 (m, 1H), 1.01 – 0.94 (m, 1H), 0.92 – 0.88 (m, 7H), 0.76 (dd, *J* = 6.9, 1.9 Hz, 3H). ^{13}C NMR (101 MHz, Chloroform-*d*) δ 171.96, 74.68, 74.67, 61.23, 61.21, 54.88, 47.03, 46.99, 40.92, 40.89, 34.20, 31.38, 30.66, 30. 65, 27.15, 27.12, 26.39, 26.36, 23.44, 21.98, 20.72, 20.70, 16.30, 16.29.

HRMS for $C_{15}H_{27}N_6O_2^+$-N_2 (ESI) $[M]^+$: calc.: 295.2129, found: 295.2128.

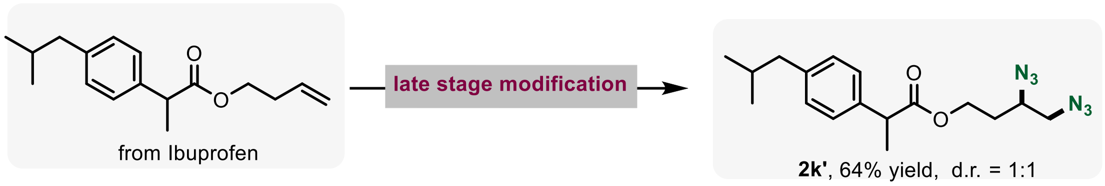

A 10 mL Schlenk tube equipped with a magnetic stir bar was charged with $Fe(NO_3)_3·9H_2O$ (0.24 mmol, 100 mg). Then, the tube was evacuated and backfilled with Ar (three times). Ibuprofen derivative (52.0 mg, 0.20 mmol, 1.0 equiv.) and $TMSN_3$ (1.0 mmol) in CH_3CN (2 mL) were added by syringe under Ar atmosphere. The reaction tube was then sealed and was placed at a distance (app. 5 cm) from a 40 W blue kessil lamp (Figure S1). The reaction mixture was stirred for 24 h at room temperature. After the reaction, the resulting solution was filtered through a cotton plug and washed with EtOAc. The filtrate was removed under reduced pressure and the residue was purified by silica gel column chromatography (ethyl acetate/*n*-pentane) to afford the desired diazide **3,4-diazidobutyl 2-(4-isobutylphenyl)propanoate 2k'** (44.1 mg, 64% yield, d.r. = 1:1). **^{1}H NMR** (500 MHz, Chloroform-*d*) δ 7.19 (d, *J* = 7.8 Hz, 2H), 7.11 (d, *J* = 7.8 Hz, 2H), 4.33 – 4.08 (m, 2H), 3.70 (q, *J* = 7.2 Hz, 1H), 3.37 – 3.12 (m, 3H), 2.45 (d, *J* = 7.2 Hz, 2H), 1.89 – 1.65 (m, 3H), 1.50 (d, *J* = 7.2 Hz, 3H), 0.90 (d, *J* = 7.6 Hz, 6H). **^{13}C NMR** (101 MHz, Chloroform-*d*) δ 174.39, 140.81, 140.77, 137.54, 137.48, 129.43, 127.07, 60.64, 60.56, 58.91, 58.88, 54.71, 54.67, 45.11, 45.07, 44.99, 30.84, 30.79, 30.15, 22.34, 18.11.

HRMS for $C_{17}H_{25}N_6O_2^+$-N_2 (ESI) $[M]^+$: calc.: 317.1972, found: 317.1974.

2.4 Mechanistic Investigations

The analysis for the reaction mixture by GC-MS

A 10 mL Schlenk tube equipped with a magnetic stir bar was charged with $Fe(NO_3)_3{\cdot}9H_2O$ (0.24 mmol, 100 mg). Then, the tube was evacuated and backfilled with Ar (three times). Alkene **1a** (29.2 mg, 0.20 mmol, 1.0 equiv.) and $TMSN_3$ (1.0 mmol) in CH_3CN (2 mL) were added by syringe under Ar atmosphere. The reaction tube was then sealed and was placed at a distance (app. 5 cm) from a 40 W blue kessil lamp (Figure S1). The reaction mixture was stirred for 24 h at room temperature. The reaction mixture was analyzed by GC-MS.

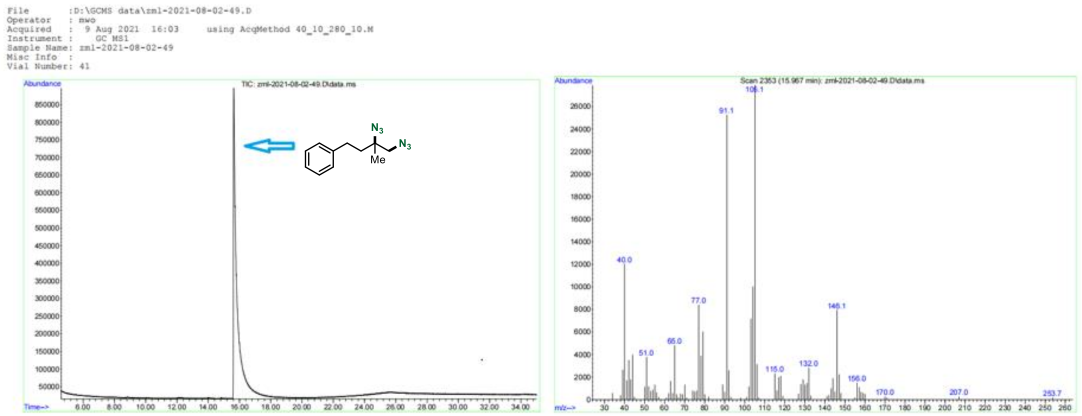

Supplementary Figure 3. Spectra of the reaction mixture by GC-MS analysis.

Control Experiment with Additives

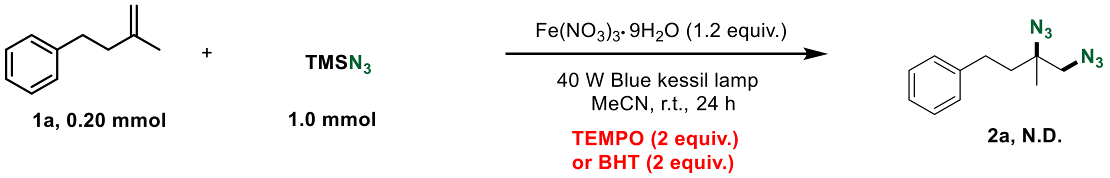

A 10 mL Schlenk tube equipped with a magnetic stir bar was charged with $Fe(NO_3)_3{\cdot}9H_2O$ (0.24 mmol, 100 mg) and TEMPO or BHT (0.4 mmol, 2 equiv.). Then, the tube was evacuated and backfilled with Ar (three times). Alkene **1a** (29.2 mg, 0.20 mmol, 1.0 equiv.) and $TMSN_3$ (1.0 mmol) in CH_3CN (2 mL) were added by syringe under Ar atmosphere. The reaction tube was then sealed and was placed at a distance (app. 5 cm) from a 40 W blue kessil lamp (Figure S1). The reaction mixture was stirred for 24 h at room temperature. The corresponding diazide product **2a** was

not detected according to both TLC and GC-Mass analysis. The TEMPO-adduct **3** was detected by ESI-HRMS.

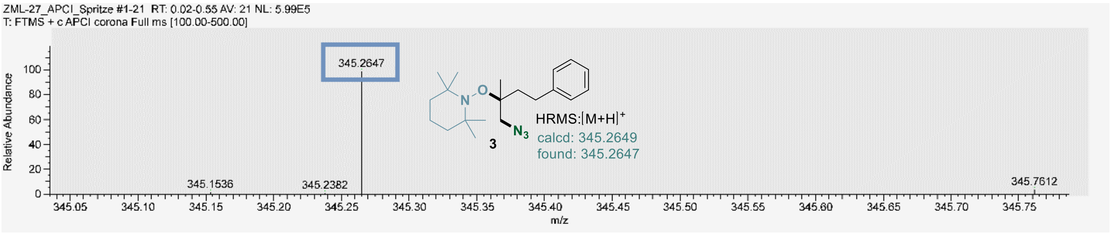

Supplementary Figure 4. ESI-HRMS Spectra using TEMPO

Radical cyclic experiments

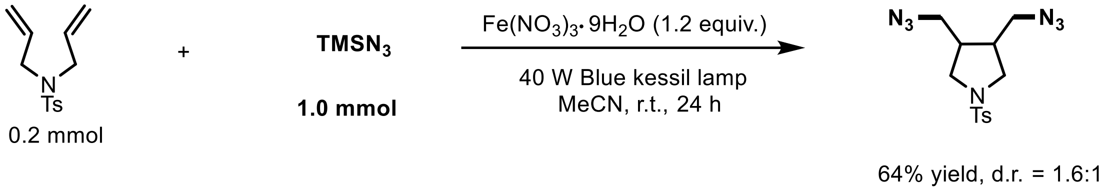

A 10 mL Schlenk tube equipped with a magnetic stir bar was charged with $Fe(NO_3)_3{\cdot}9H_2O$ (0.24 mmol, 100 mg). Then, the tube was evacuated and backfilled with Ar (three times). *N*,*N*-diallyl-4-methylbenzenesulfonamide (50.2 mg, 0.20 mmol, 1.0 equiv.) and $TMSN_3$ (1.0 mmol) in CH_3CN (2 mL) were added by syringe under Ar atmosphere. The reaction tube was then sealed and was placed at a distance (app. 5 cm) from a 40 W blue kessil lamp (Figure S1). The reaction mixture was stirred for 24 h at room temperature. After the reaction, the resulting solution was filtered through a cotton plug and washed with EtOAc. The filtrate was removed under reduced pressure and the residue was purified by silica gel column chromatography (ethyl acetate/*n*-pentane) to afford the product **3,4-bis(azidomethyl)-1-tosylpyrrolidine** (42.9 mg, 64% yield) as an oil. ^{1}H NMR (500 MHz, Chloroform-*d*) δ 7.75 – 7.70 (m, 2H), 7.36 (d, *J* = 8.0 Hz, 2H), 3.45 – 3.36 (m, 2H), 3.34 – 3.26 (m, 2H), 3.26 – 3.21 (m, 0.62H), 3.18 – 3.09 (m, 2.55H), 3.02 (dd, *J* = 10.4, 6.1 Hz, 0.58H), 2.45 – 2.43 (m, 3H), 2.41 – 2.36 (m, 1.33H), 2.13 – 2.08 (m, 0.55H). ^{13}C NMR (101 MHz, Chloroform-*d*) δ 143.95, 143.88, 133.38, 132.69, 129.79, 129.65, 127.71, 127.49, 52.83, 50.59, 50.27, 49.61, 41.22, 39.82, 21.56, 21.53.

Exclusion of radical-polar crossover pathway

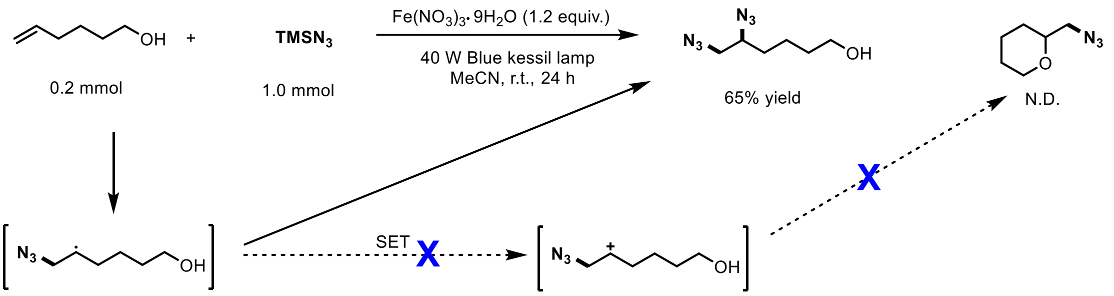

A 10 mL Schlenk tube equipped with a magnetic stir bar was charged with $Fe(NO_3)_3 \cdot 9H_2O$ (0.24 mmol, 100 mg). Then, the tube was evacuated and backfilled with Ar (three times). hex-5-en-1-ol (20.0 mg, 0.20 mmol, 1.0 equiv.) and $TMSN_3$ (1.0 mmol) in CH_3CN (2 mL) were added by syringe under Ar atmosphere. The reaction tube was then sealed and was placed at a distance (app. 5 cm) from a 40 W blue kessil lamp (Figure S1). The reaction mixture was stirred for 24 h at room temperature. After the reaction, the resulting solution was filtered through a cotton plug and washed with EtOAc. The filtrate was removed under reduced pressure and the residue was purified by silica gel column chromatography (ethyl acetate/*n*-pentane) to afford the product 5,6-diazidohexan-1-ol (23.9 mg, 65% yield) as an oil. And no cyclic product was observed by MS analysis.

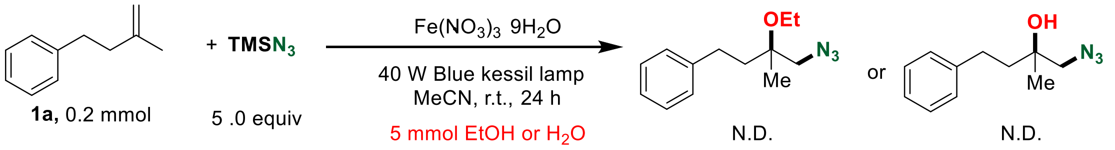

The intermolecular trapping experiments were performed with a large excess of nucleophiles such as H_2O and EtOH added into reaction system, however, the compounds trapped by nucleophiles could not be detected. A radical-polar crossover mechanism should be excluded.

3. Supplementary Notes

3.1 Copies of ^{1}H, ^{13}C and ^{19}F NMR spectra

Supplementary Figure 5. ^{1}H NMR (500 MHz, $CDCl_3$, 298K) of **(3,4-diazido-3-methylbutyl)benzene**

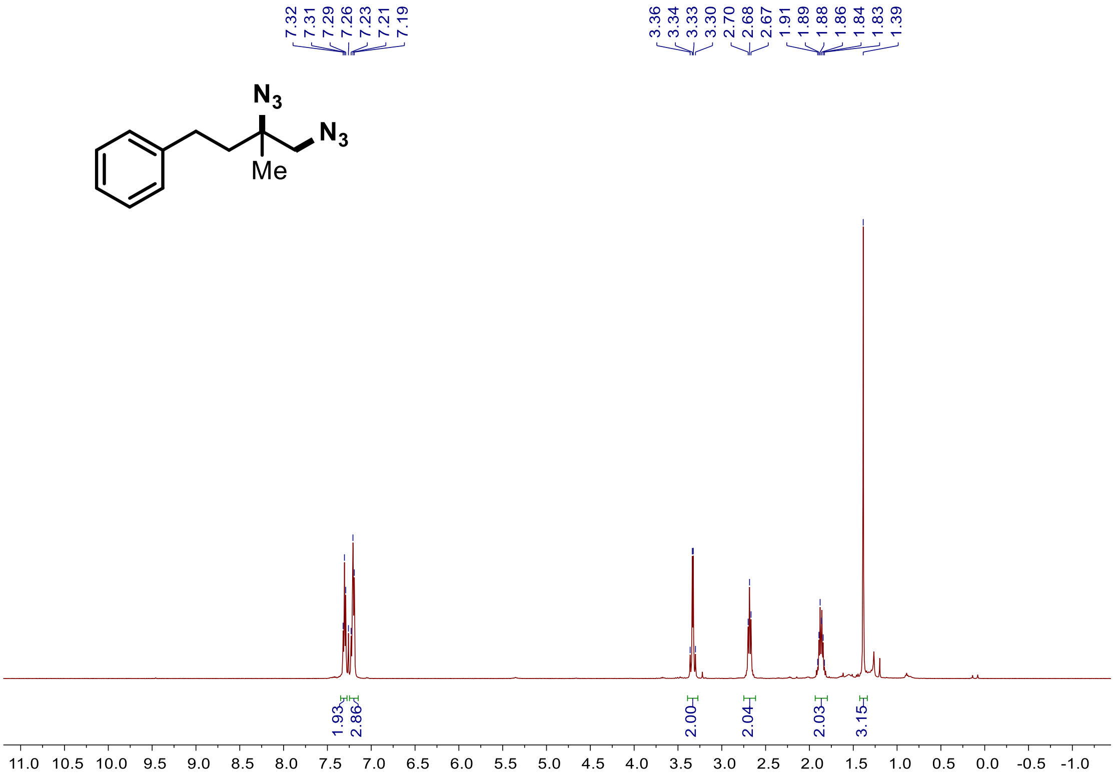

Supplementary Figure 6. ^{13}C NMR (101 MHz, $CDCl_3$, 298K) of **(3,4-diazido-3-methylbutyl)benzene**

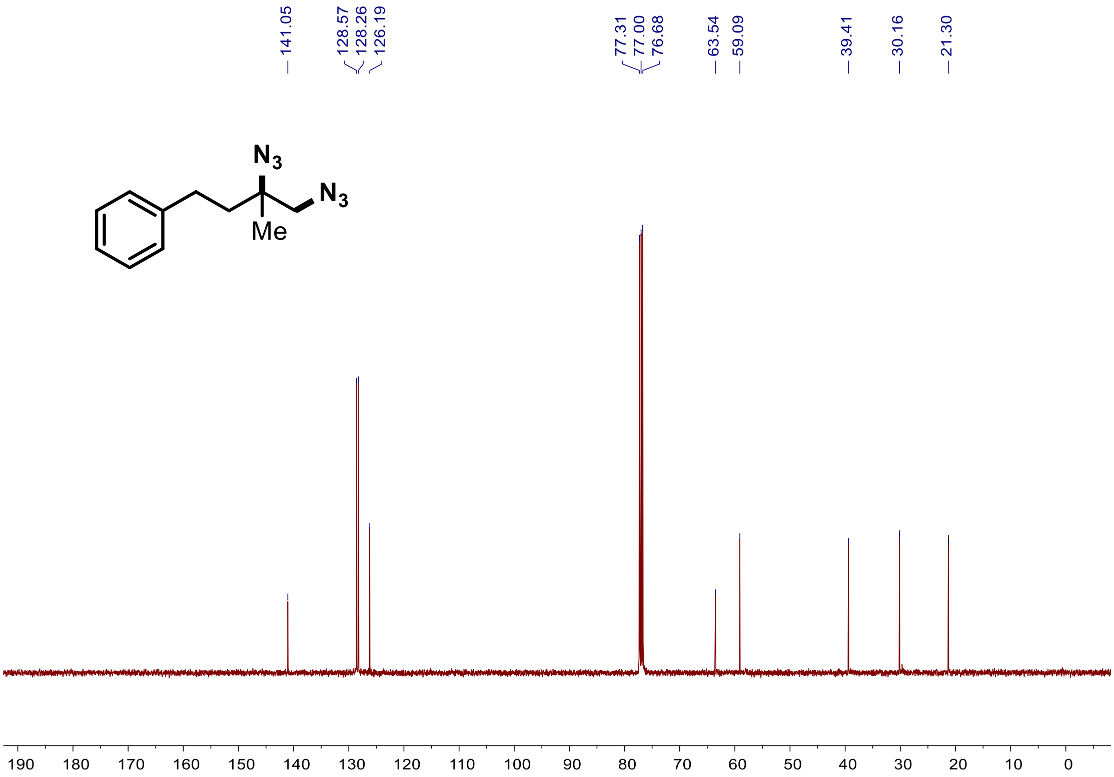

Supplementary Figure 7. ^{1}**H NMR** (500 MHz, $CDCl_3$, 298K) of **1-(3,4-diazido-3-methylbutyl)-4-methylbenzene**

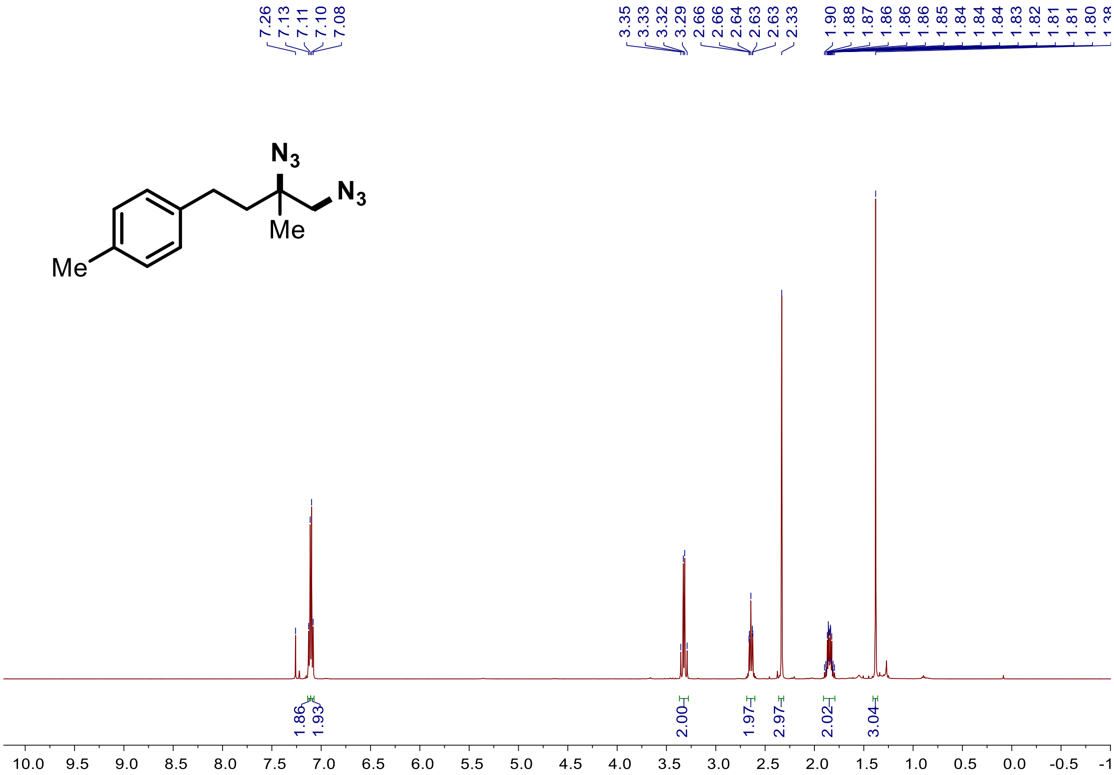

Supplementary Figure 8. ^{13}**C NMR** (101 MHz, $CDCl_3$, 298K) of **1-(3,4-diazido-3-methylbutyl)-4-methylbenzene**

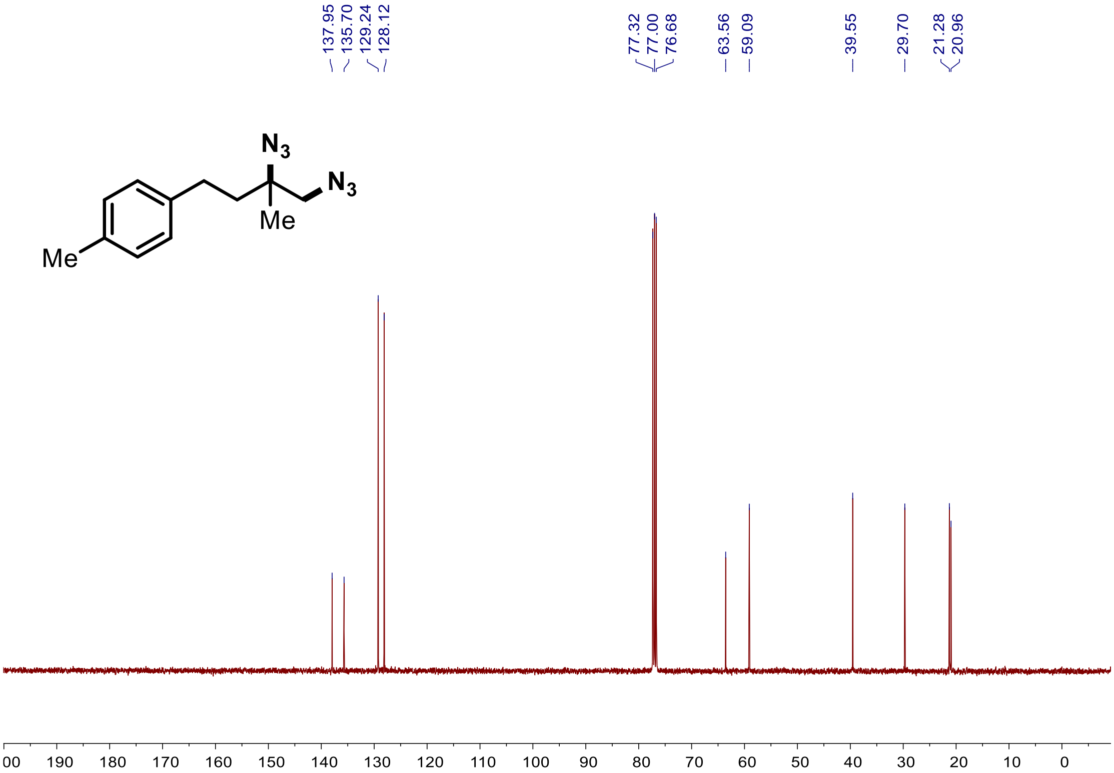

Supplementary Figure 9. **^{1}H NMR** (500 MHz, $CDCl_3$, 298K) of **1-(3,4-diazido-3-methylbutyl)-4-(trifluoromethyl)benzene**

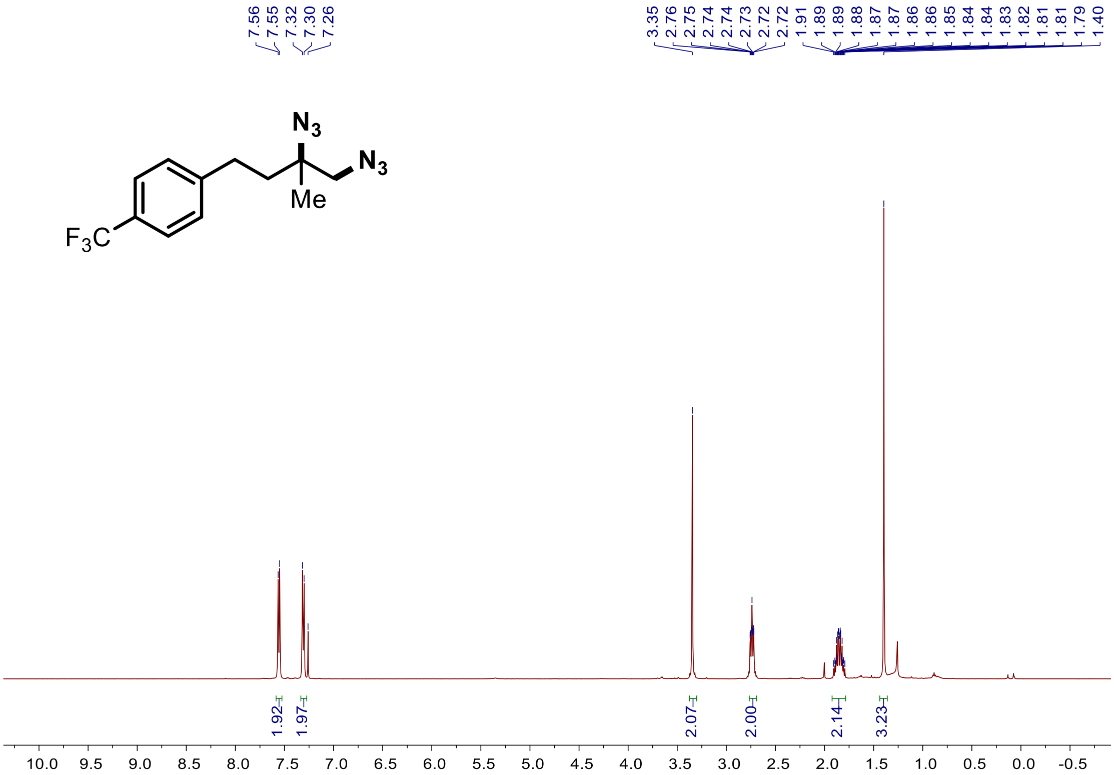

Supplementary Figure 10. **^{13}C NMR** (101 MHz, $CDCl_3$, 298K) of **1-(3,4-diazido-3-methylbutyl)-4-(trifluoromethyl)benzene**

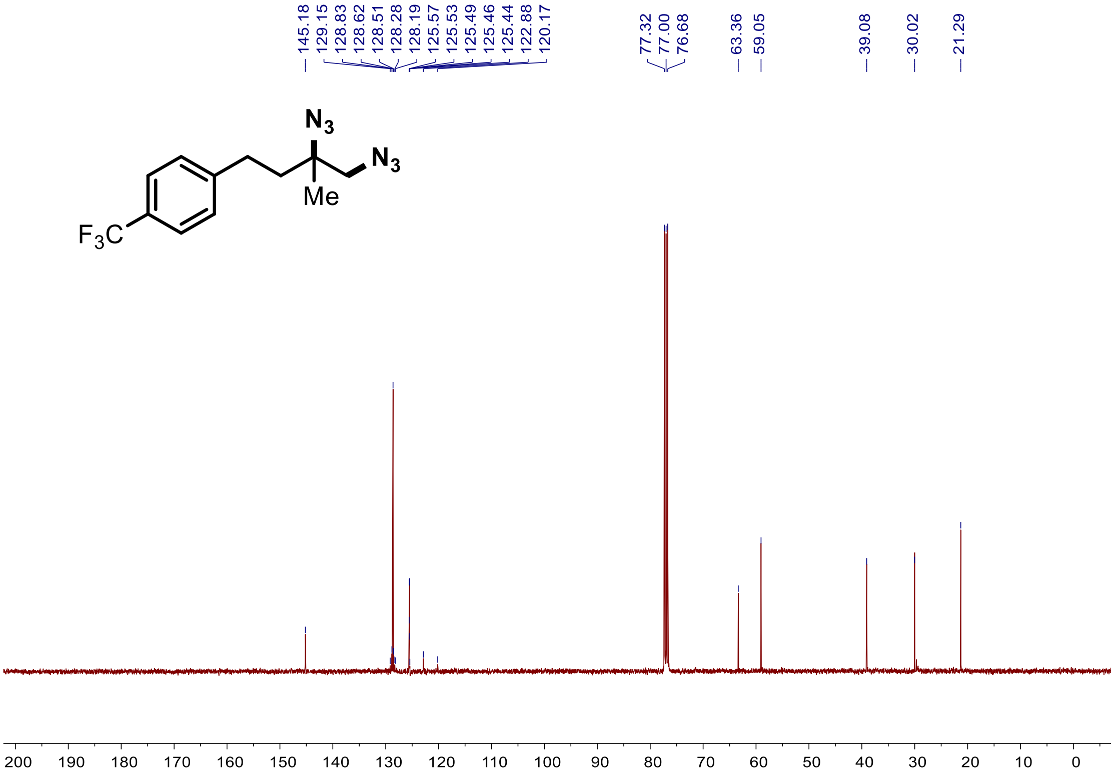

Supplementary Figure 11. ^{19}**F NMR** (471 MHz, $CDCl_3$, 298K) of **1-(3,4-diazido-3-methylbutyl)-4-(trifluoromethyl)benzene**

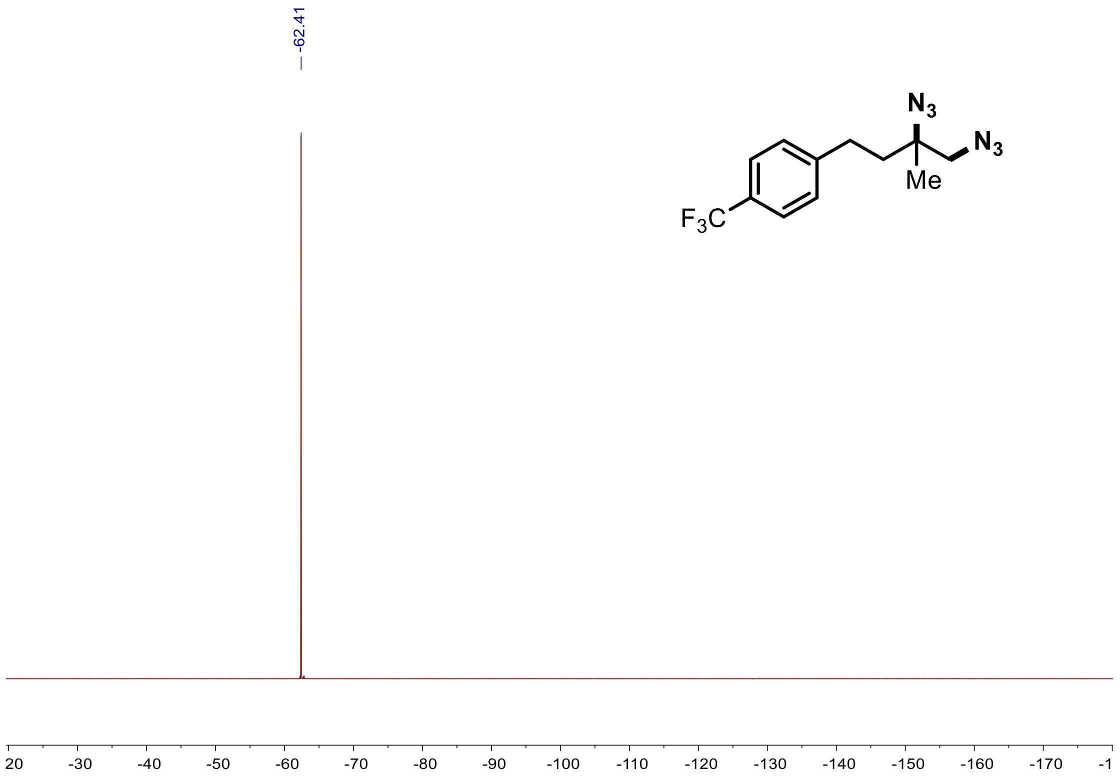

Supplementary Figure 12. ^{1}**H NMR** (500 MHz, $CDCl_3$, 298K) of **1-bromo-4-(3,4-diazido-3-methylbutyl)benzene**

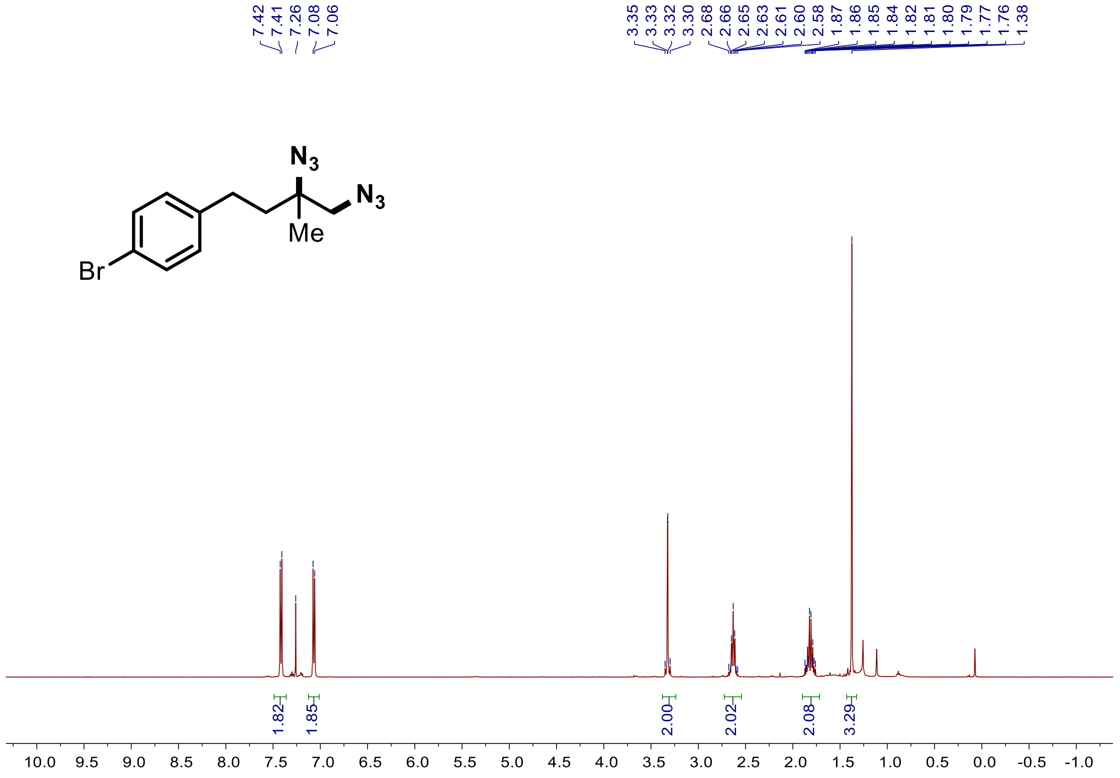

Supplementary Figure 13. ^{13}**C NMR** (101 MHz, $CDCl_3$, 298K) of **1-bromo-4-(3,4-diazido-3-methylbutyl)benzene**

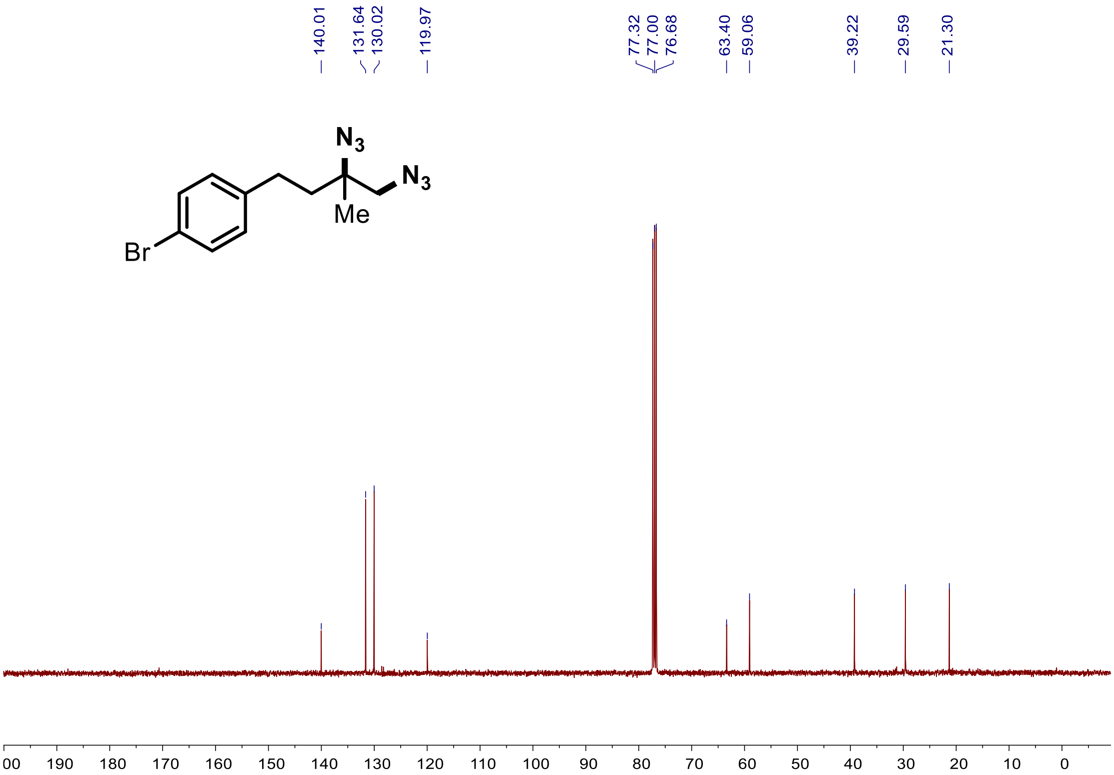

Supplementary Figure 14. ^{1}**H NMR** (500 MHz, $CDCl_3$, 298K) of **1-(3,4-diazido-3-methylbutyl)-3-methoxybenzene**

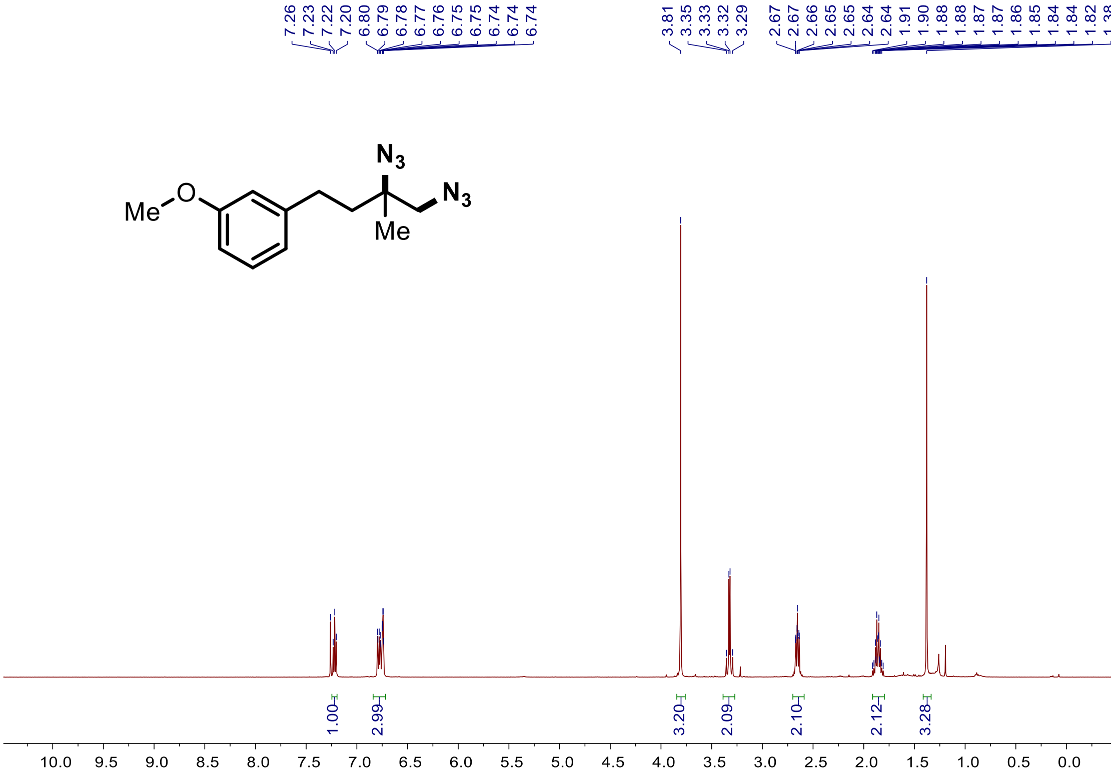

Supplementary Figure 15. ^{13}C NMR (101 MHz, $CDCl_3$, 298K) of **1-(3,4-diazido-3-methylbutyl)-3-methoxybenzene**

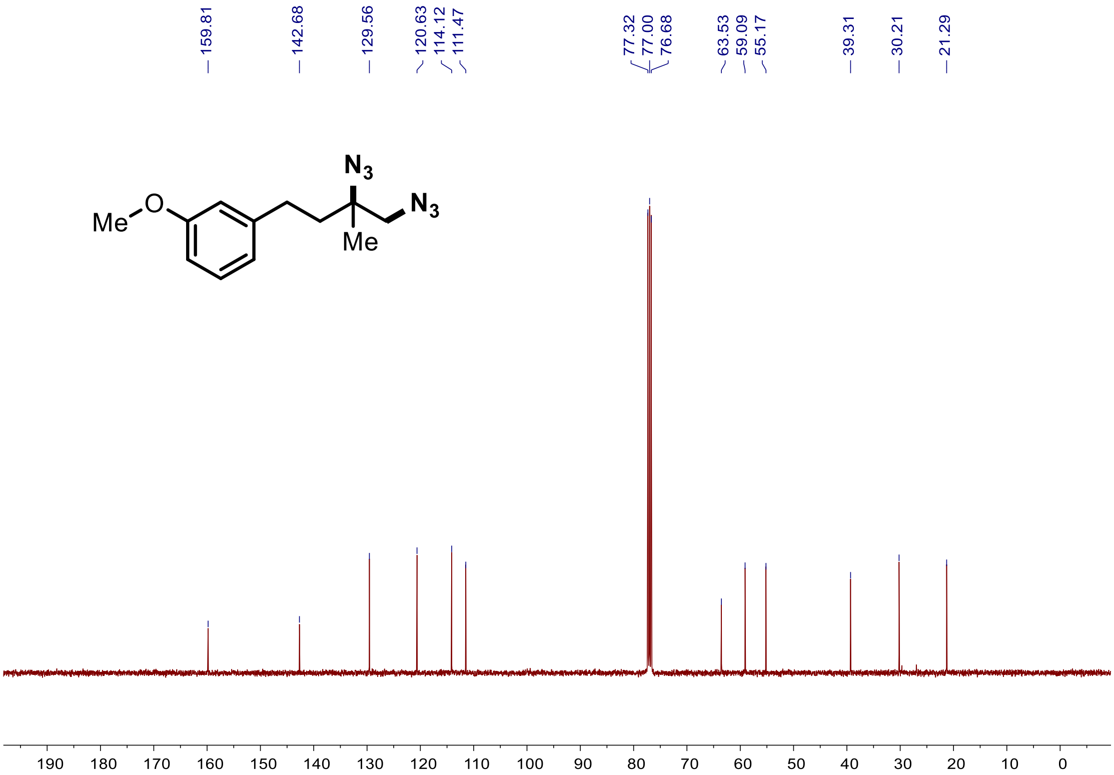

Supplementary Figure 16. ^{1}H NMR (500 MHz, $CDCl_3$, 298K) of **1-chloro-2-(3,4-diazido-3-methylbutyl)benzene**

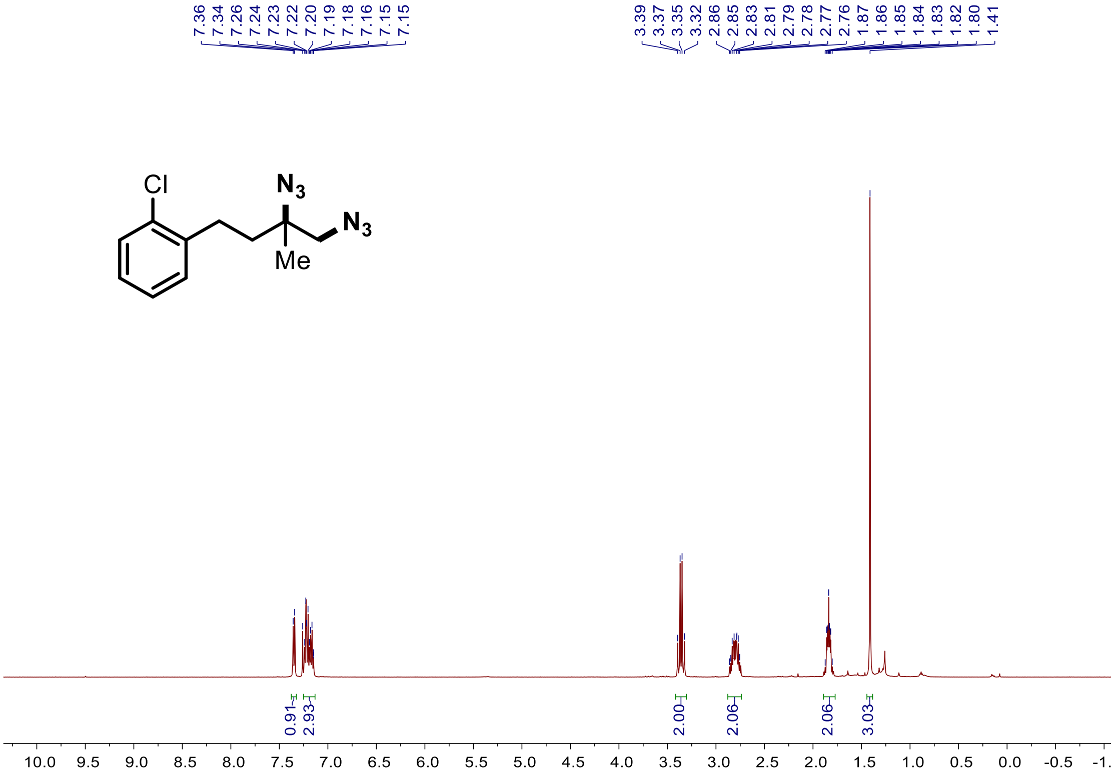

Supplementary Figure 17. ^{13}**C NMR** (101 MHz, $CDCl_3$, 298K) of **1-chloro-2-(3,4-diazido-3-methylbutyl)benzene**

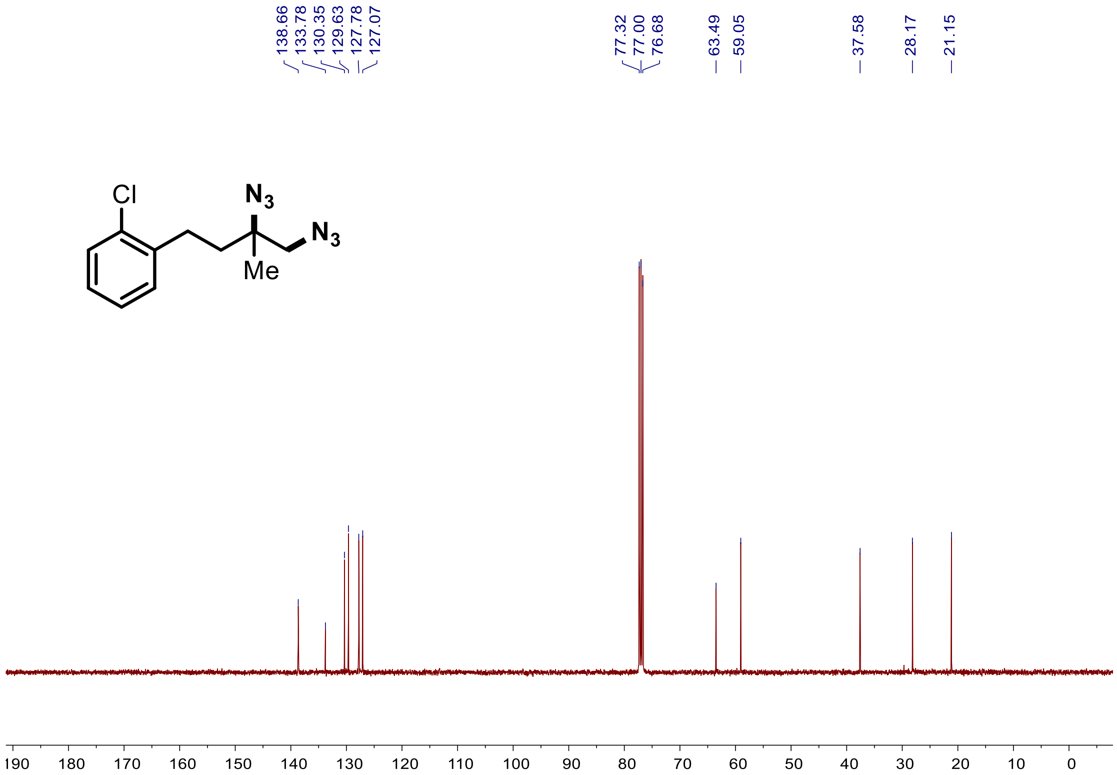

Supplementary Figure 18. ^{1}**H NMR** (500 MHz, $CDCl_3$, 298K) of **2-(3,4-diazido-3-methylbutyl)naphthalene**

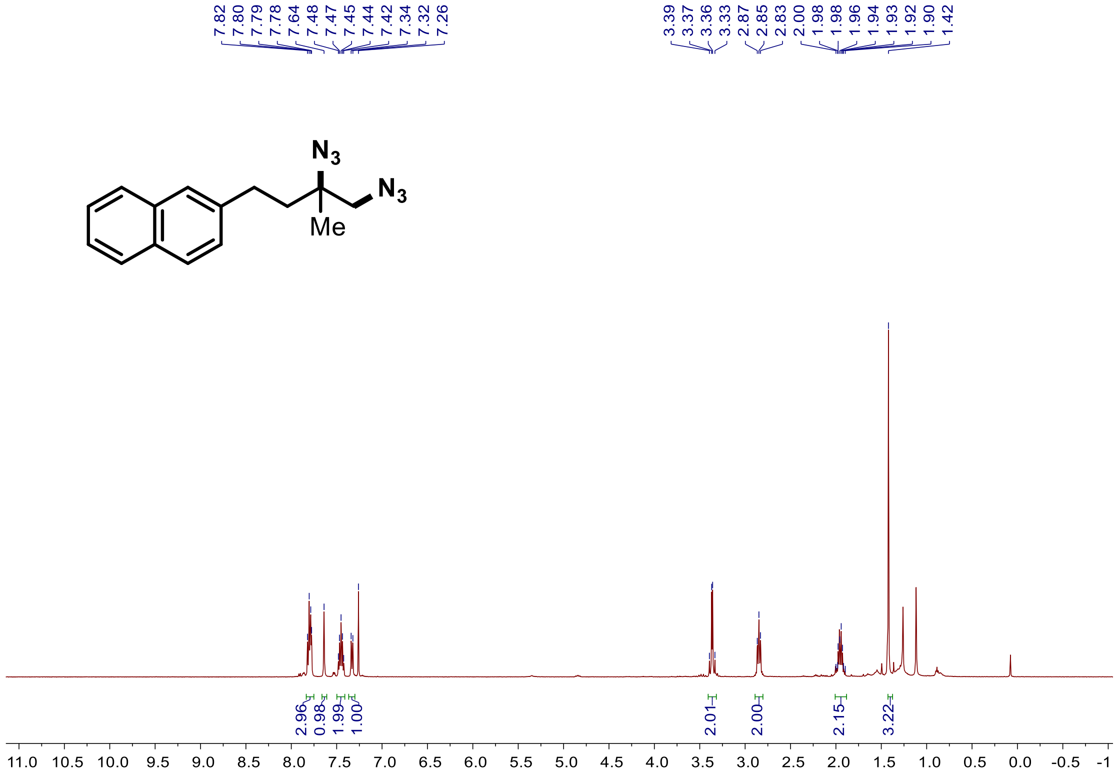

Supplementary Figure 19. ^{13}**C NMR** (101 MHz, $CDCl_3$, 298K) of **2-(3,4-diazido-3-methylbutyl)naphthalene**

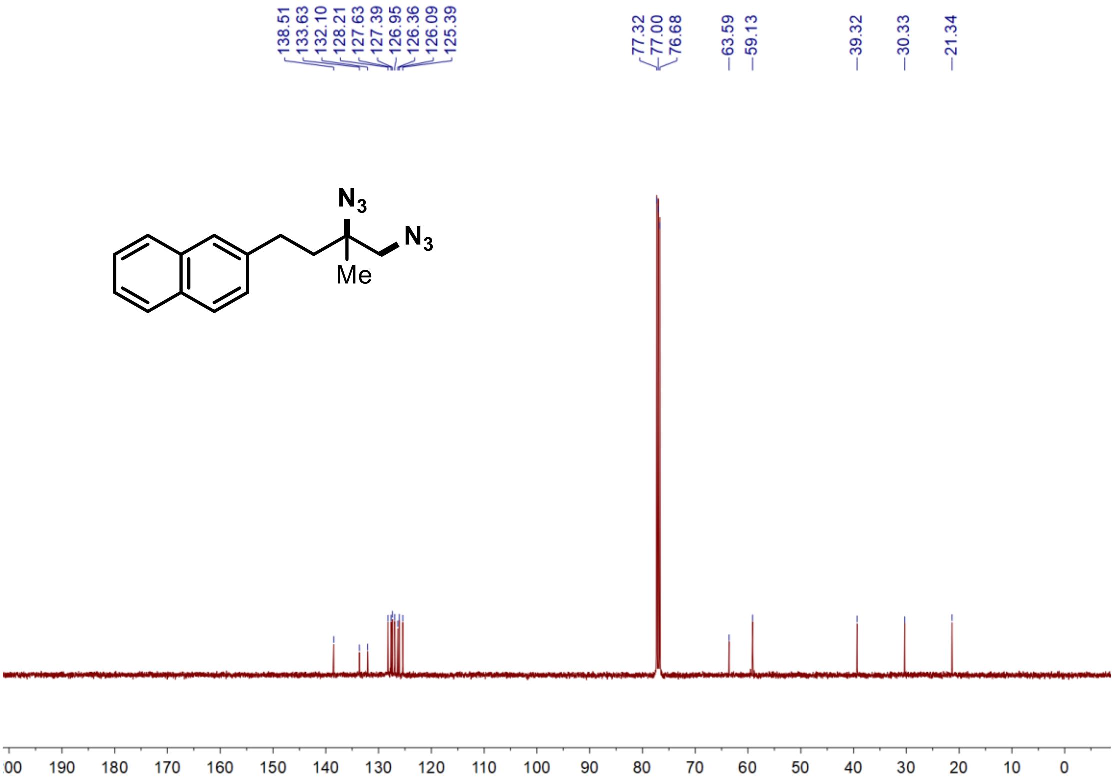

Supplementary Figure 20. ^{1}**H NMR** (500 MHz, $CDCl_3$, 298K) of **1-(3,4-diazido-3-methylbutyl)-2,3,5,6-tetrafluoro-4-methylbenzene**

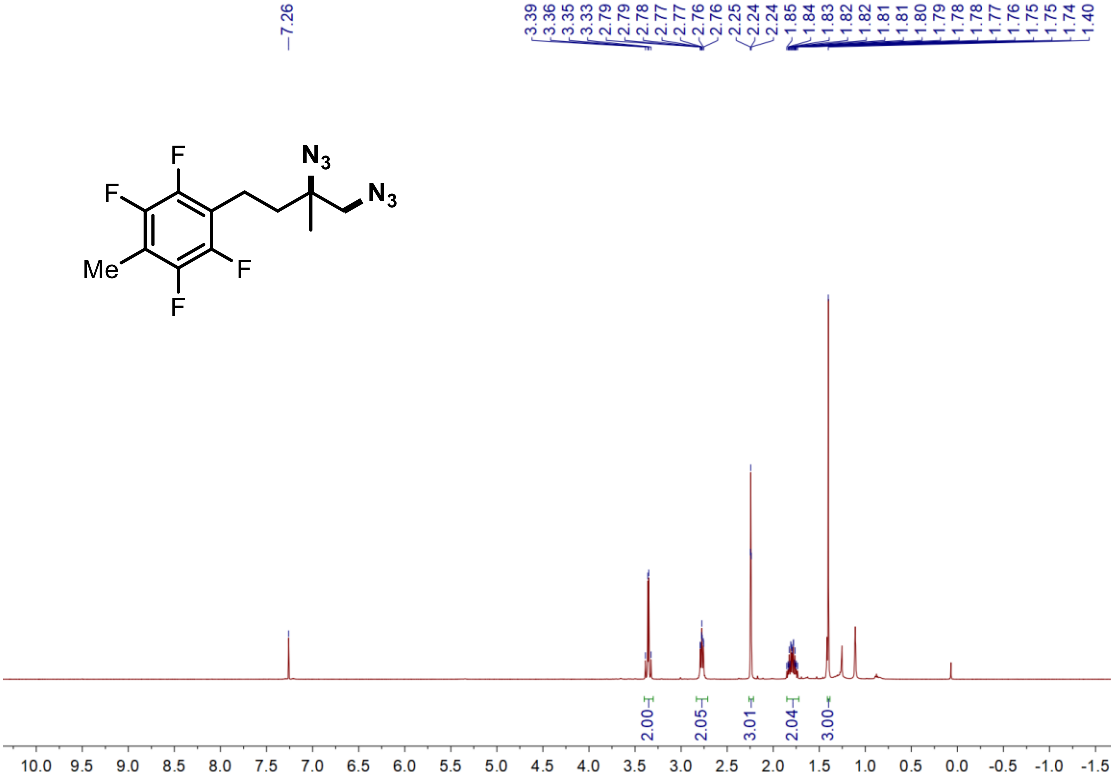

Supplementary Figure 21. ^{13}**C NMR** (101 MHz, $CDCl_3$, 298K) of **1-(3,4-diazido-3-methylbutyl)-2,3,5,6-tetrafluoro-4-methylbenzene**

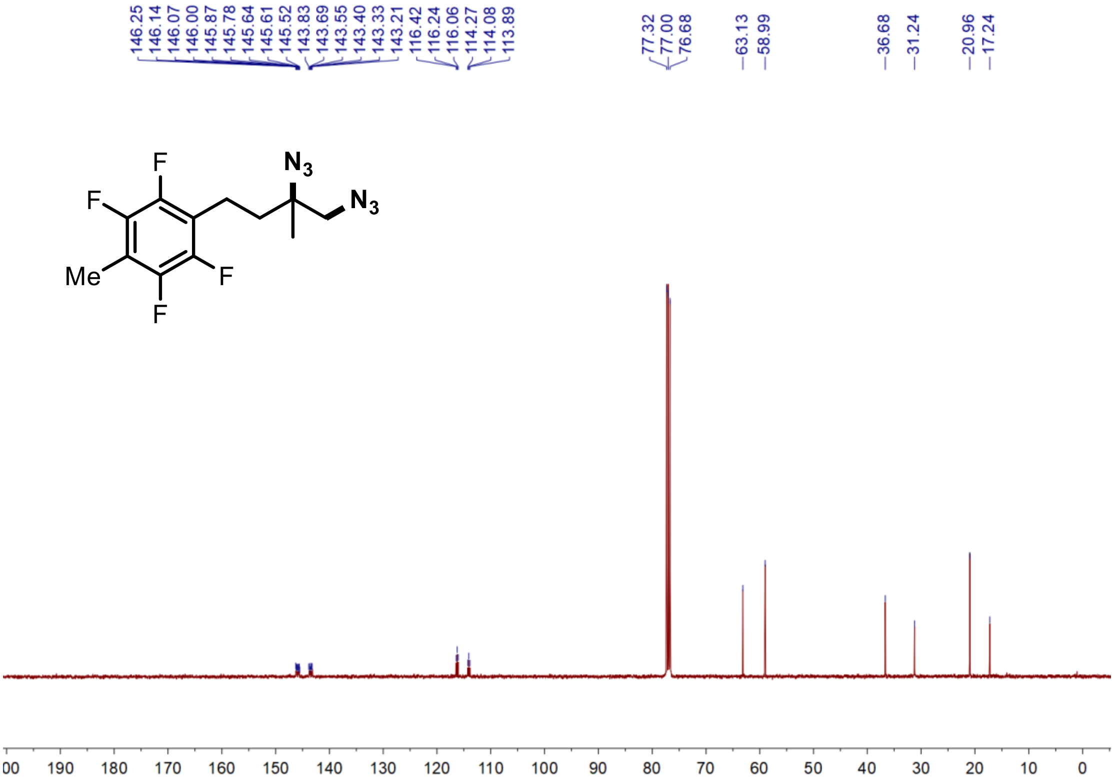

Supplementary Figure 22. ^{19}**F NMR** (471 MHz, $CDCl_3$, 298K) of **1-(3,4-diazido-3-methylbutyl)-2,3,5,6-tetrafluoro-4-methylbenzene**

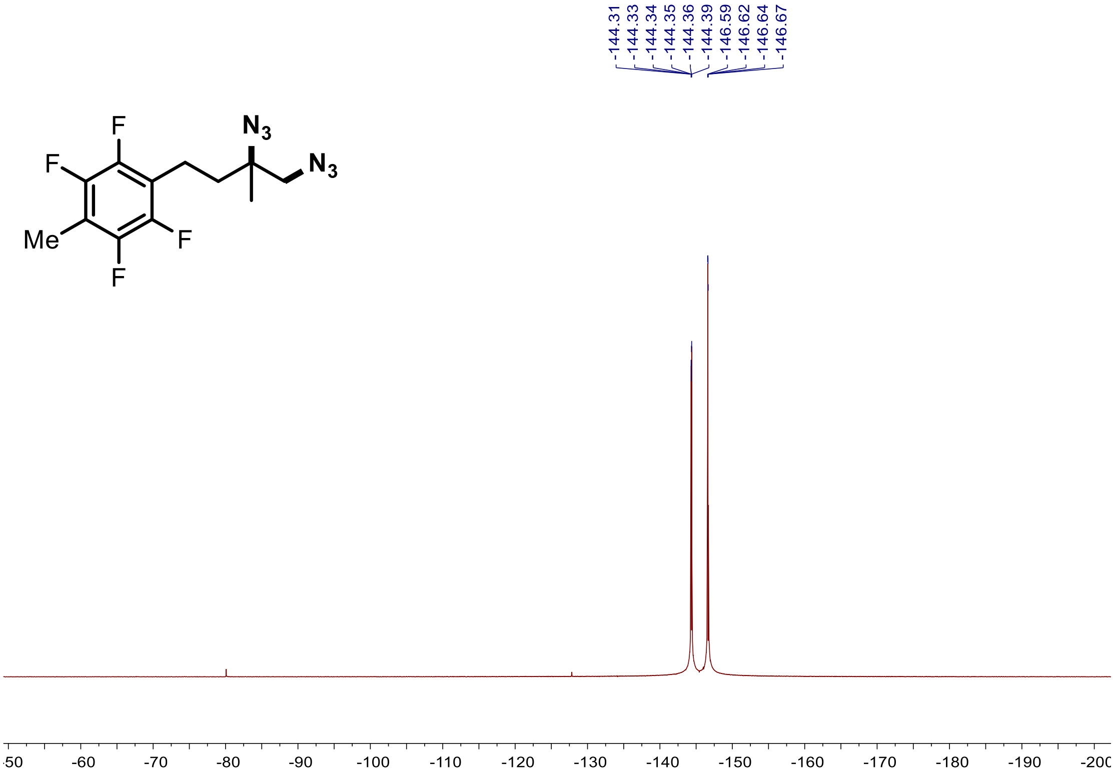

Supplementary Figure 23. ^{1}**H NMR** (500 MHz, $CDCl_3$, 298K) of **(2,3-diazido-2-methylpropoxy)benzene**

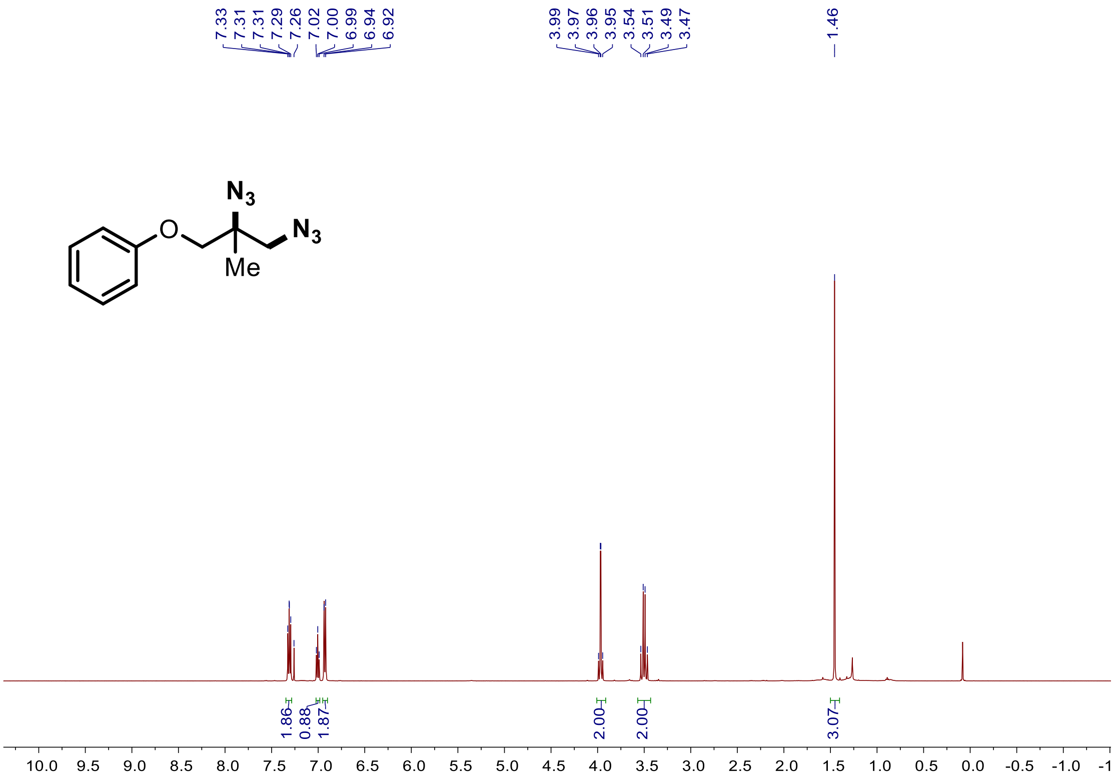

Supplementary Figure 24. ^{13}**C NMR** (101 MHz, $CDCl_3$, 298K) of **(2,3-diazido-2-methylpropoxy)benzene**

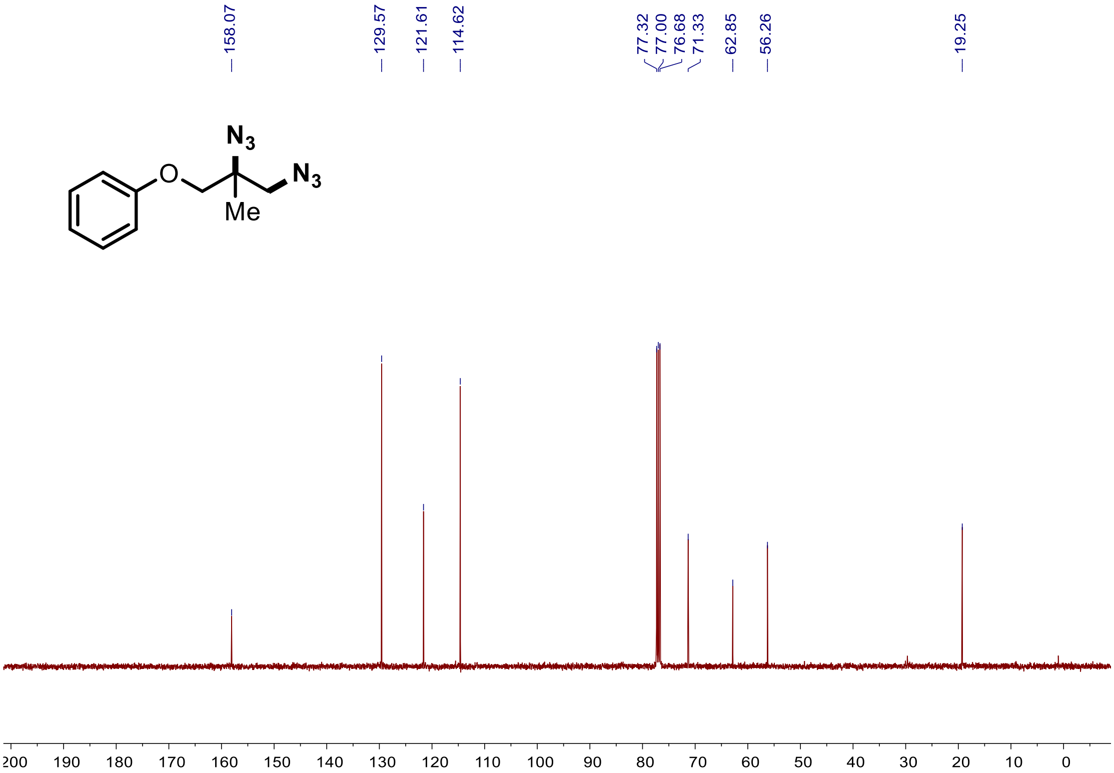

Supplementary Figure 25. ^{1}**H NMR** (500 MHz, $CDCl_3$, 298K) of **1,2-diazido-2-methylheptane**

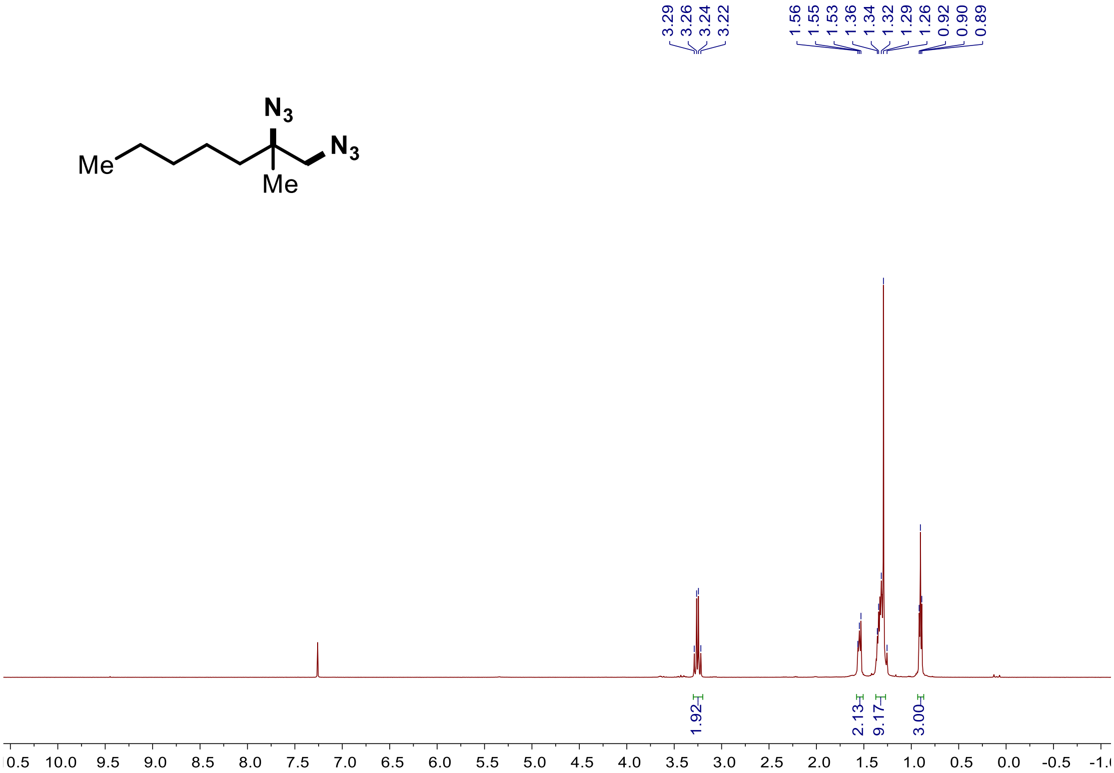

Supplementary Figure 26. ^{13}**C NMR** (101 MHz, $CDCl_3$, 298K) of **1,2-diazido-2-methylheptane**

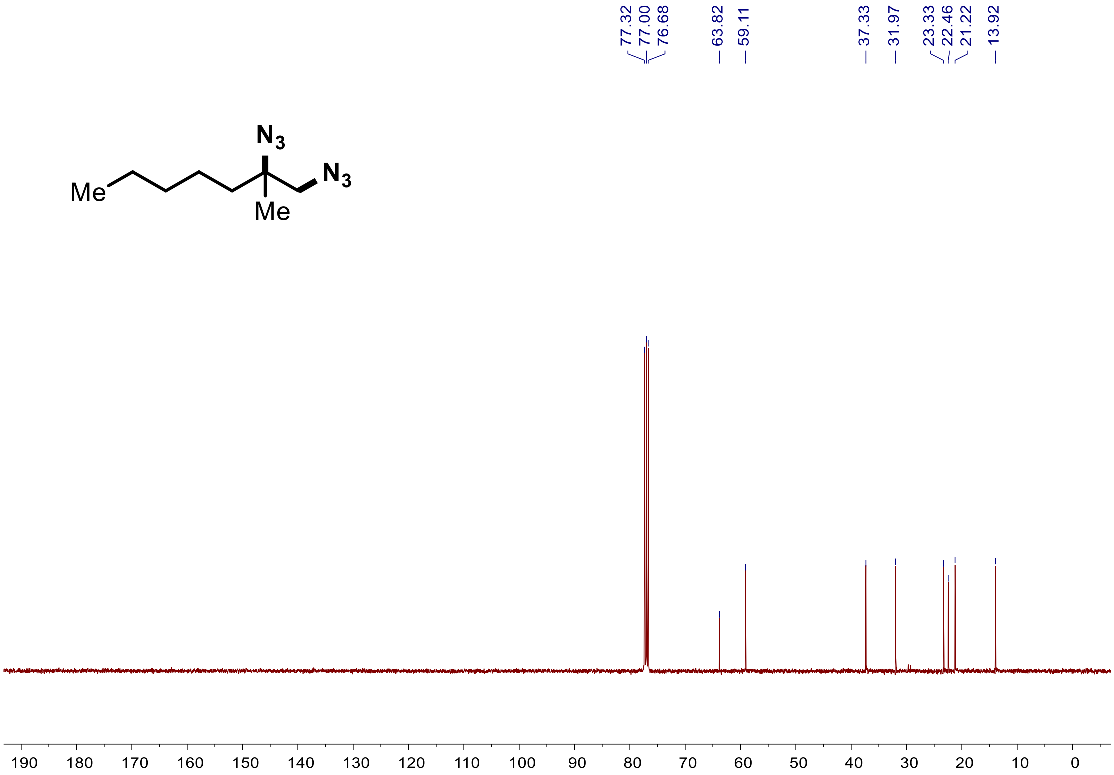

Supplementary Figure 27. ^{1}**H NMR** (500 MHz, $CDCl_3$, 298K) of **(3,4-diazidobutyl)benzene**

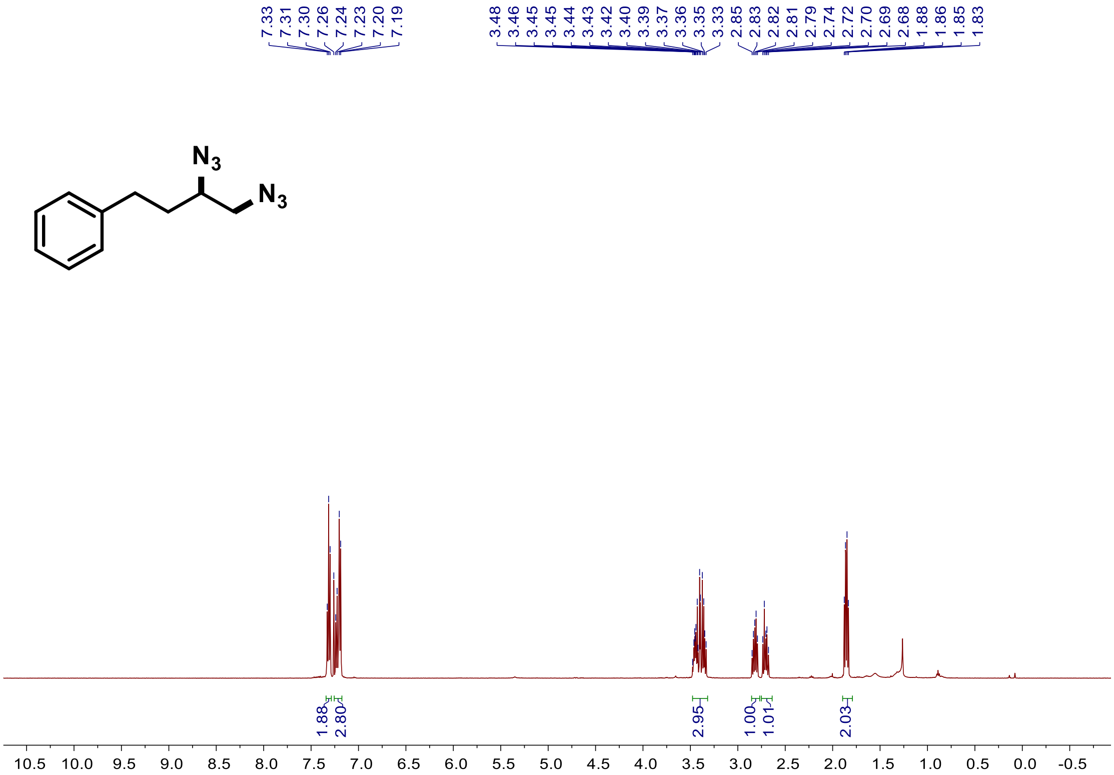

Supplementary Figure 28. ^{13}**C NMR** (101 MHz, $CDCl_3$, 298K) of **(3,4-diazidobutyl)benzene**

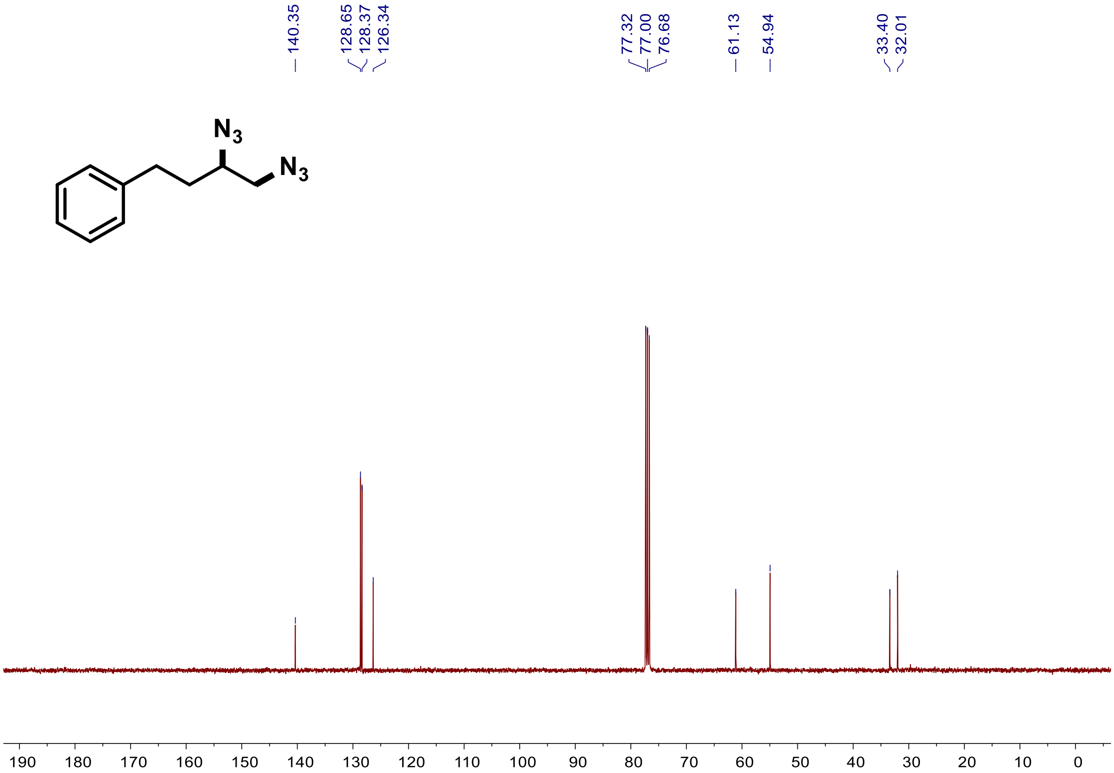

Supplementary Figure 29. ^{1}**H NMR** (500 MHz, $CDCl_3$, 298K) of **1-(2,3-diazidopropyl)-4-fluorobenzene**

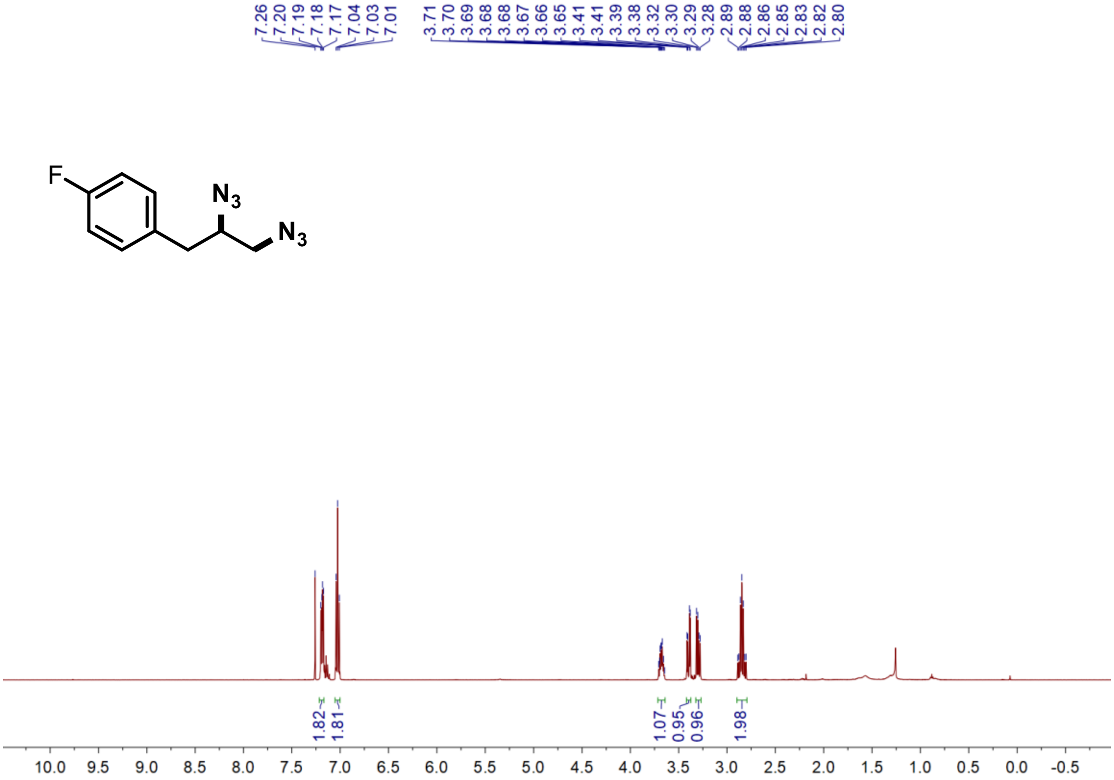

Supplementary Figure 30. ^{13}**C NMR** (101 MHz, $CDCl_3$, 298K) of **1-(2,3-diazidopropyl)-4-fluorobenzene**

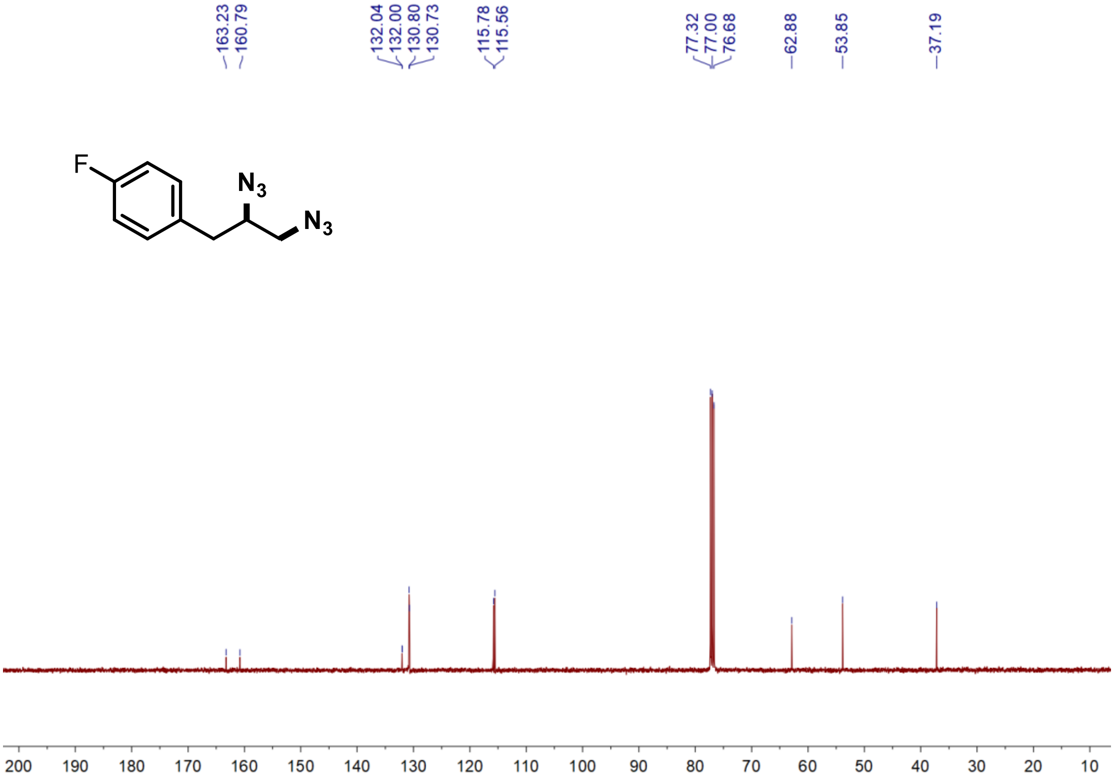

Supplementary Figure 31. **^{19}F NMR** (471 MHz, $CDCl_3$, 298K) of **1-(2,3-diazidopropyl)-4-fluorobenzene**

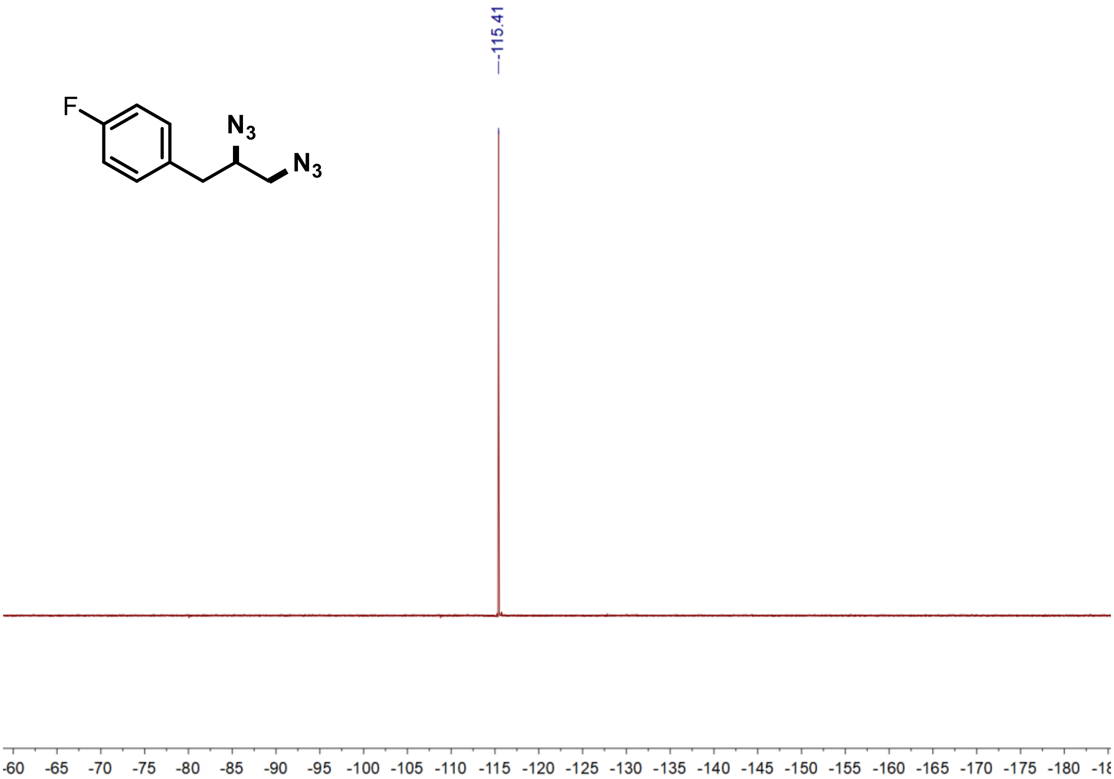

Supplementary Figure 32. **^{1}H NMR** (500 MHz, $CDCl_3$, 298K) of **1-(2,3-diazidopropyl)naphthalene**

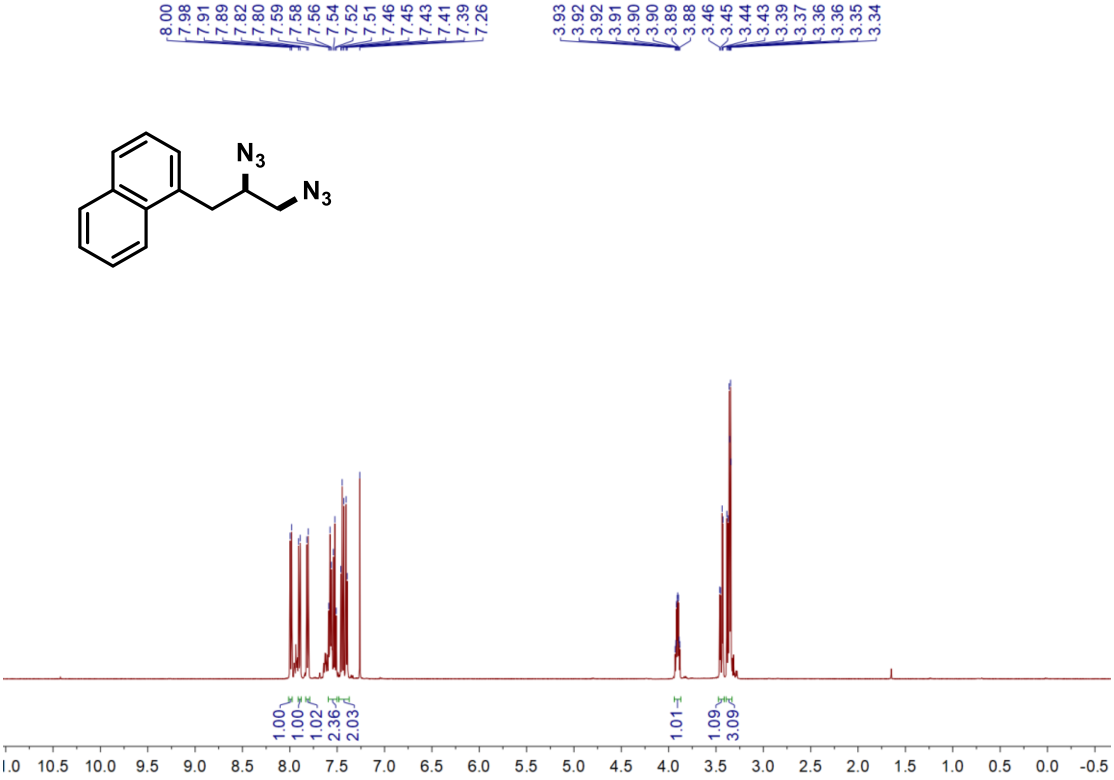

Supplementary Figure 33. ^{13}**C NMR** (101 MHz, $CDCl_3$, 298K) of **1-(2,3-diazidopropyl)naphthalene**

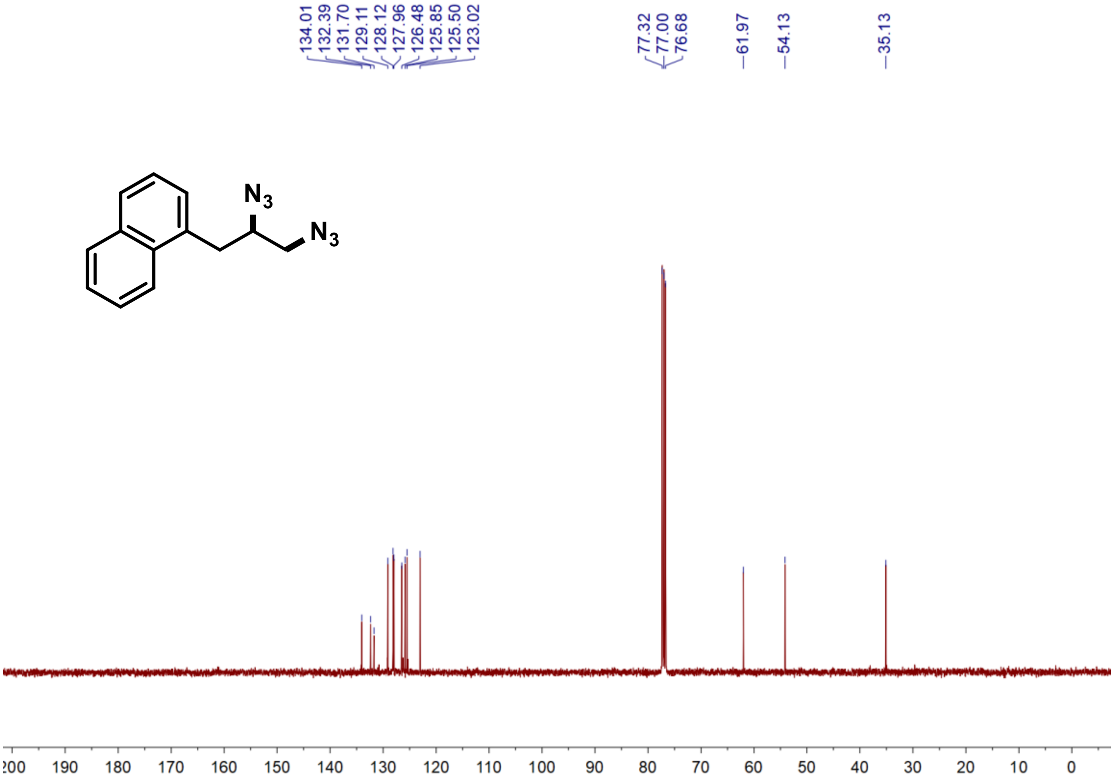

Supplementary Figure 34. ^{1}**H NMR** (500 MHz, $CDCl_3$, 298K) of **2-(4,5-diazidopentyl)benzo[b]thiophene**

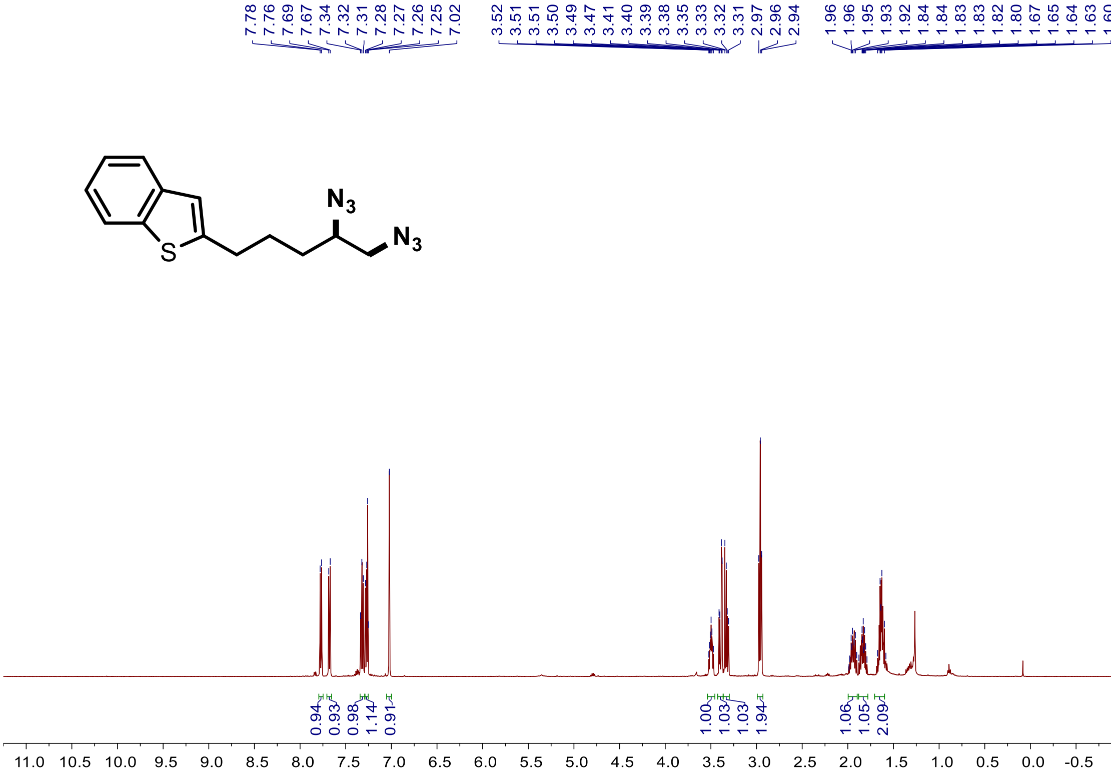

Supplementary Figure 35. ^{13}**C NMR** (101 MHz, $CDCl_3$, 298K) of **2-(4,5-diazidopentyl)benzo[b]thiophene**

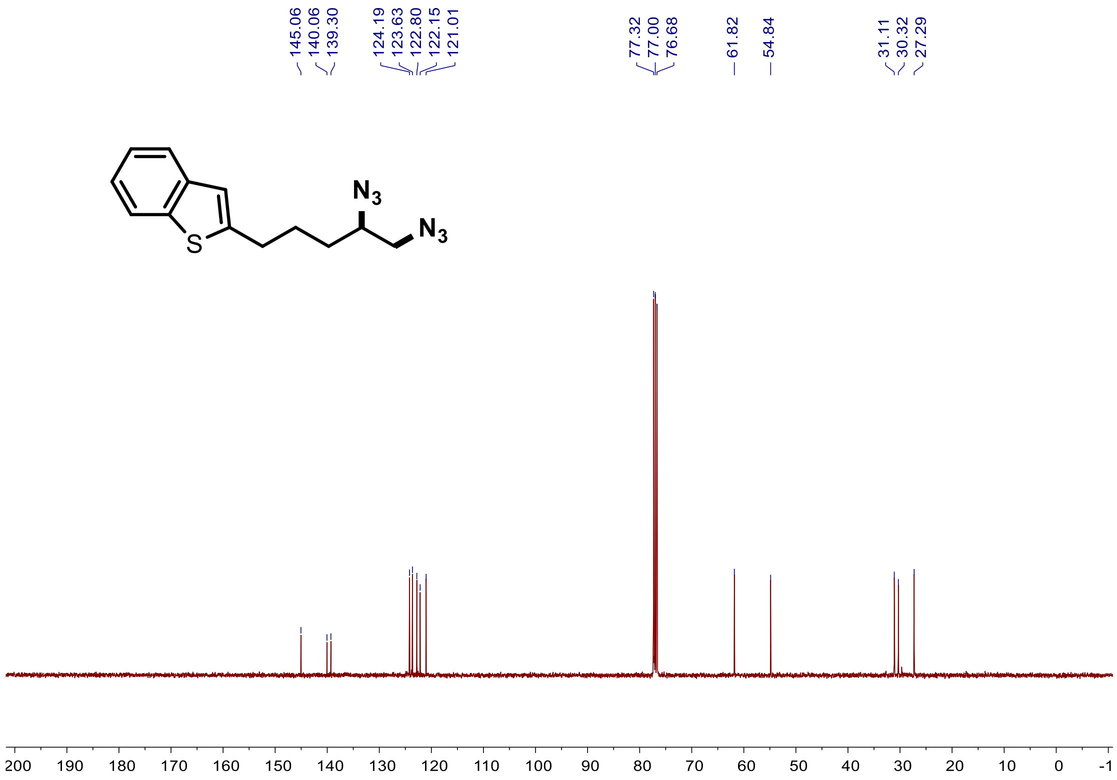

Supplementary Figure 36. ^{1}**H NMR** (500 MHz, $CDCl_3$, 298K) of **1-(2,3-diazidopropyl)-2,3,4,5,6-pentafluorobenzene**

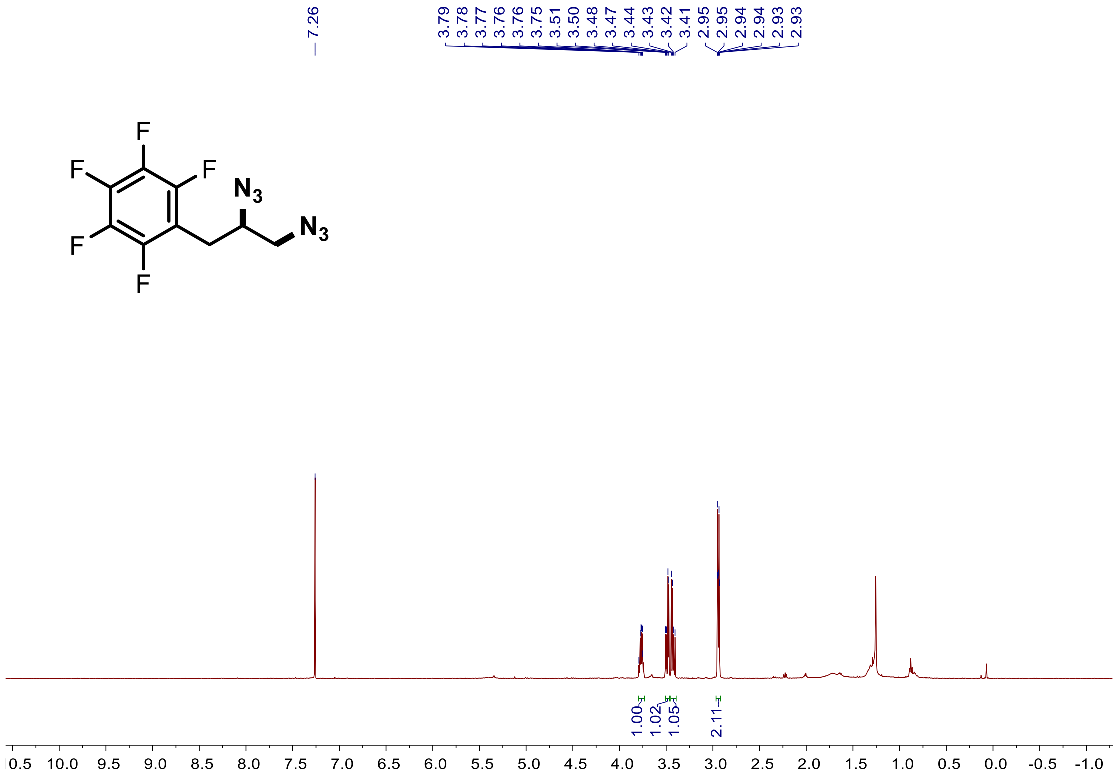

Supplementary Figure 37. ^{13}**C NMR** (101 MHz, $CDCl_3$, 298K) of **1-(2,3-diazidopropyl)-2,3,4,5,6-pentafluorobenzene**

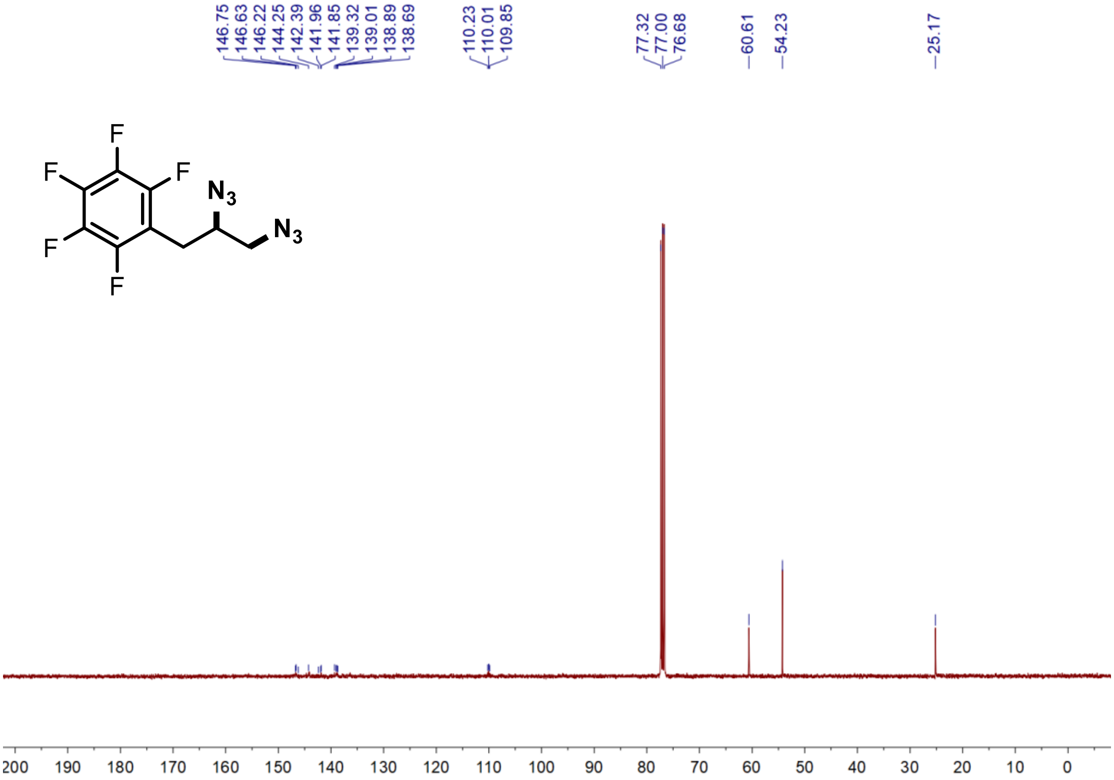

Supplementary Figure 38. ^{19}**F NMR** (471 MHz, $CDCl_3$, 298K) of **1-(2,3-diazidopropyl)-2,3,4,5,6-pentafluorobenzene**

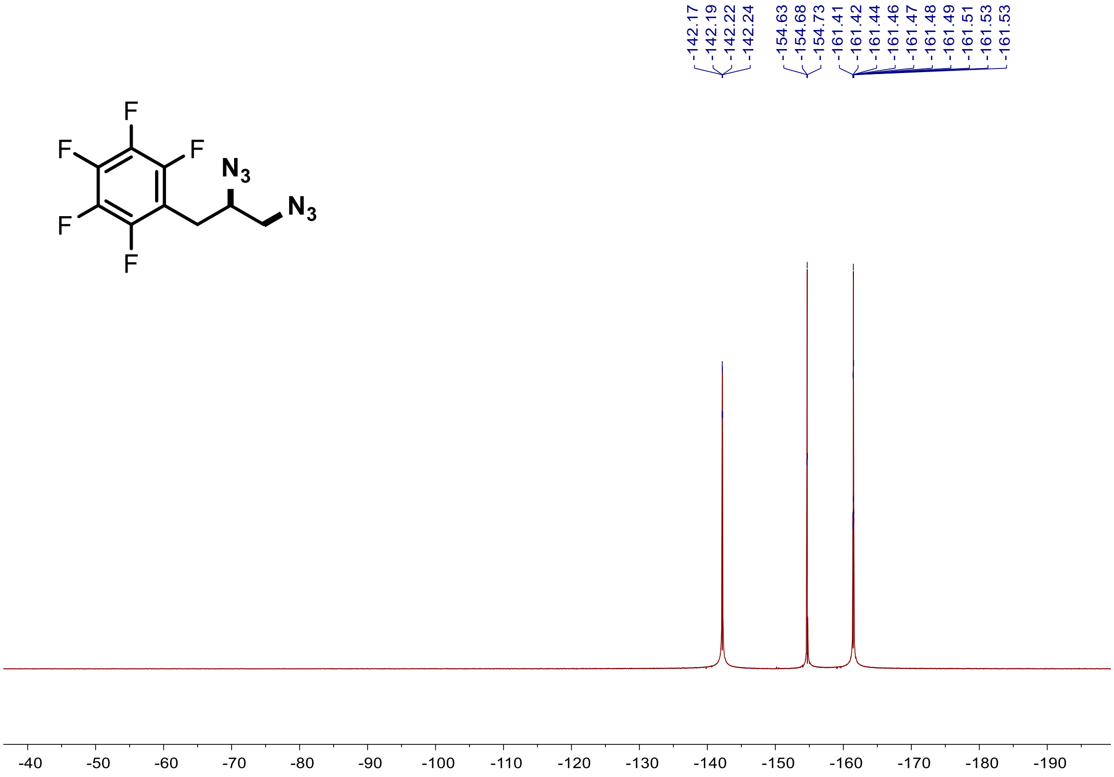

Supplementary Figure 39. ^{1}**H NMR** (500 MHz, $CDCl_3$, 298K) of **1,2-diazido-6-chlorohexane**

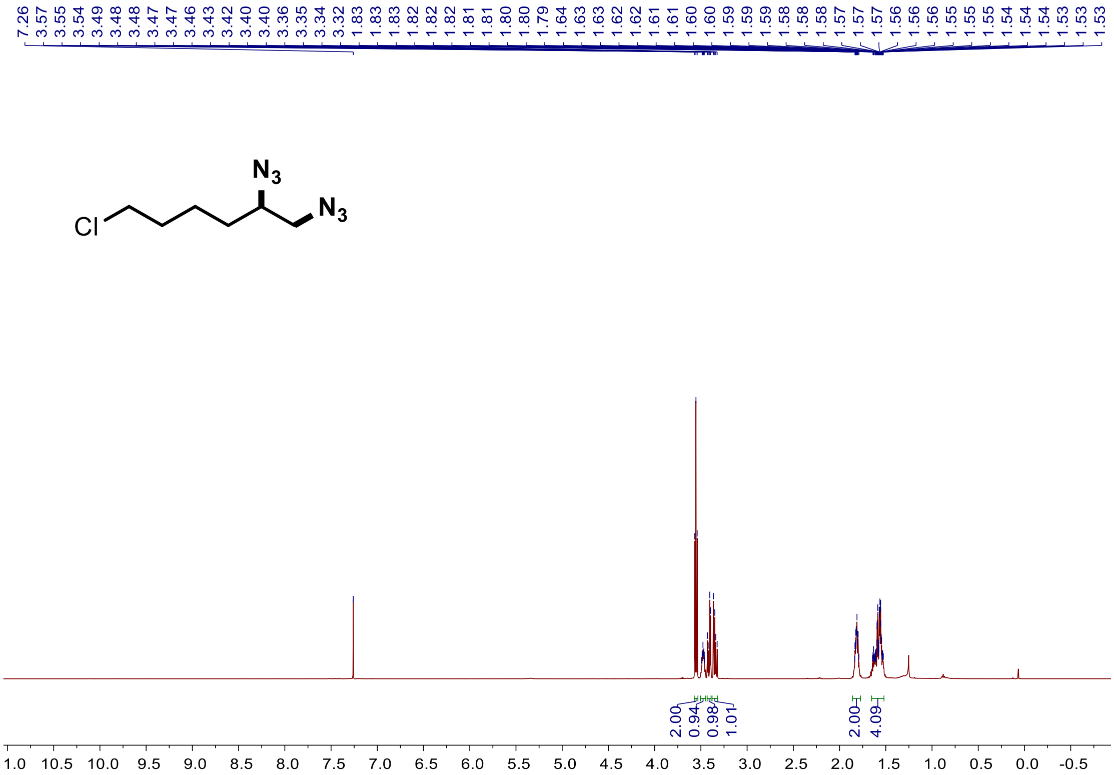

Supplementary Figure 40. ^{13}**C NMR** (101 MHz, $CDCl_3$, 298K) of **1,2-diazido-6-chlorohexane**

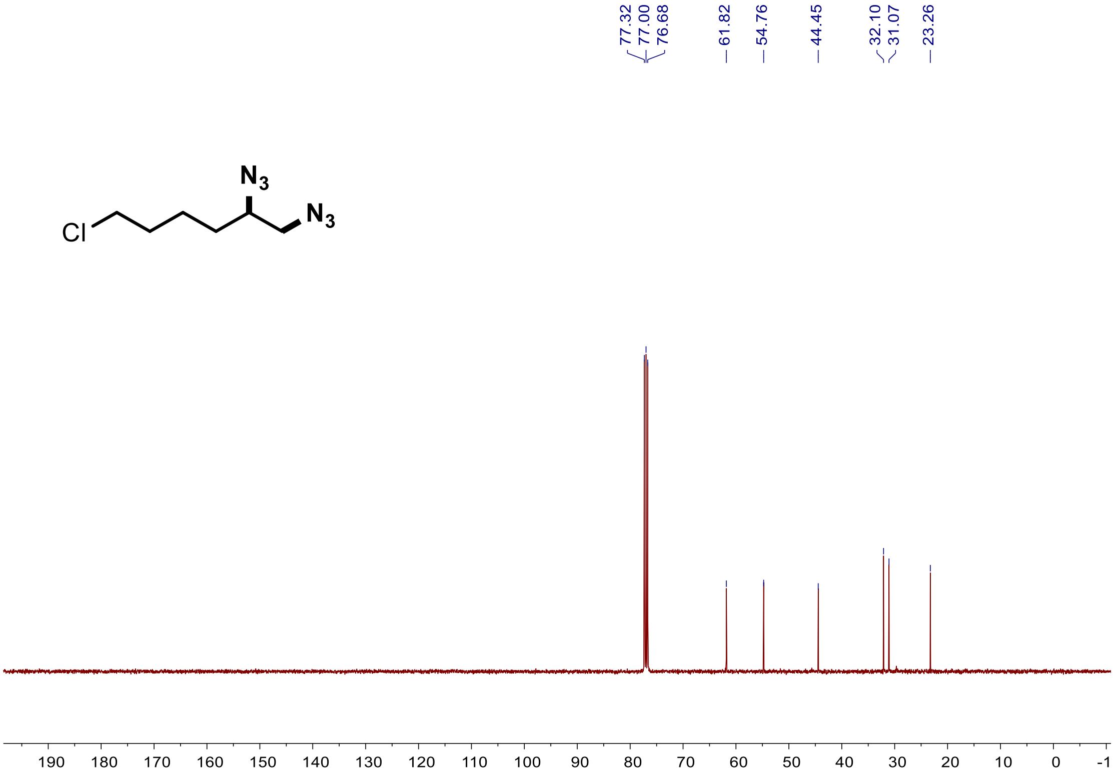

Supplementary Figure 41. ^{1}**H NMR** (500 MHz, $CDCl_3$, 298K) of **1,2-diazido-8-bromooctane**

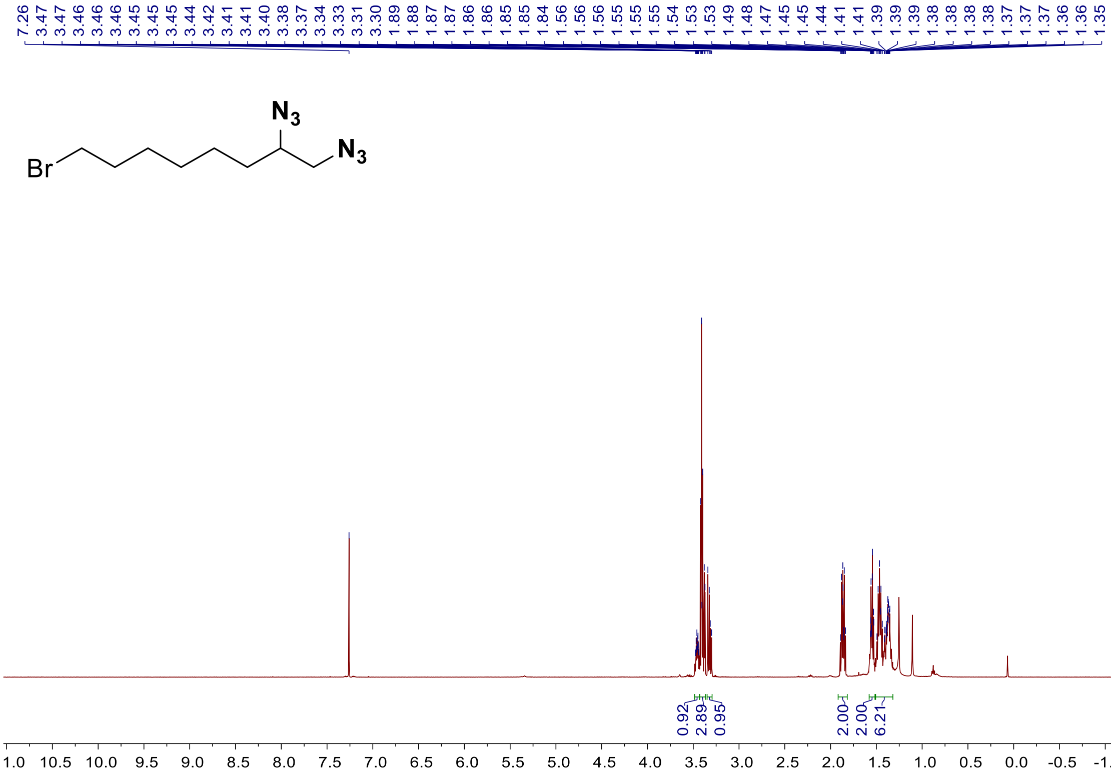

Supplementary Figure 42. ^{13}**C NMR** (101 MHz, $CDCl_3$, 298K) of **1,2-diazido-8-bromooctane**

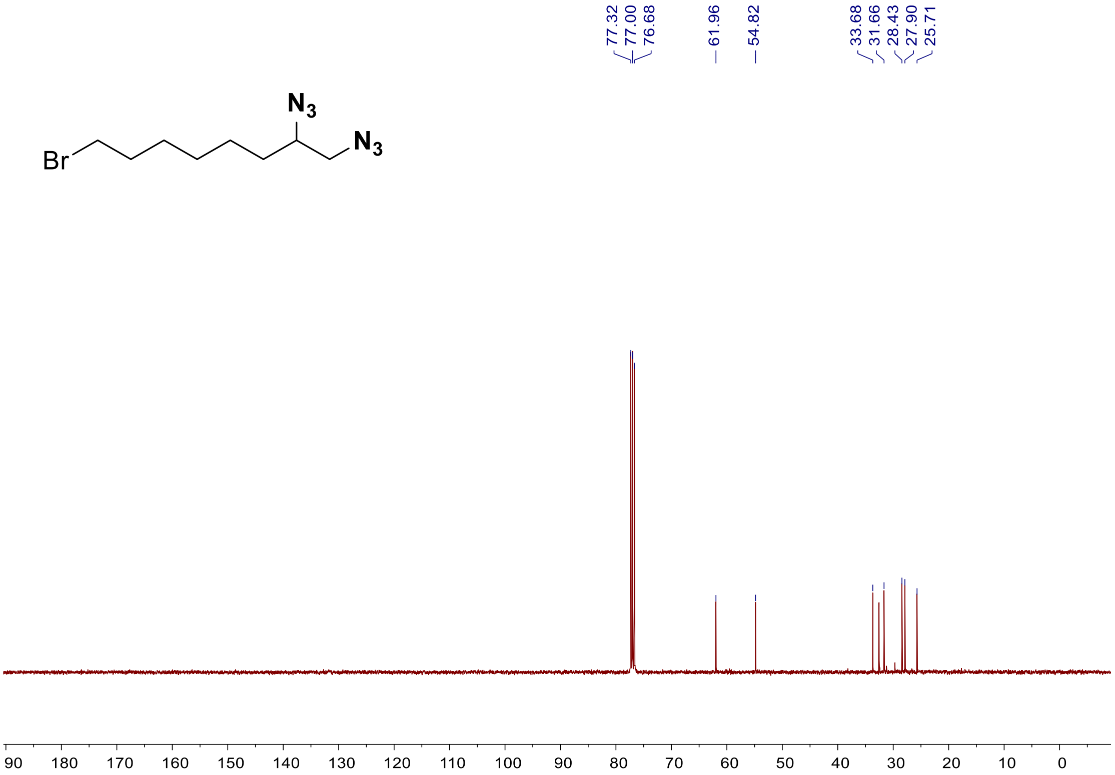

Supplementary Figure 43. ^{1}**H NMR** (500 MHz, $CDCl_3$, 298K) of **5,6-diazidohexan-1-ol**

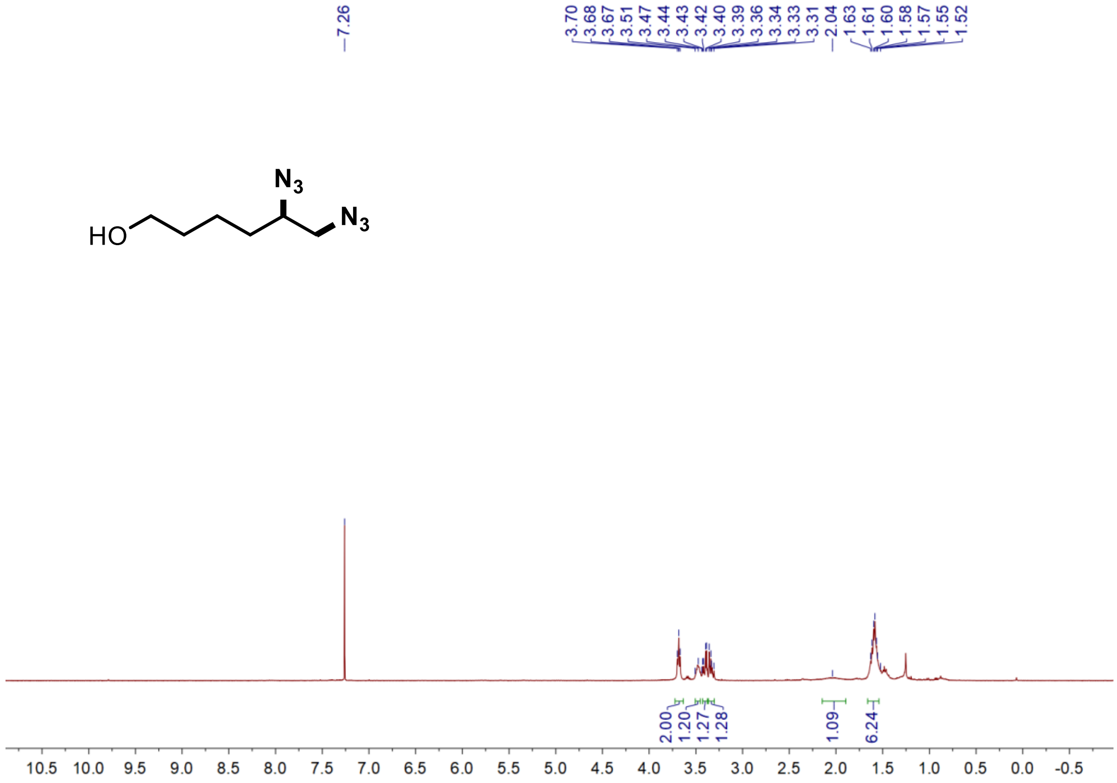

Supplementary Figure 44. ^{13}**C NMR** (101 MHz, $CDCl_3$, 298K) of **5,6-diazidohexan-1-ol**

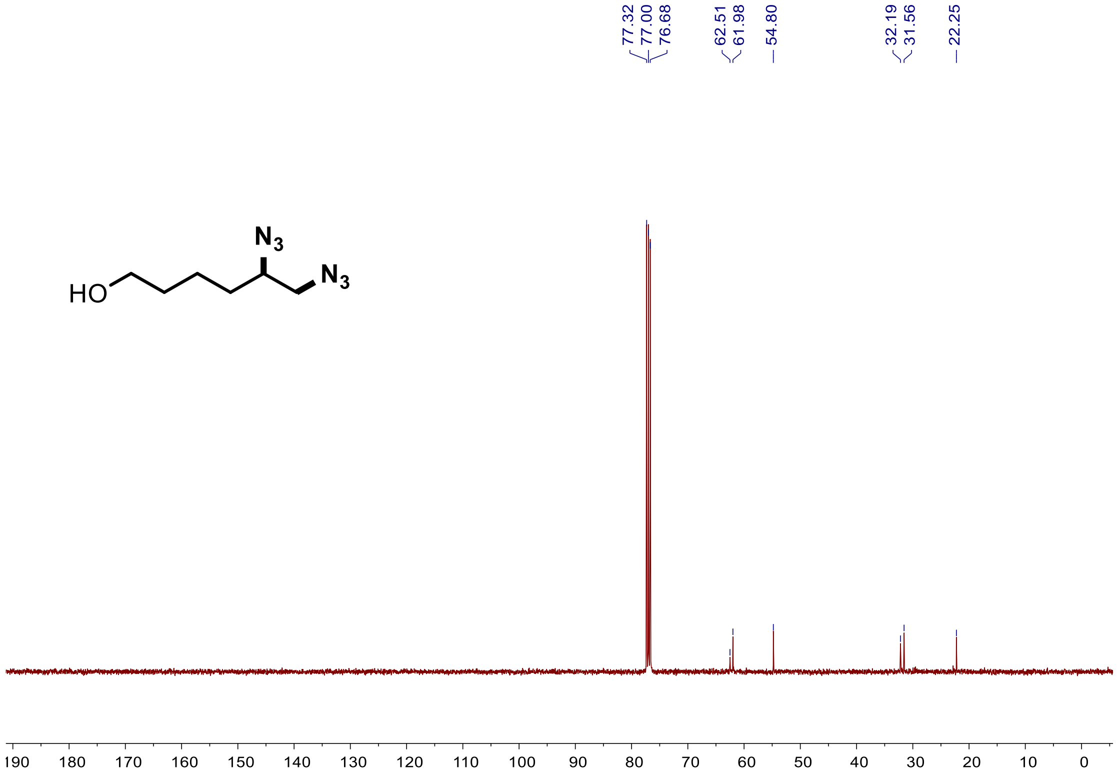

Supplementary Figure 45. ^{1}**H NMR** (500 MHz, $CDCl_3$, 298K) of **2-(2,3-diazidopropyl)isoindoline-1,3-dione**

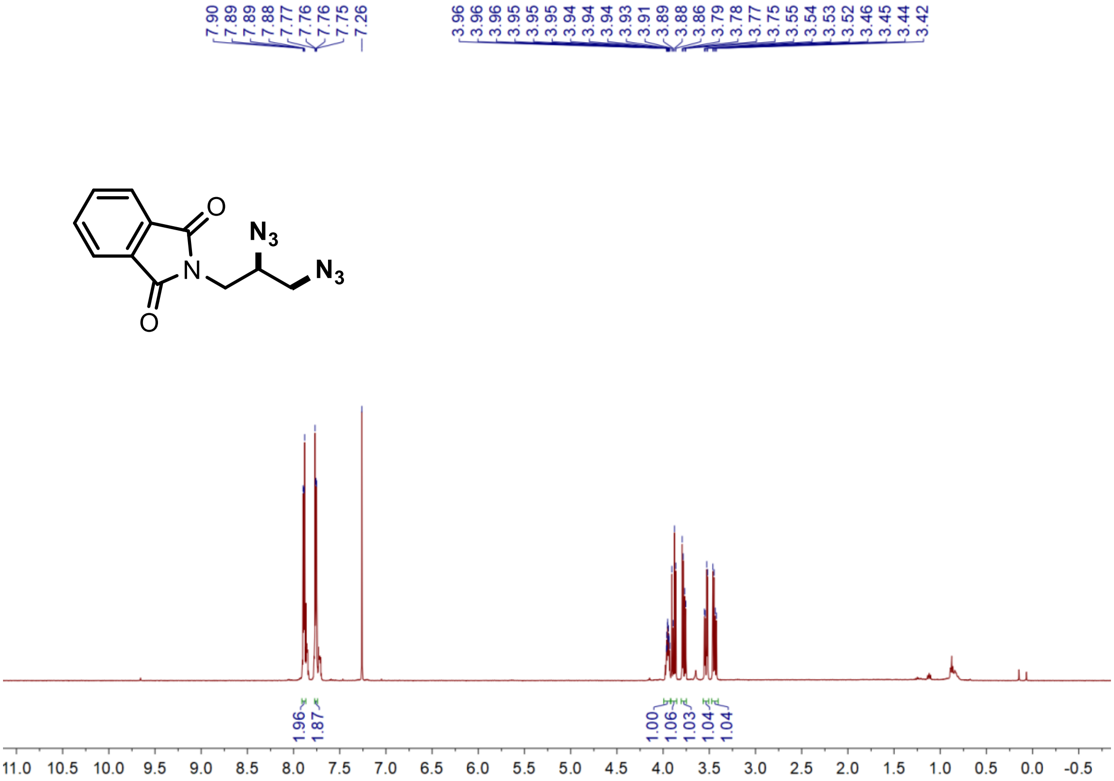

Supplementary Figure 46. ^{13}**C NMR** (101 MHz, $CDCl_3$, 298K) of **2-(2,3-diazidopropyl)isoindoline-1,3-dione**

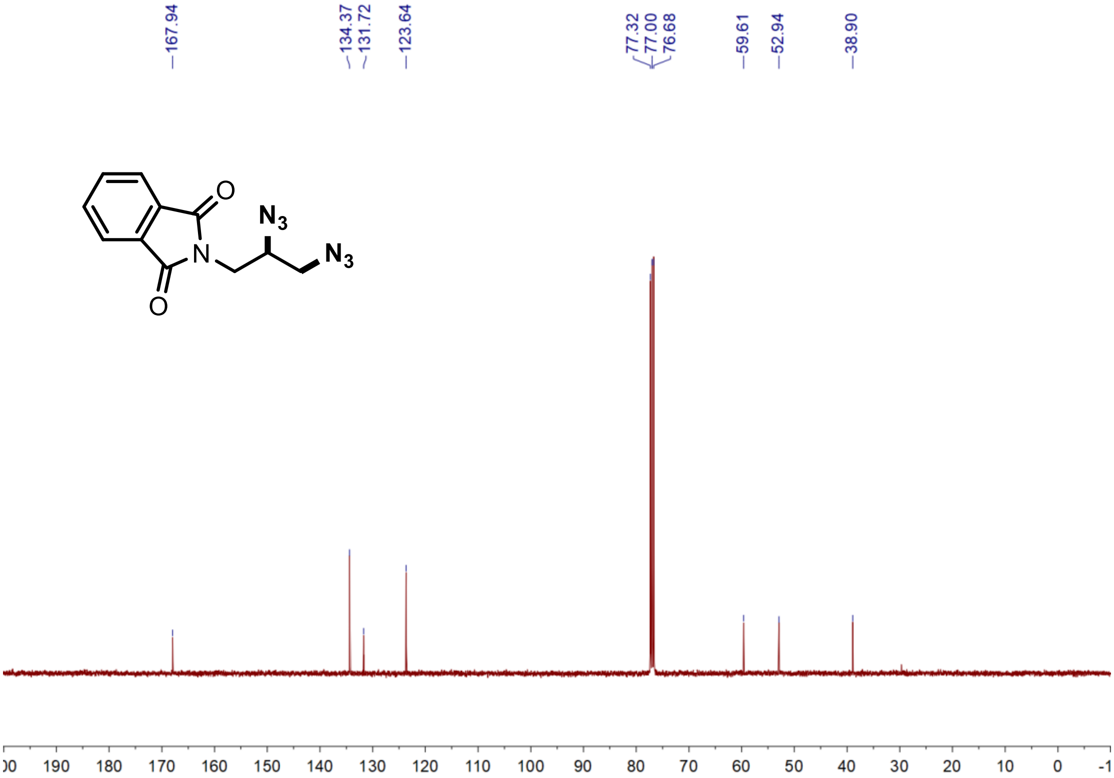

Supplementary Figure 47. **^{1}H NMR** (500 MHz, $CDCl_3$, 298K) of **4,5-diazido-N-phenylpentanamide**

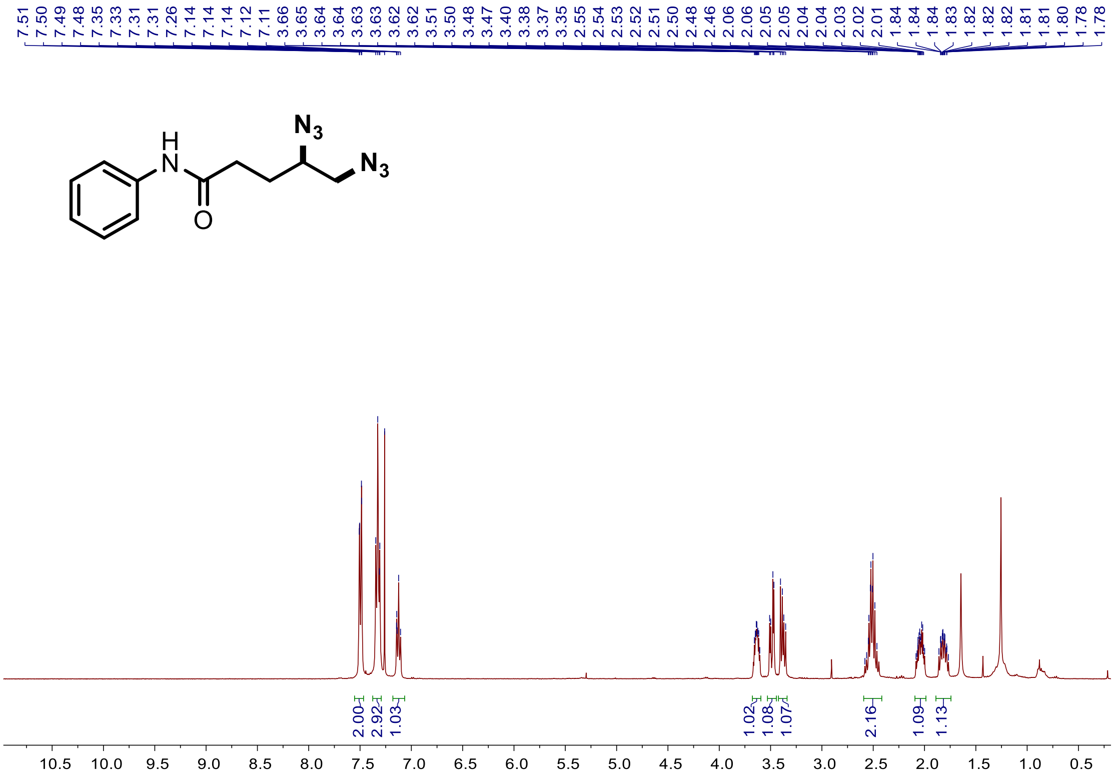

Supplementary Figure 48. **^{13}C NMR** (126 MHz, $CDCl_3$, 298K) of **4,5-diazido-N-phenylpentanamide**

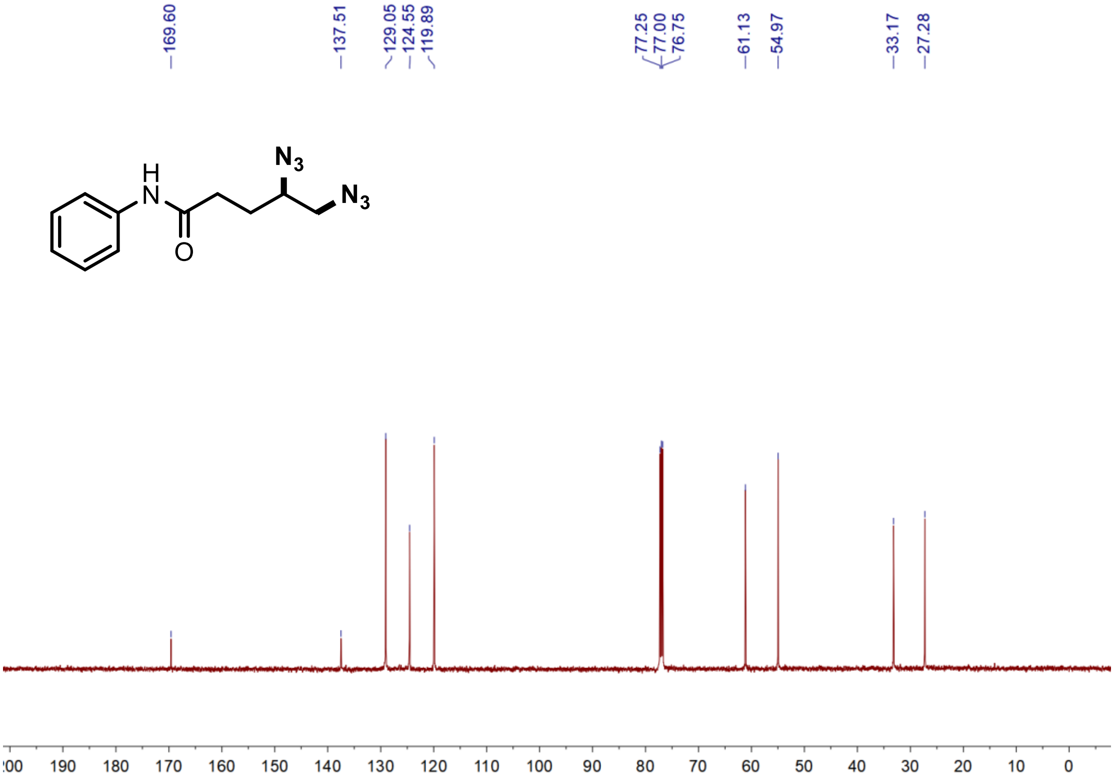

Supplementary Figure 49. ^{1}**H NMR** (500 MHz, $CDCl_3$, 298K) of **S-phenyl-4,5-diazidopentanethioate**

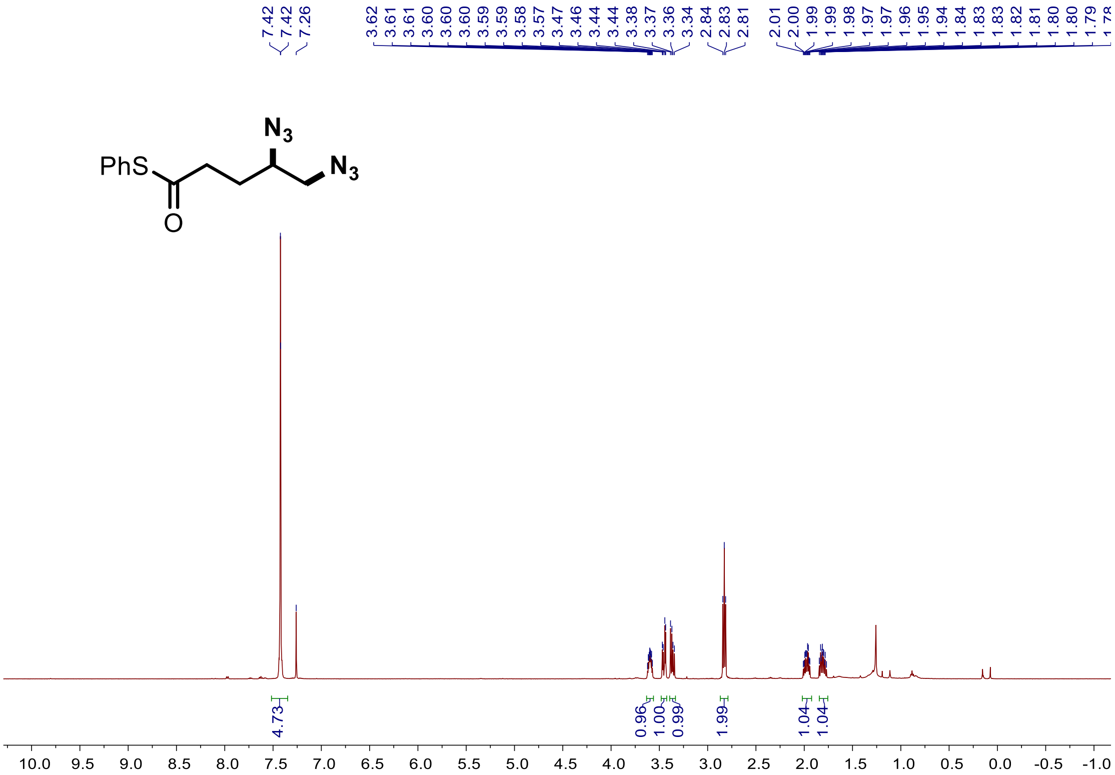

Supplementary Figure 50. ^{13}**C NMR** (101 MHz, $CDCl_3$, 298K) of **S-phenyl-4,5-diazidopentanethioate**

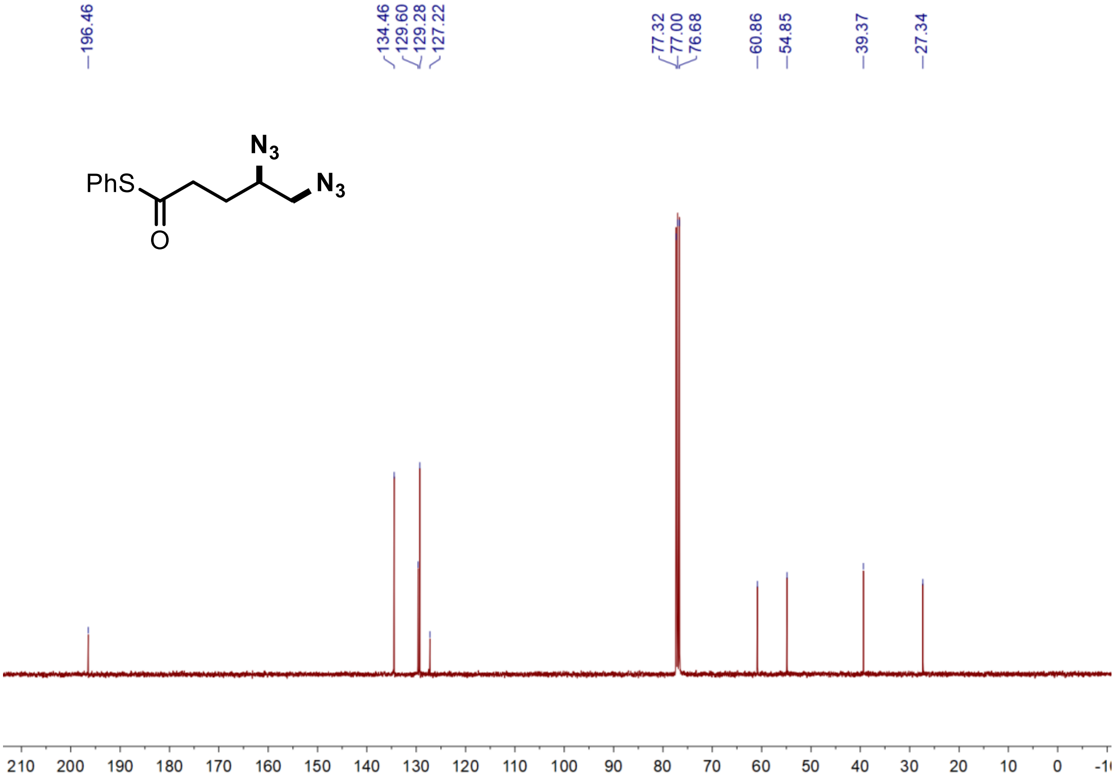

Supplementary Figure 51. ^{1}H **NMR** (500 MHz, $CDCl_3$, 298K) of **(2,3-diazidopropyl)dimethyl(phenyl)silane**

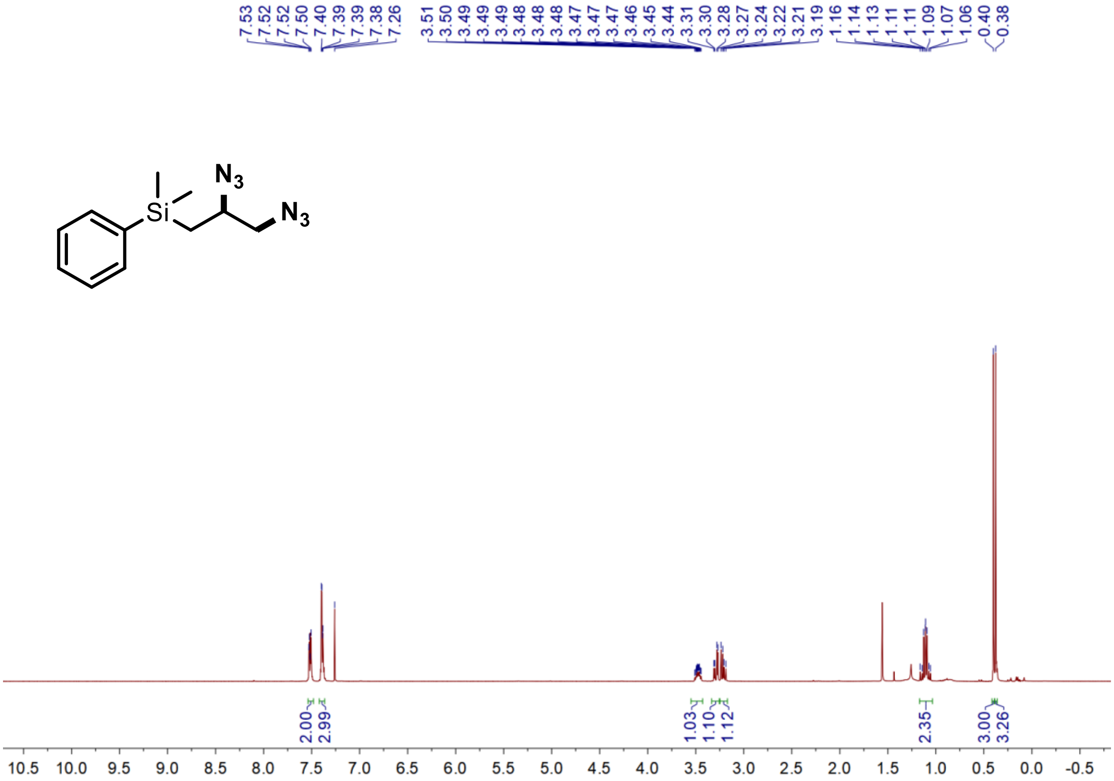

Supplementary Figure 52. ^{13}C **NMR** (126 MHz, $CDCl_3$, 298K) of **(2,3-diazidopropyl)dimethyl(phenyl)silane**

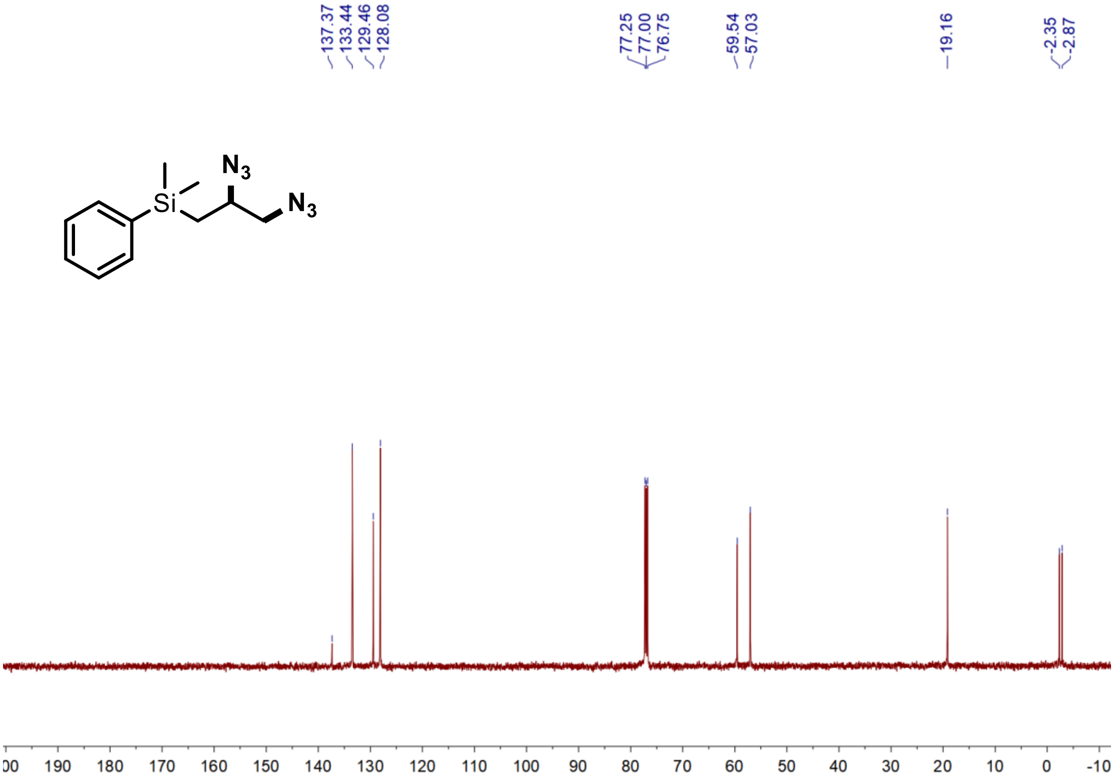

Supplementary Figure 53. **^{1}H NMR** (500 MHz, $CDCl_3$, 298K) of **4,5-diazidooctane**

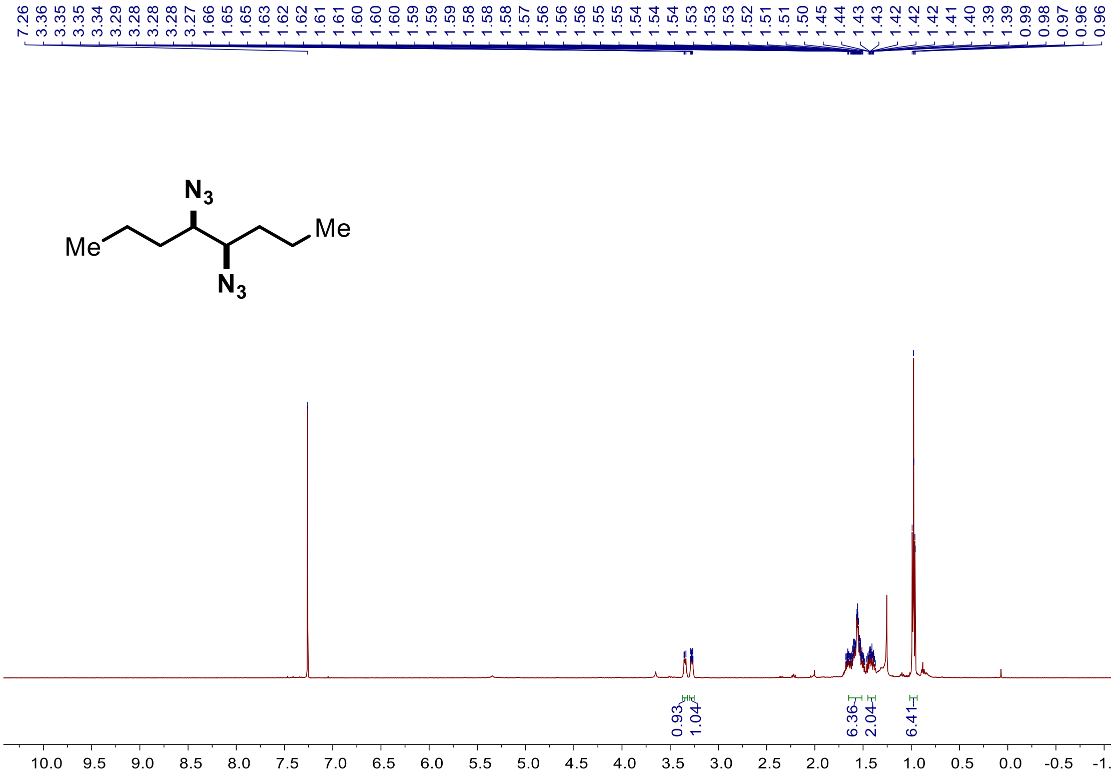

Supplementary Figure 54. **^{13}C NMR** (101 MHz, $CDCl_3$, 298K) of **4,5-diazidooctane**

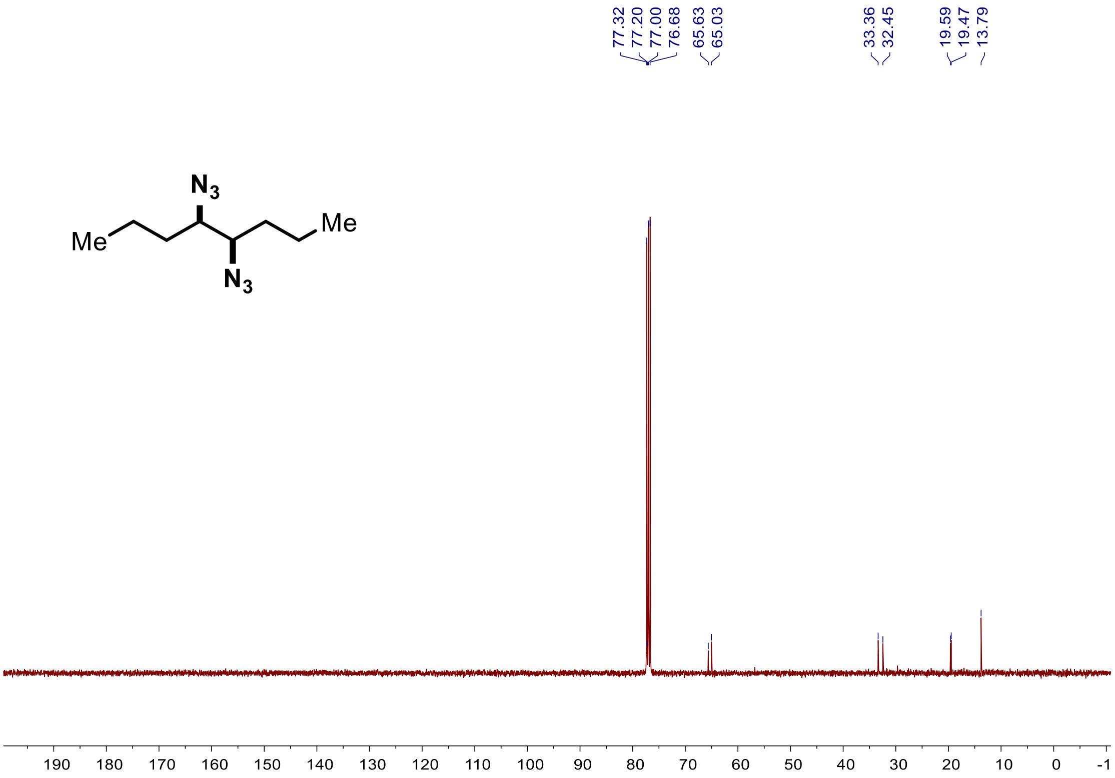

Supplementary Figure 55. ^{1}**H NMR** (500 MHz, $CDCl_3$, 298K) of **1,2-diazidocyclooctane**

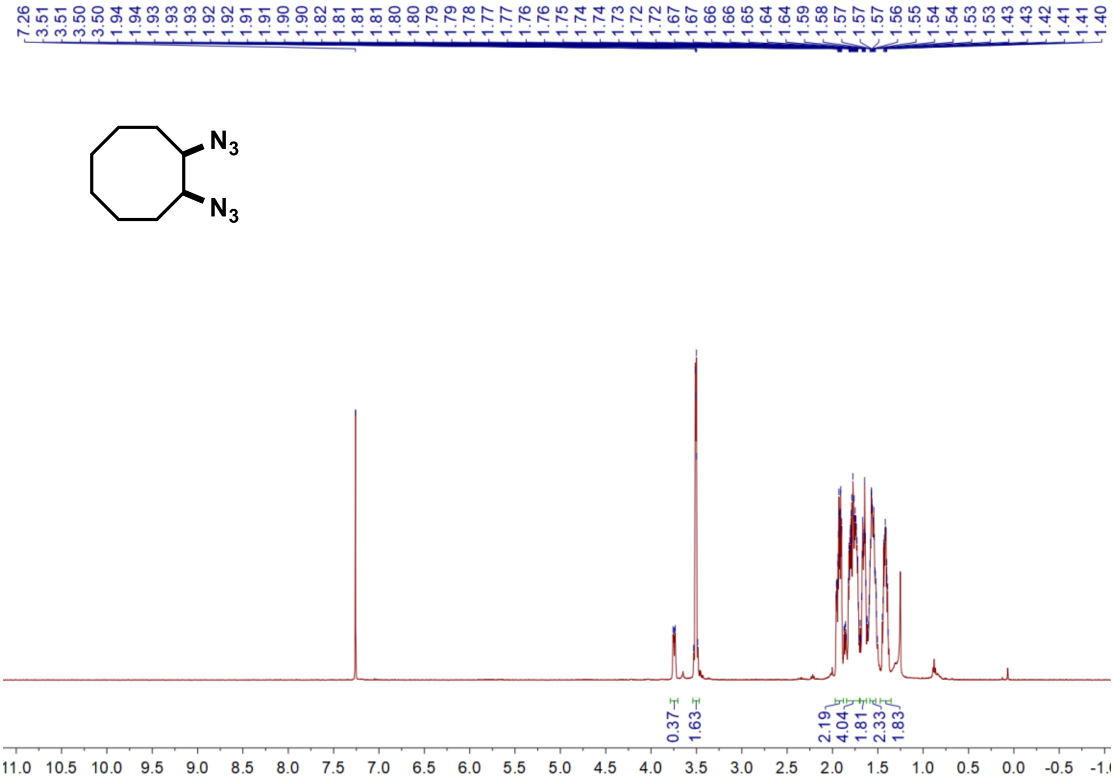

Supplementary Figure 56. ^{13}**C NMR** (101 MHz, $CDCl_3$, 298K) of **1,2-diazidocyclooctane**

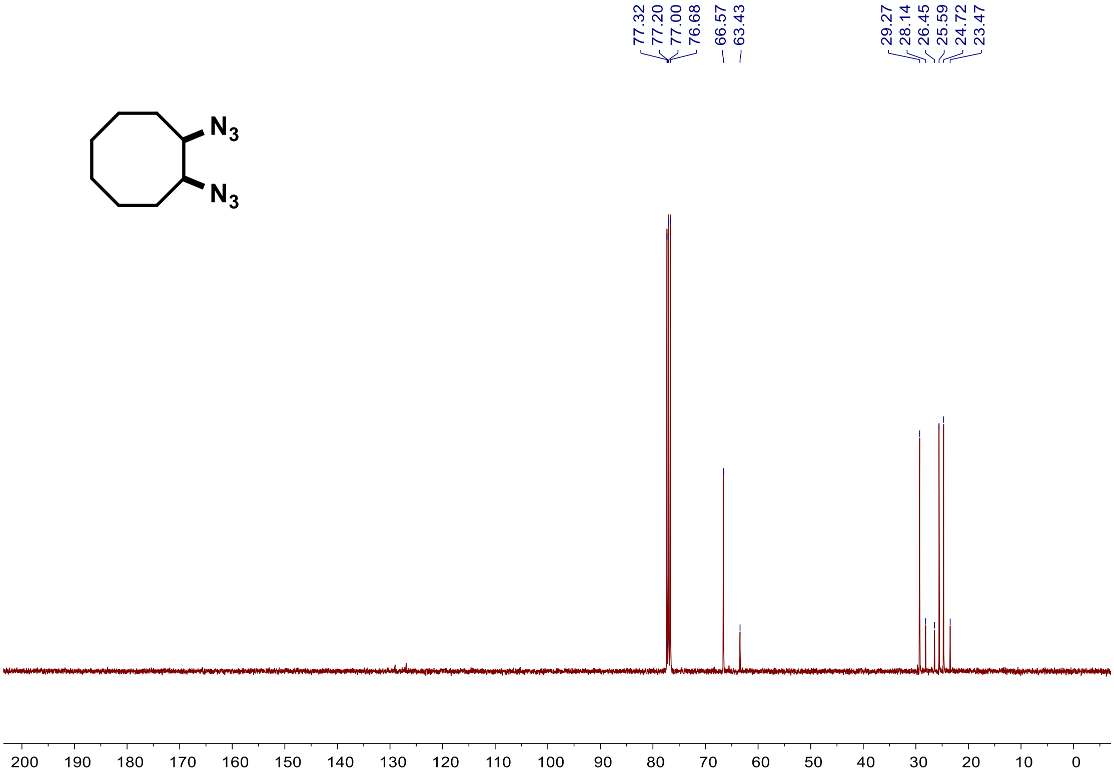

Supplementary Figure 57. ^{1}H NMR (500 MHz, $CDCl_3$, 298K) of **3,4-diazido-4-methylpentyl)benzene**

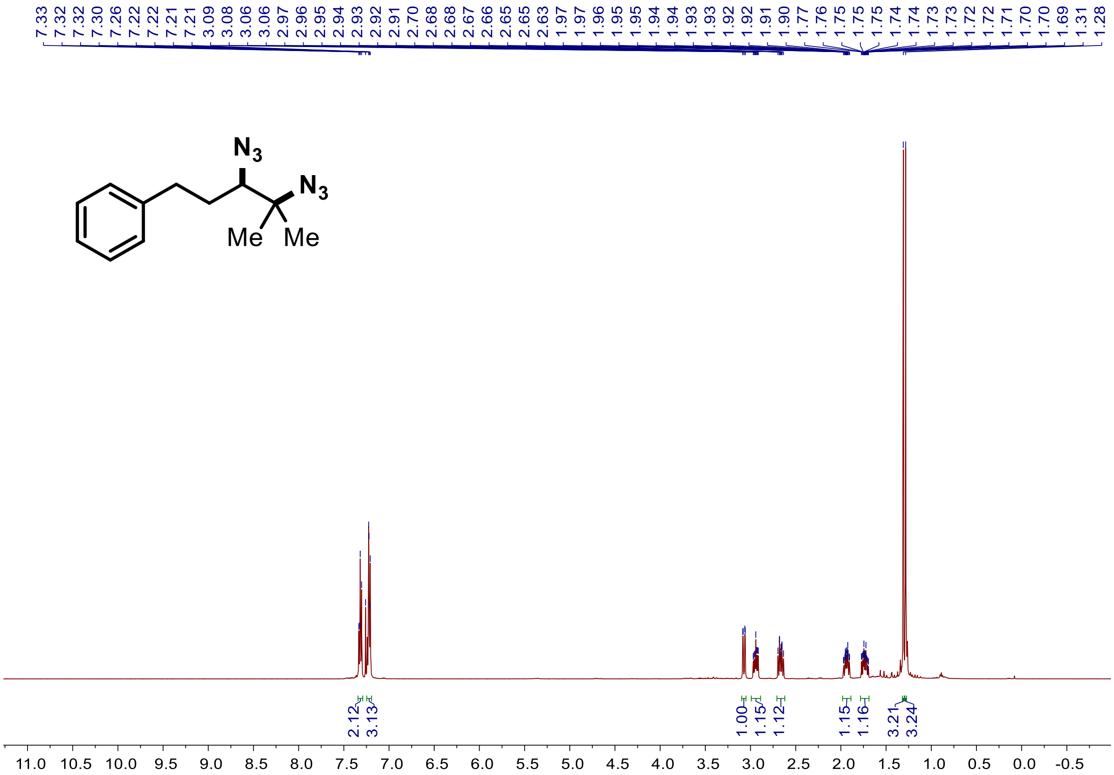

Supplementary Figure 58. ^{13}C NMR (101 MHz, $CDCl_3$, 298K) of **3,4-diazido-4-methylpentyl)benzene**

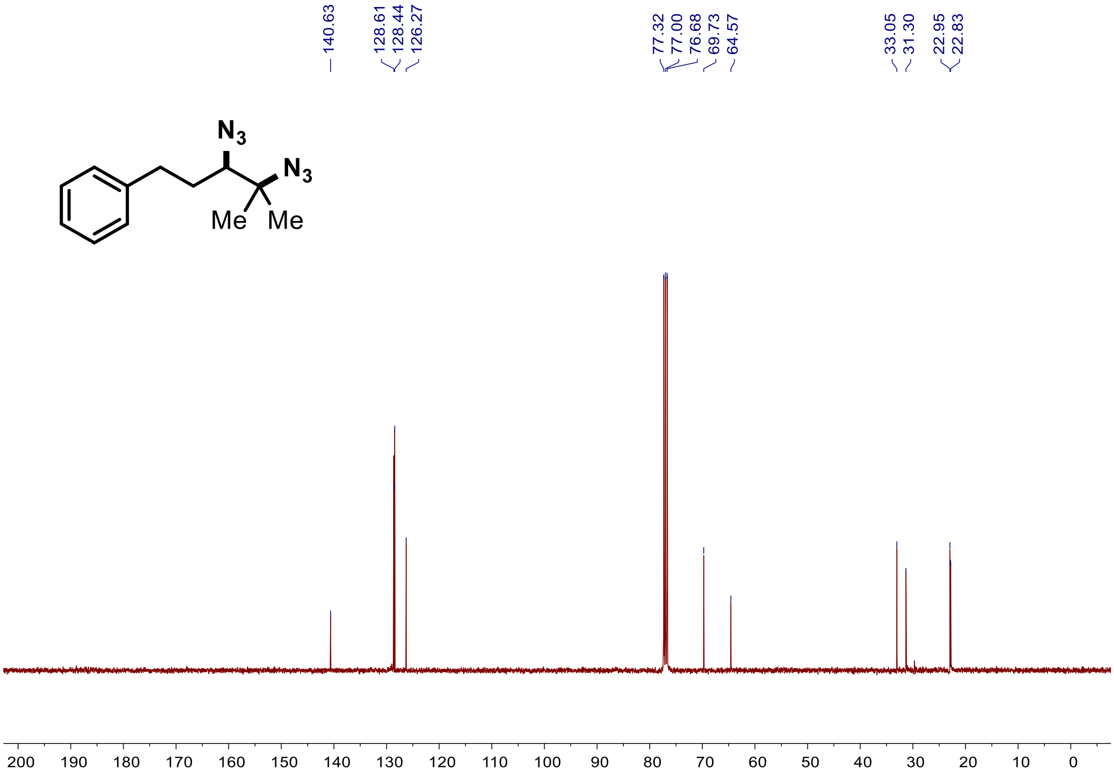

Supplementary Figure 59. ^{1}**H NMR** (500 MHz, $CDCl_3$, 298K) of **1,2-diazidoethyl)benzene**

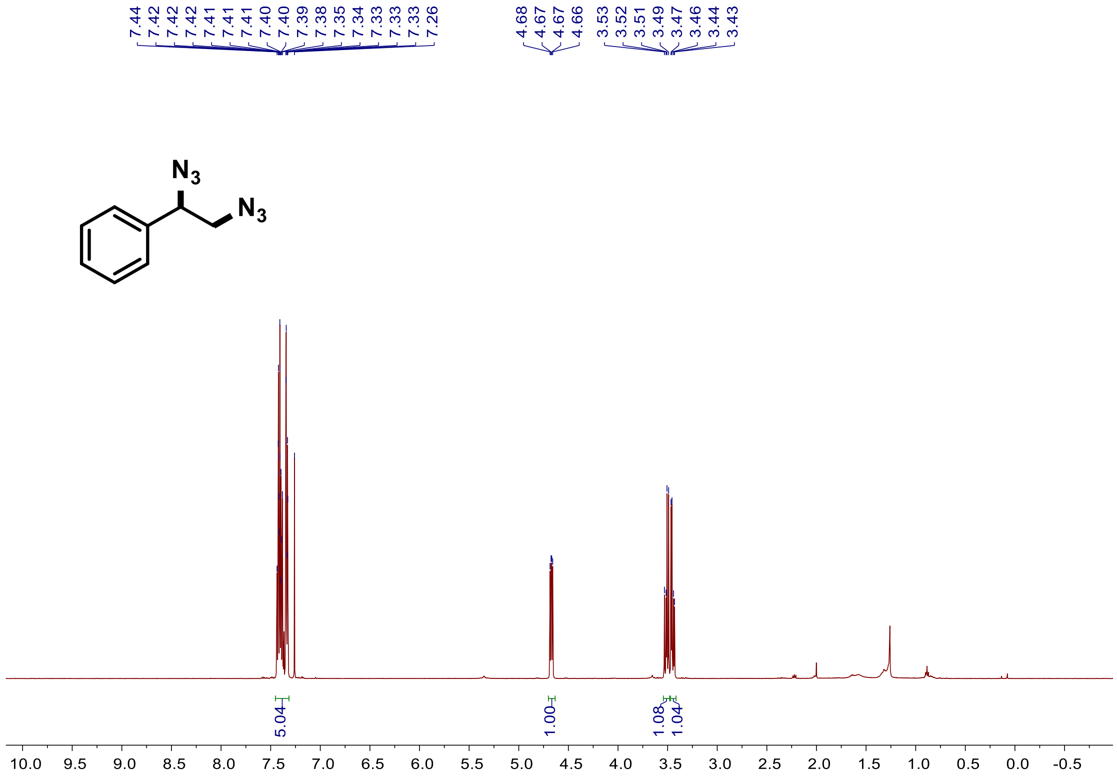

Supplementary Figure 60. ^{13}**C NMR** (101 MHz, $CDCl_3$, 298K) of **1,2-diazidoethyl)benzene**

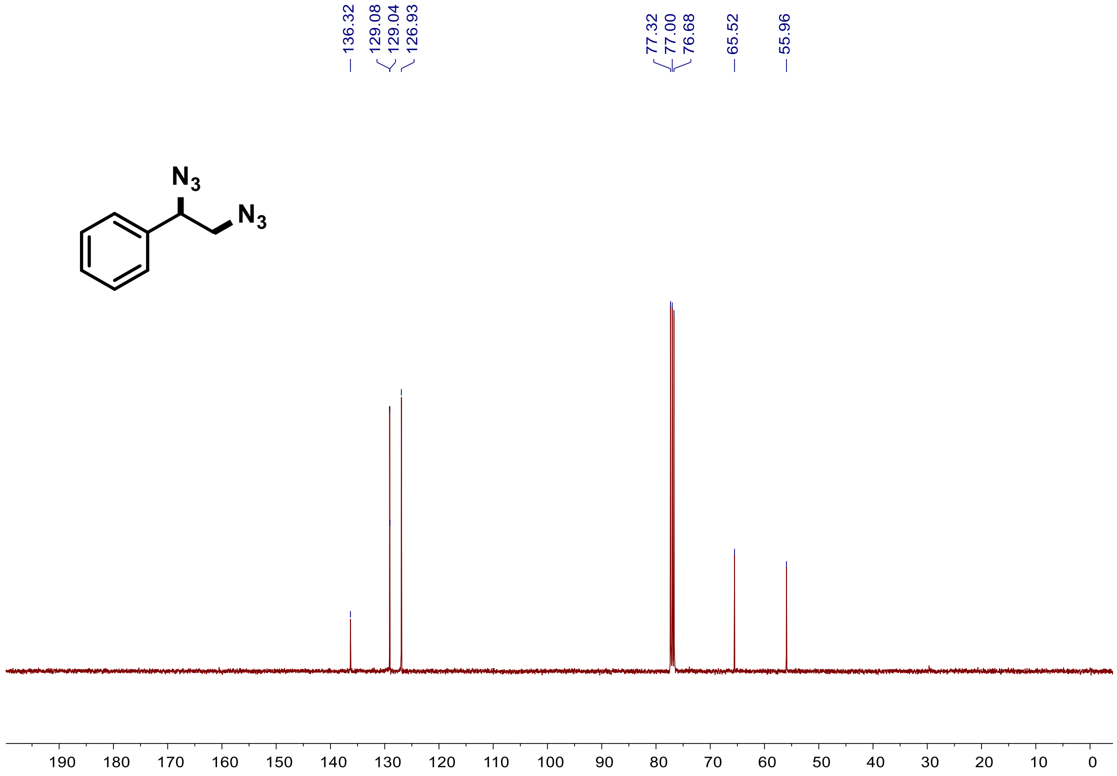

Supplementary Figure 61. ^{1}**H NMR** (500 MHz, $CDCl_3$, 298K) of **(1,2-diazidoethyl)-4-methoxybenzene**

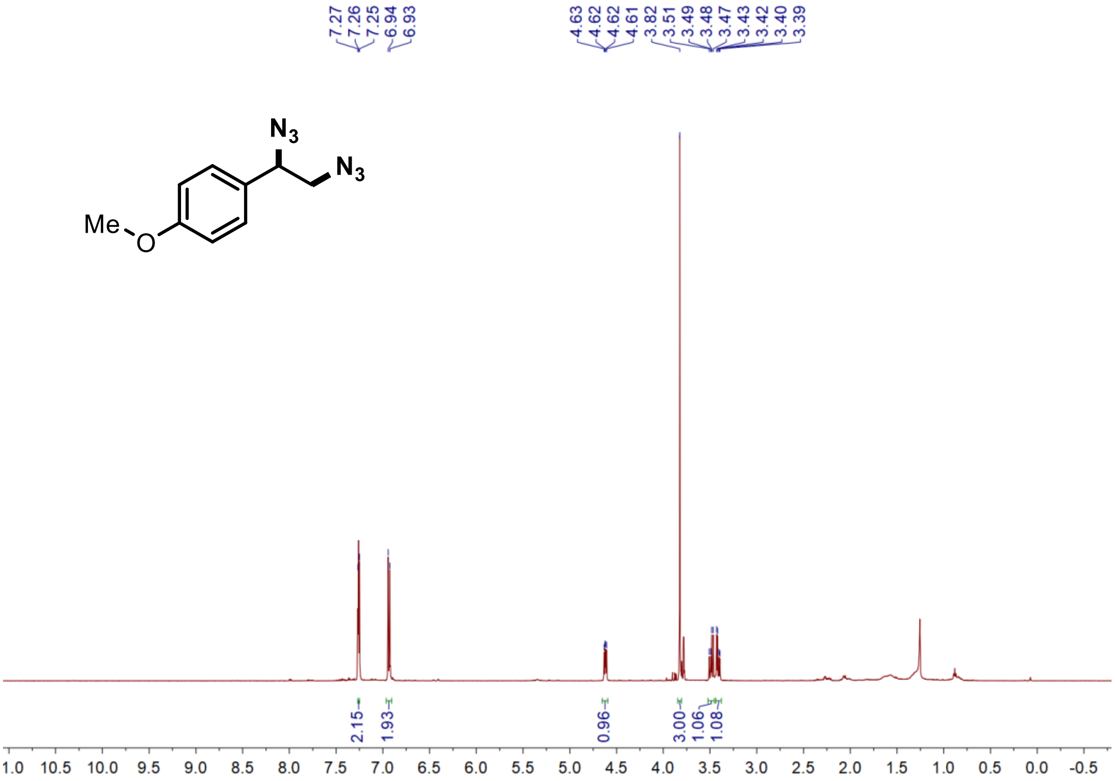

Supplementary Figure 62 ^{13}**C NMR** (101 MHz, $CDCl_3$, 298K) of **(1,2-diazidoethyl)-4-methoxybenzene**

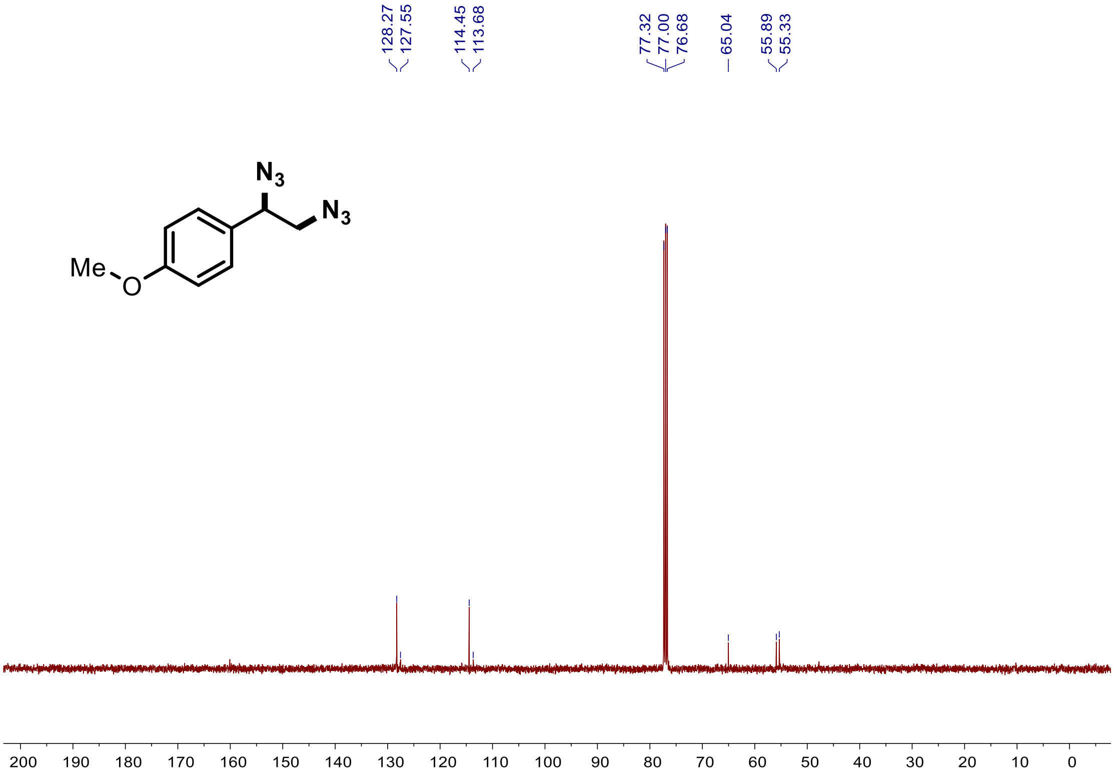

Supplementary Figure 63. ^{1}**H NMR** (500 MHz, $CDCl_3$, 298K) of **4-(1,2-diazidoethyl)phenyl acetate**

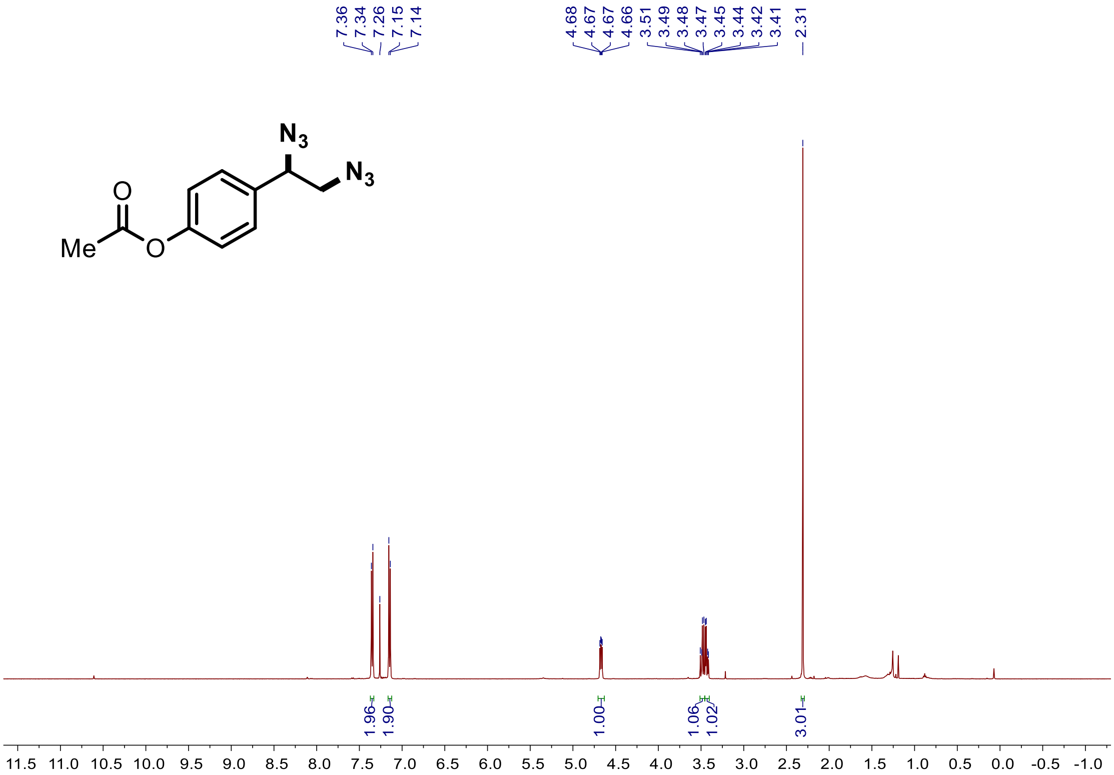

Supplementary Figure 64. ^{13}**C NMR** (101 MHz, $CDCl_3$, 298K) of **4-(1,2-diazidoethyl)phenyl acetate**

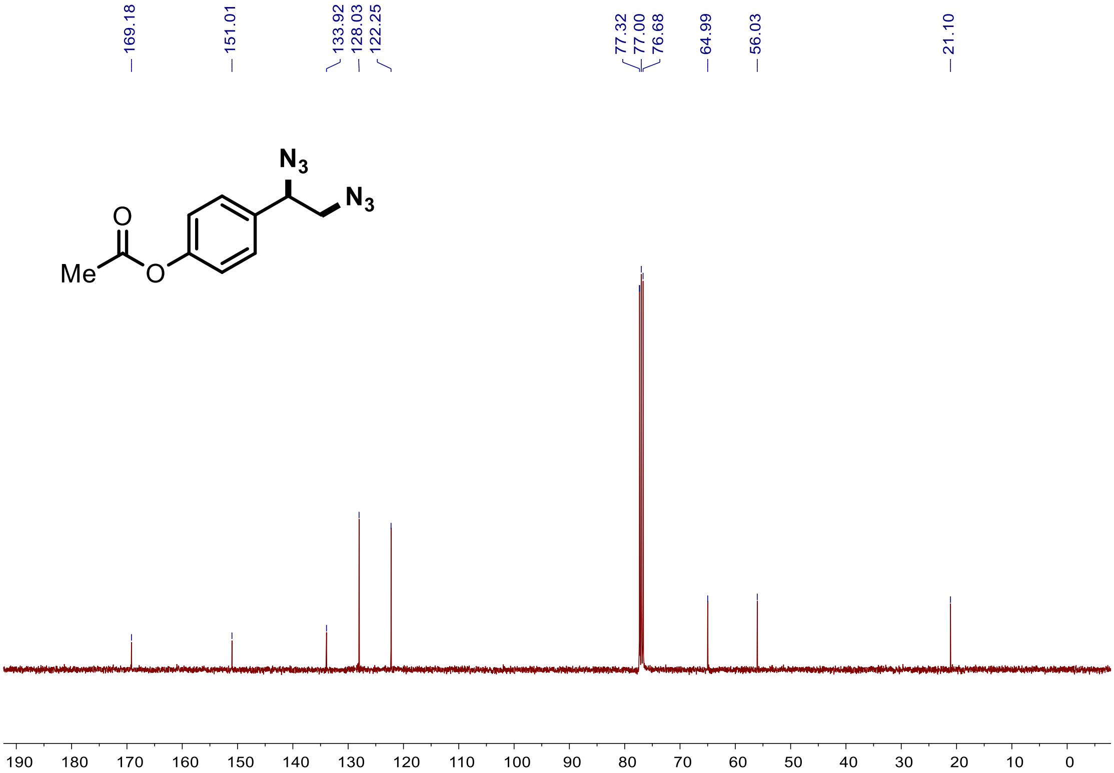

Supplementary Figure 65. **^{1}H NMR** (500 MHz, $CDCl_3$, 298K) of **1-bromo-4-(1,2-diazidoethyl)benzene**

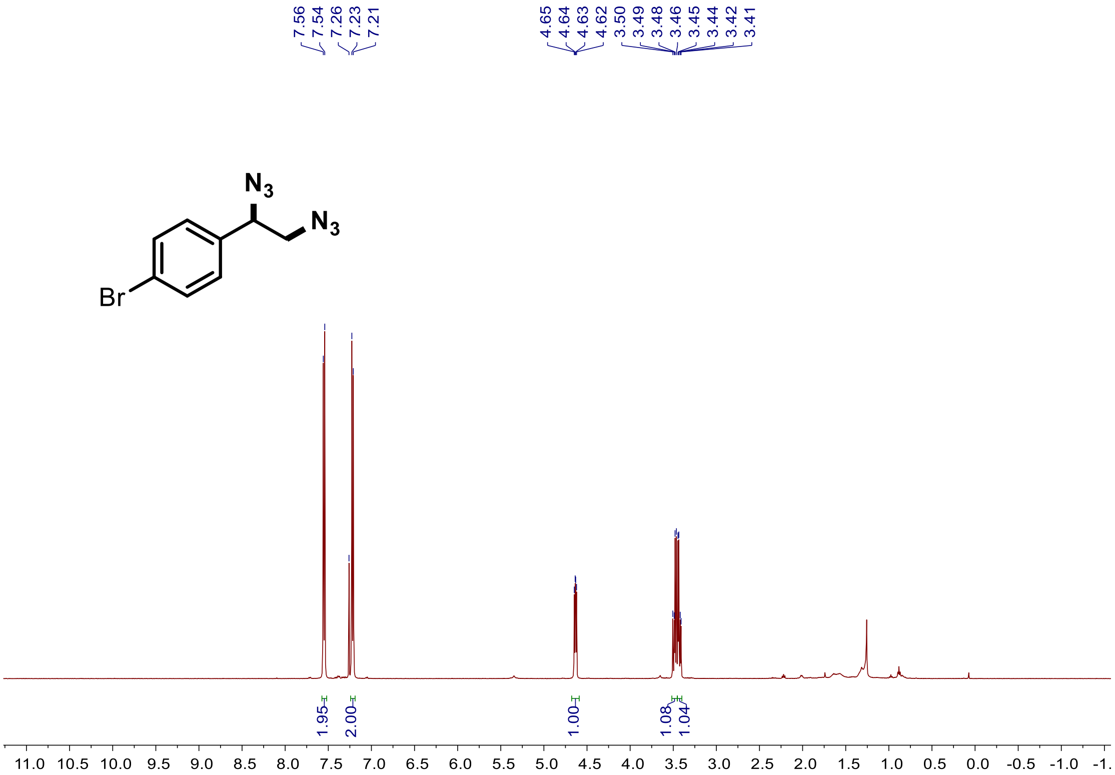

Supplementary Figure 66. **^{13}C NMR** (101 MHz, $CDCl_3$, 298K) of **1-bromo-4-(1,2-diazidoethyl)benzene**

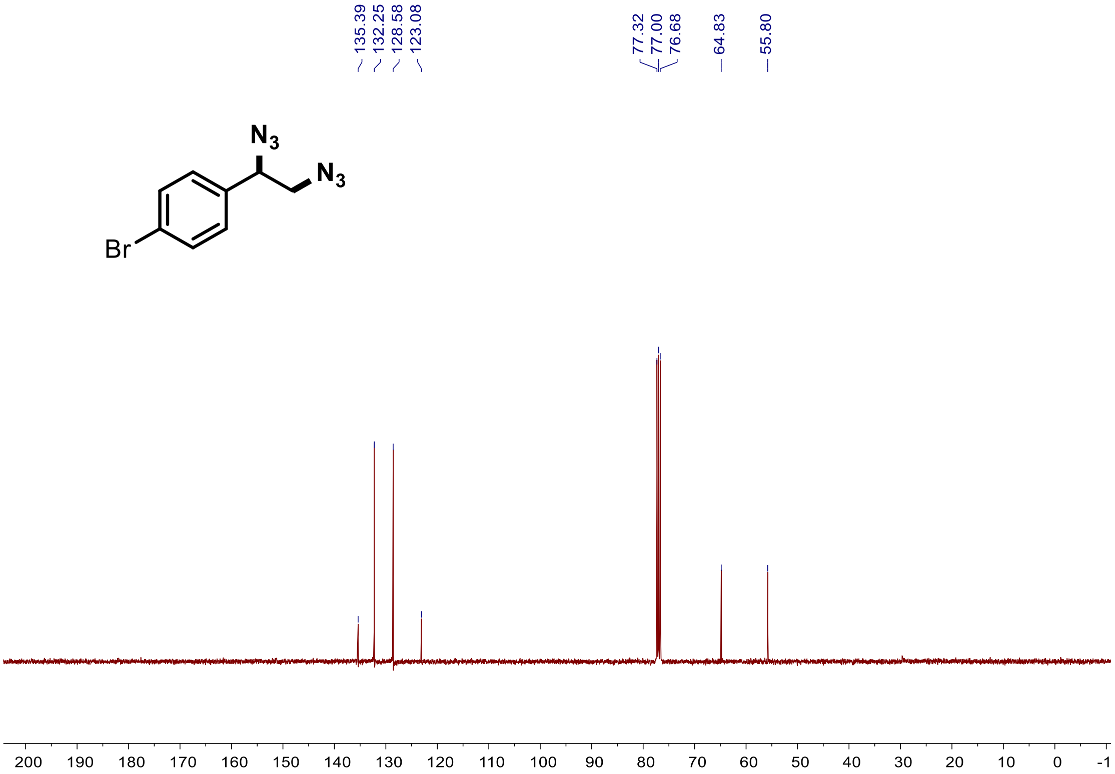

Supplementary Figure 67. ^{1}**H NMR** (500 MHz, $CDCl_3$, 298K) of **1-chloro-3-(1,2-diazidoethyl)benzene**

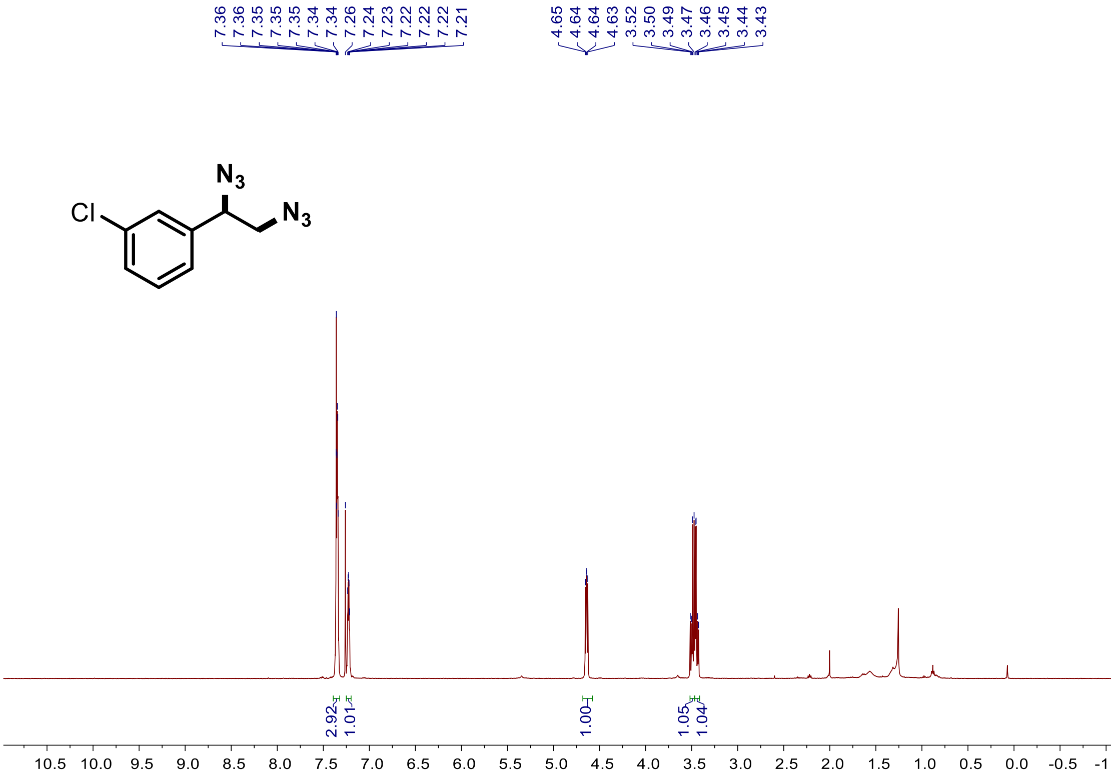

Supplementary Figure 68. ^{13}**C NMR** (101 MHz, $CDCl_3$, 298K) of **1-chloro-3-(1,2-diazidoethyl)benzene**

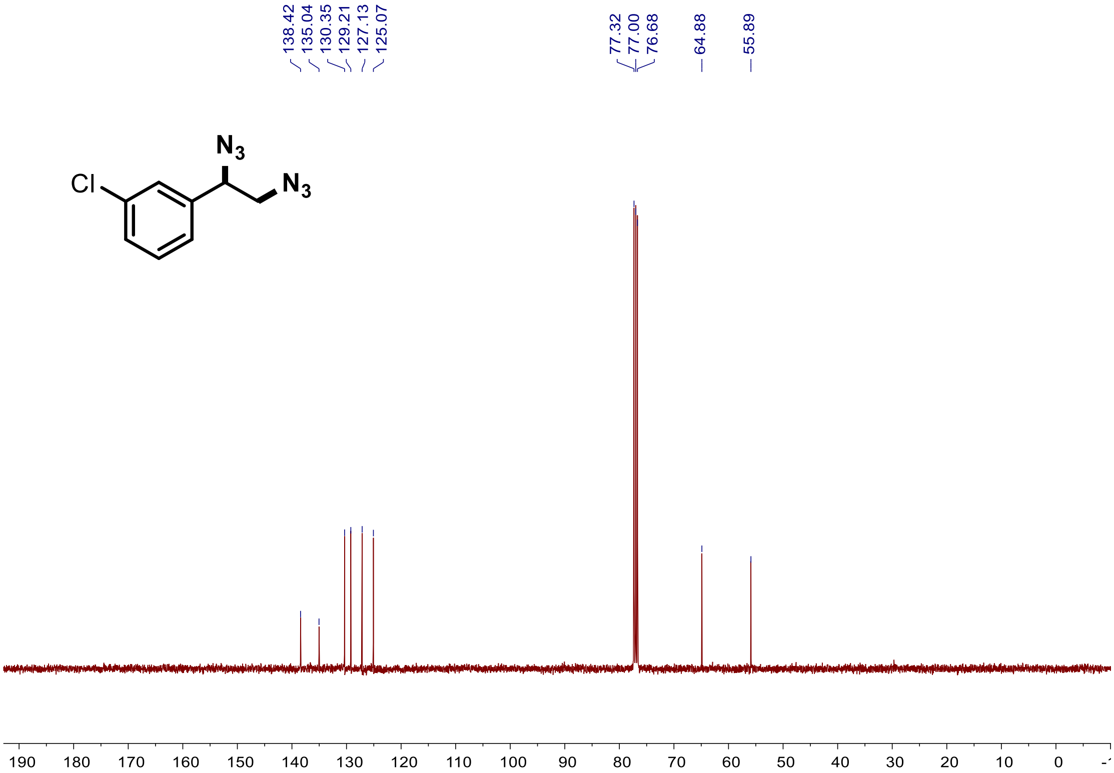

Supplementary Figure 69. ^{1}**H NMR** (500 MHz, $CDCl_3$, 298K) of **1-chloro-2-(1,2-diazidoethyl)benzene**

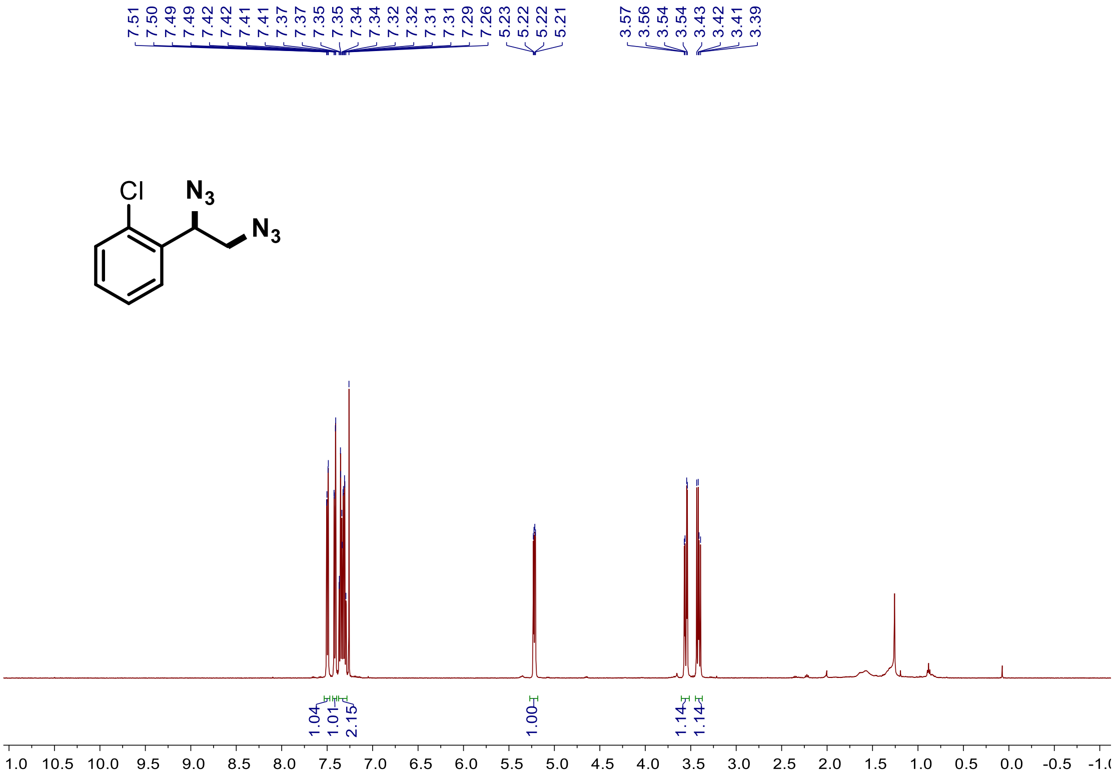

Supplementary Figure 70.^{13}**C NMR** (101 MHz, $CDCl_3$, 298K) of **1-chloro-2-(1,2-diazidoethyl)benzene**

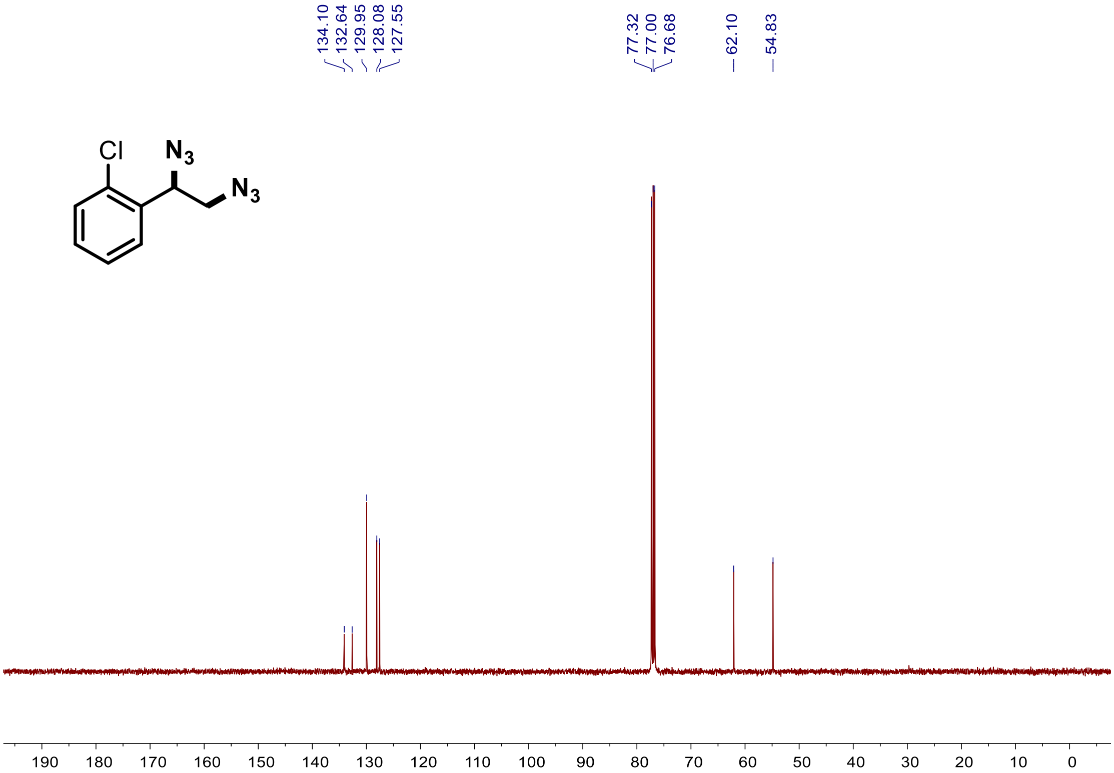

Supplementary Figure 71. ^{1}**H NMR** (500 MHz, $CDCl_3$, 298K) of **2-(1,2-diazidoethyl)naphthalene**

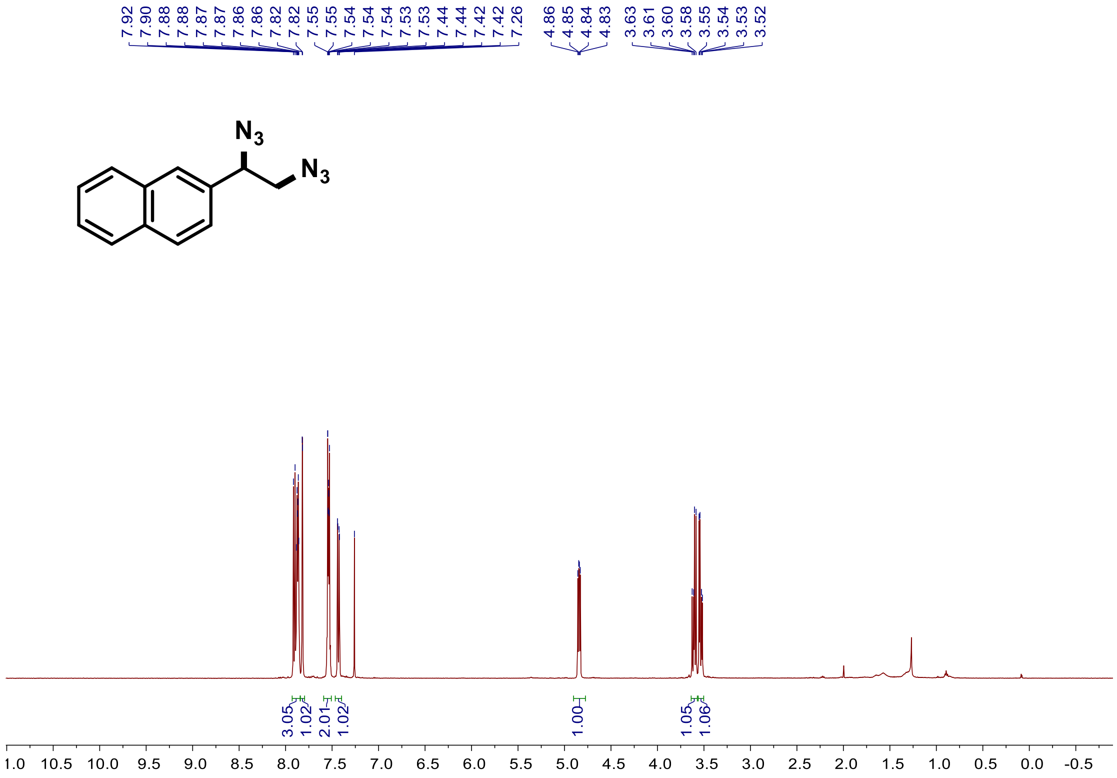

Supplementary Figure 72. ^{13}**C NMR** (101 MHz, $CDCl_3$, 298K) of **2-(1,2-diazidoethyl)naphthalene**

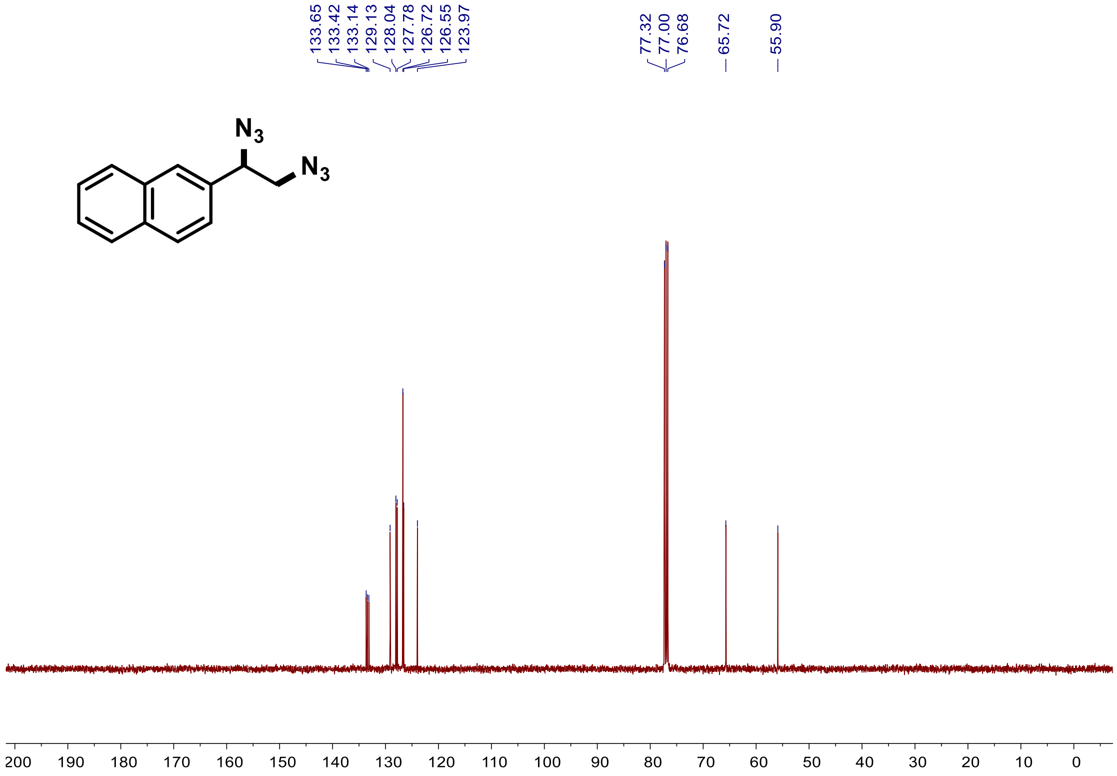

Supplementary Figure 73. ^{1}**H NMR** (500 MHz, $CDCl_3$, 298K) of **1,2-diazidopropyl)benzene**

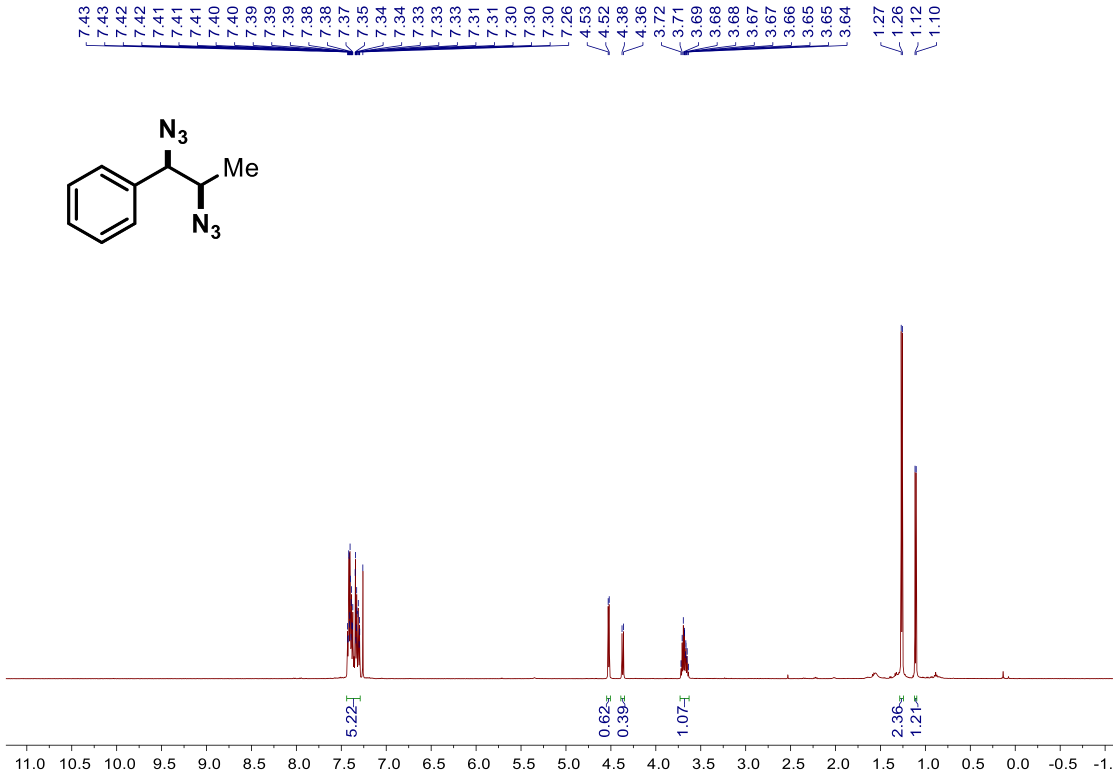

Supplementary Figure 74. ^{13}**C NMR** (101 MHz, $CDCl_3$, 298K) of **1,2-diazidopropyl)benzene**

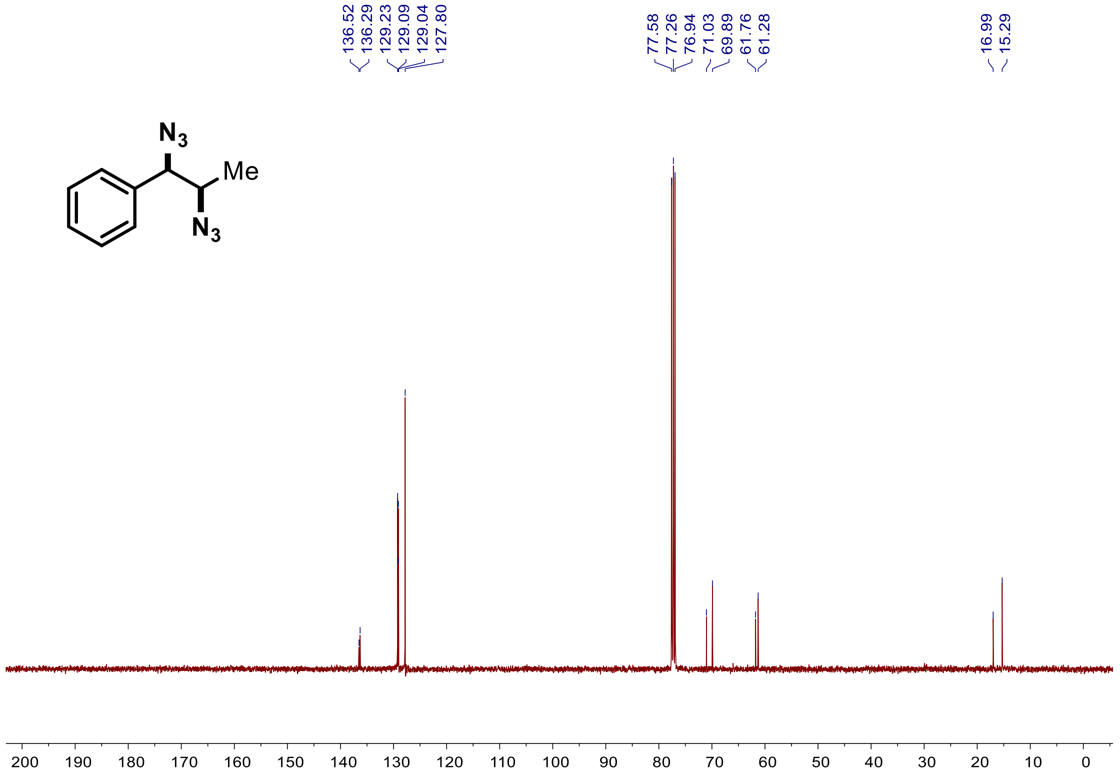

Supplementary Figure 75. ^{1}**H NMR** (500 MHz, $CDCl_3$, 298K) of **1,2-diazido-1,2-diphenylethane**

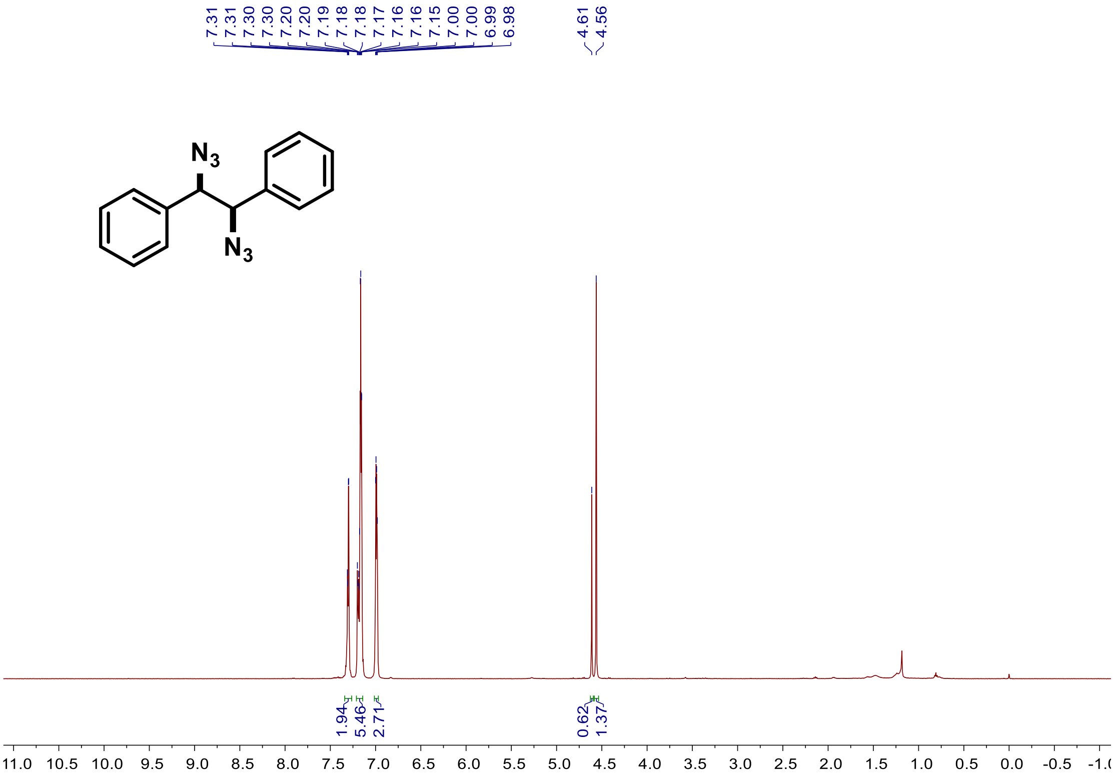

Supplementary Figure 76. ^{13}**C NMR** (101 MHz, $CDCl_3$, 298K) of **1,2-diazido-1,2-diphenylethane**

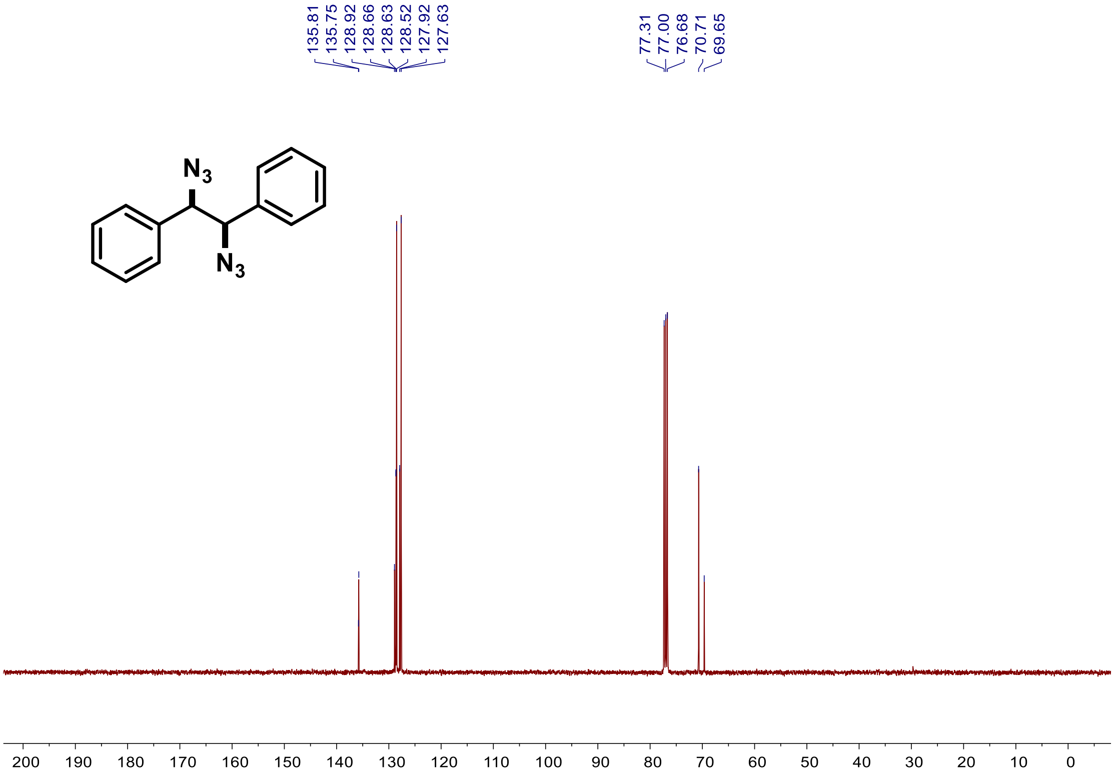

Supplementary Figure 77. ^{1}**H NMR** (500 MHz, $CDCl_3$, 298K) of **1,2-diazido-1,2,3,4-tetrahydronaphthalene**

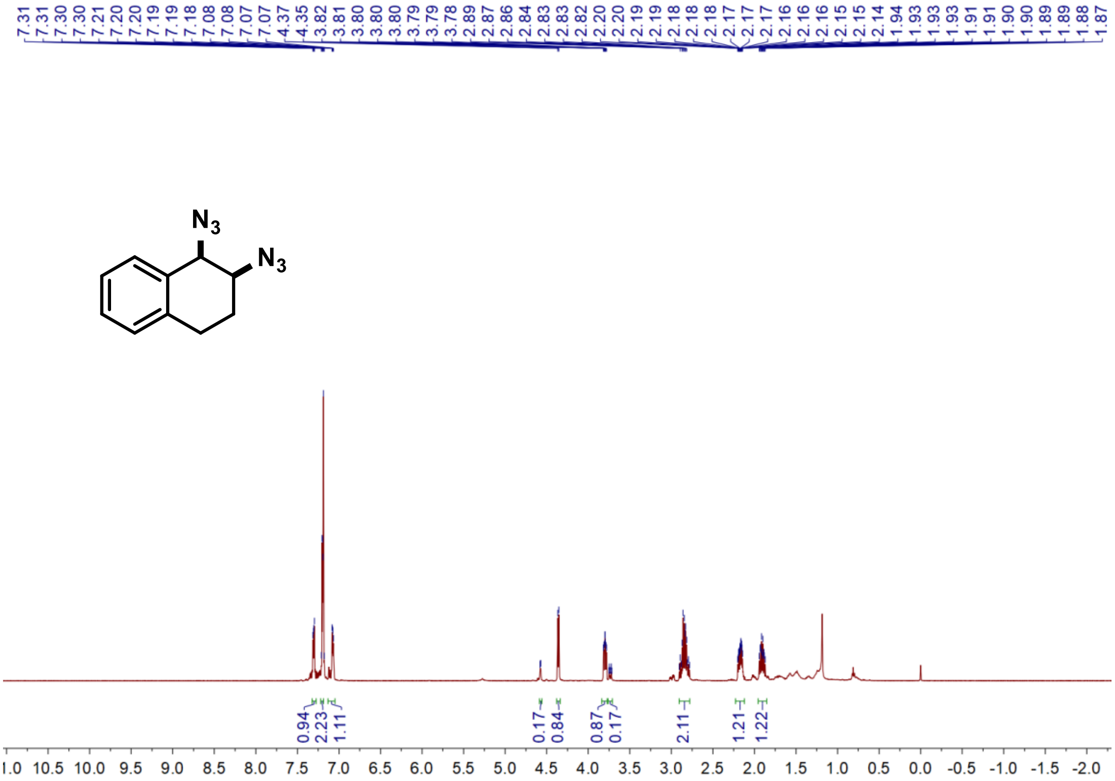

Supplementary Figure 78. ^{13}**C NMR** (101 MHz, $CDCl_3$, 298K) of **1,2-diazido-1,2,3,4-tetrahydronaphthalene**

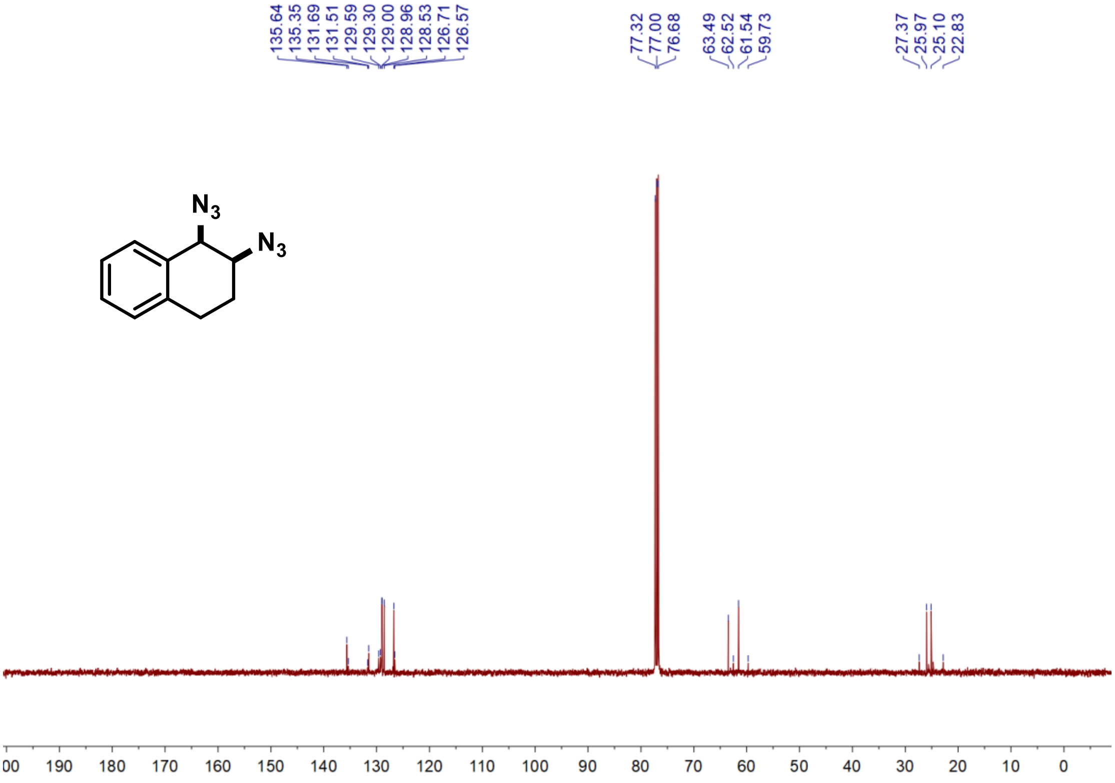

Supplementary Figure 79. **^{1}H NMR** (500 MHz, $CDCl_3$, 298K) of **(1R,2S,5R)-2-isopropyl-5-methylcyclohexyl 4,5-diazidopentanoate**

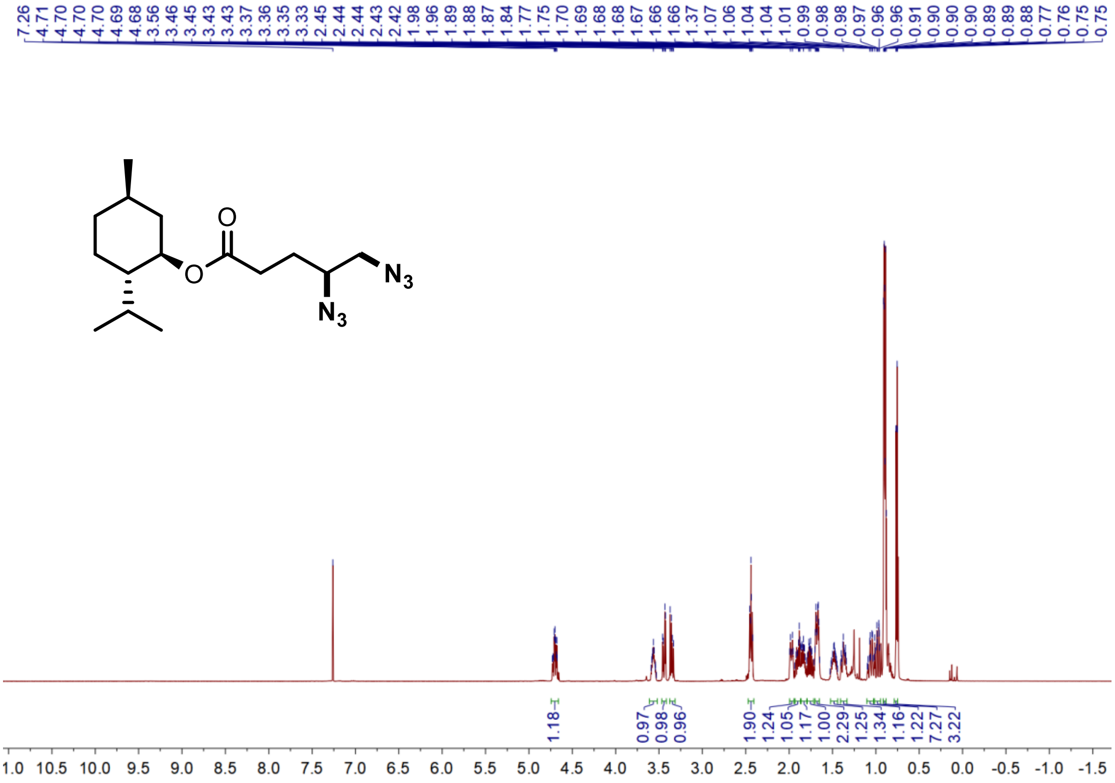

Supplementary Figure 80. **^{13}C NMR** (101 MHz, $CDCl_3$, 298K) of **(1R,2S,5R)-2-isopropyl-5-methylcyclohexyl 4,5-diazidopentanoate**

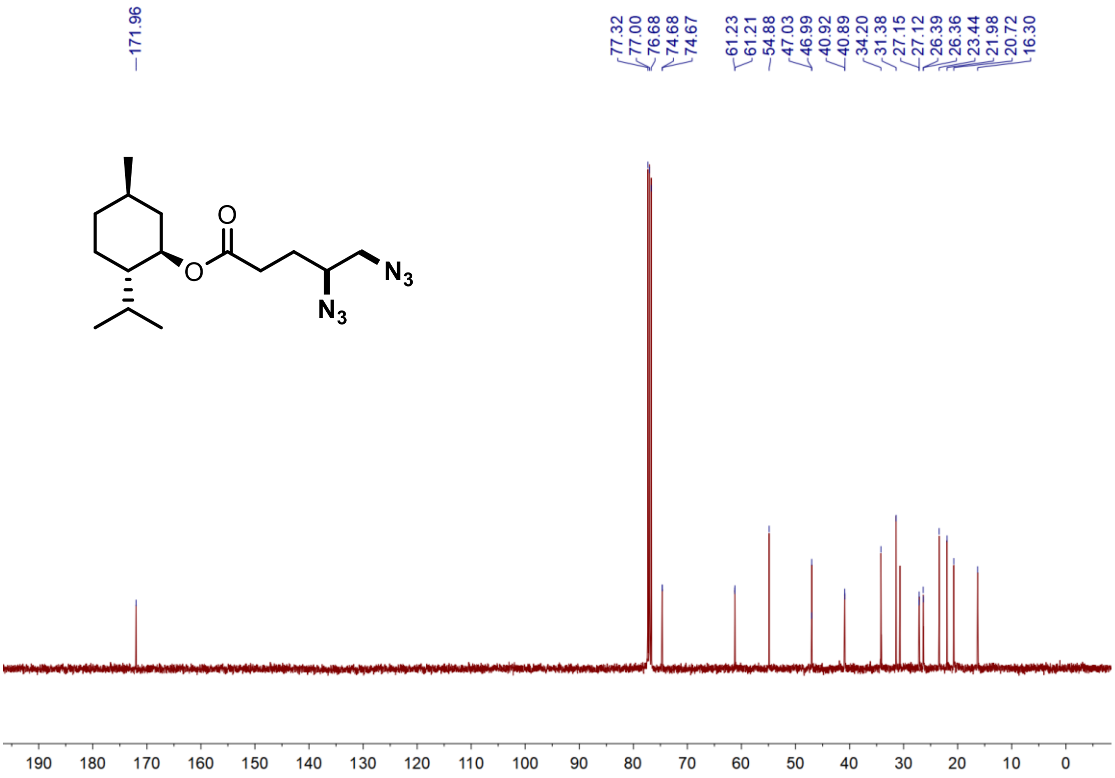

Supplementary Figure 81. ^{1}H NMR (500 MHz, $CDCl_3$, 298K) of **3,4-diazidobutyl 2-(4-isobutylphenyl)propanoate**

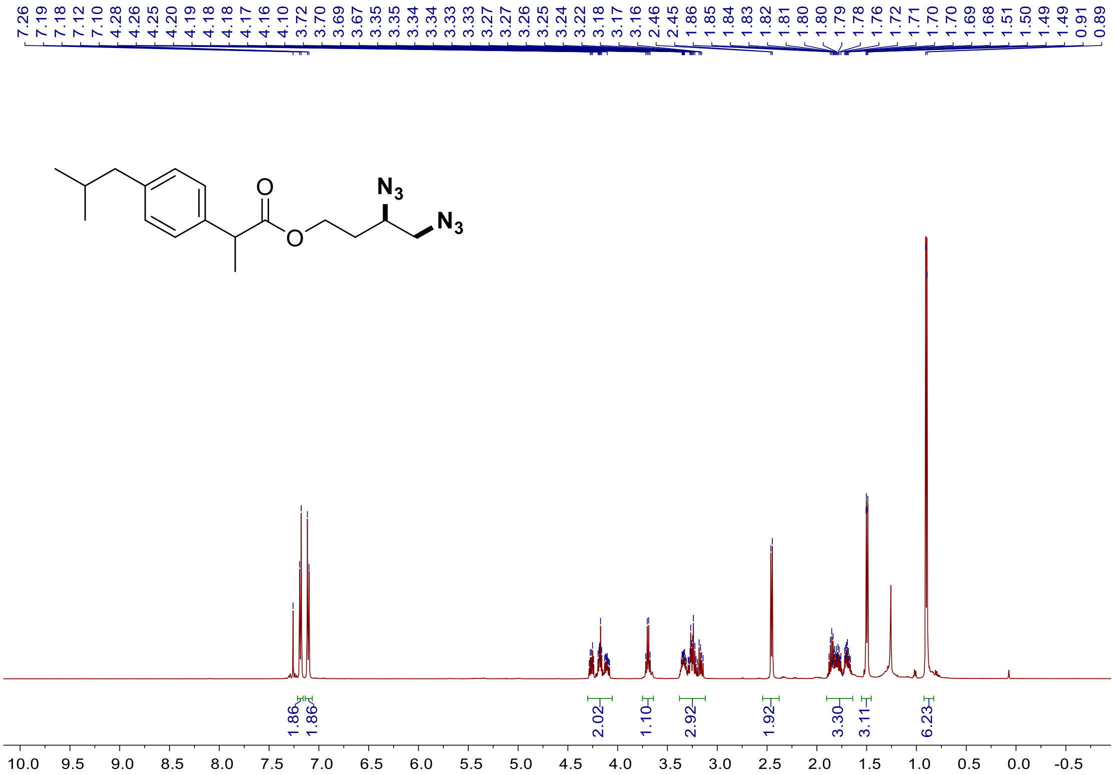

Supplementary Figure 82. ^{13}C NMR (101 MHz, $CDCl_3$, 298K) of **3,4-diazidobutyl 2-(4-isobutylphenyl)propanoate**

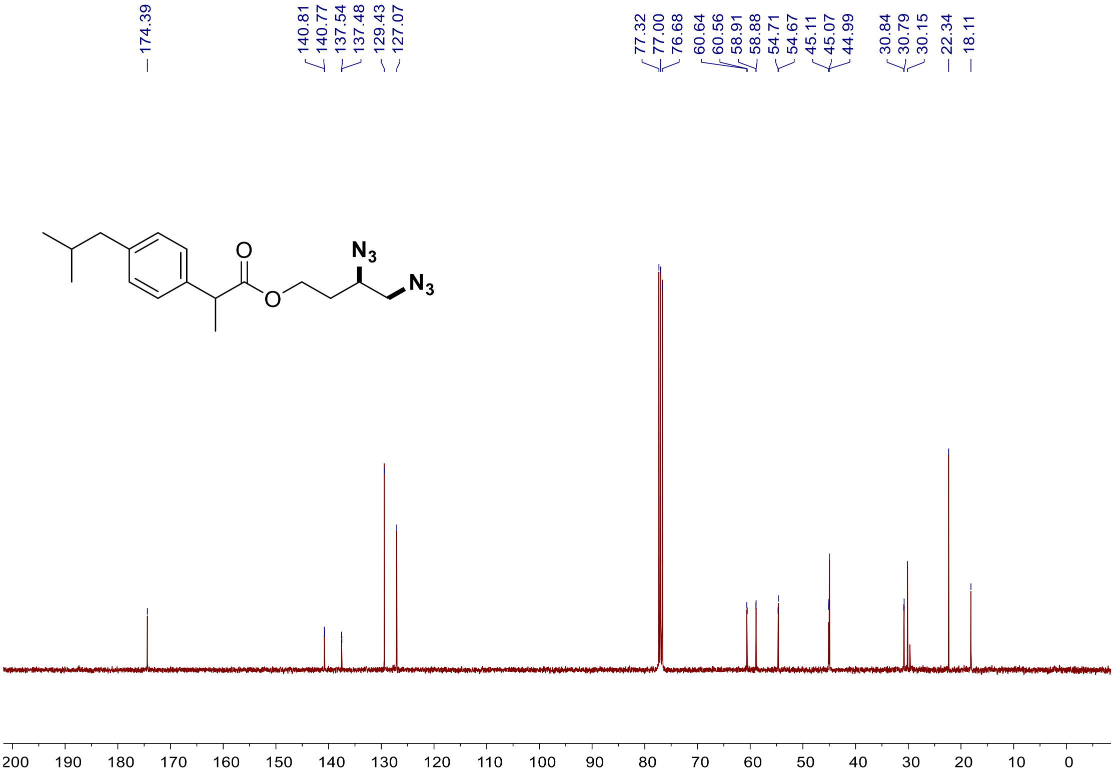

Supplementary Figure 83. ^{1}**H NMR** (500 MHz, $CDCl_3$, 298K) of **3,4-bis(azidomethyl)-1-tosylpyrrolidine**

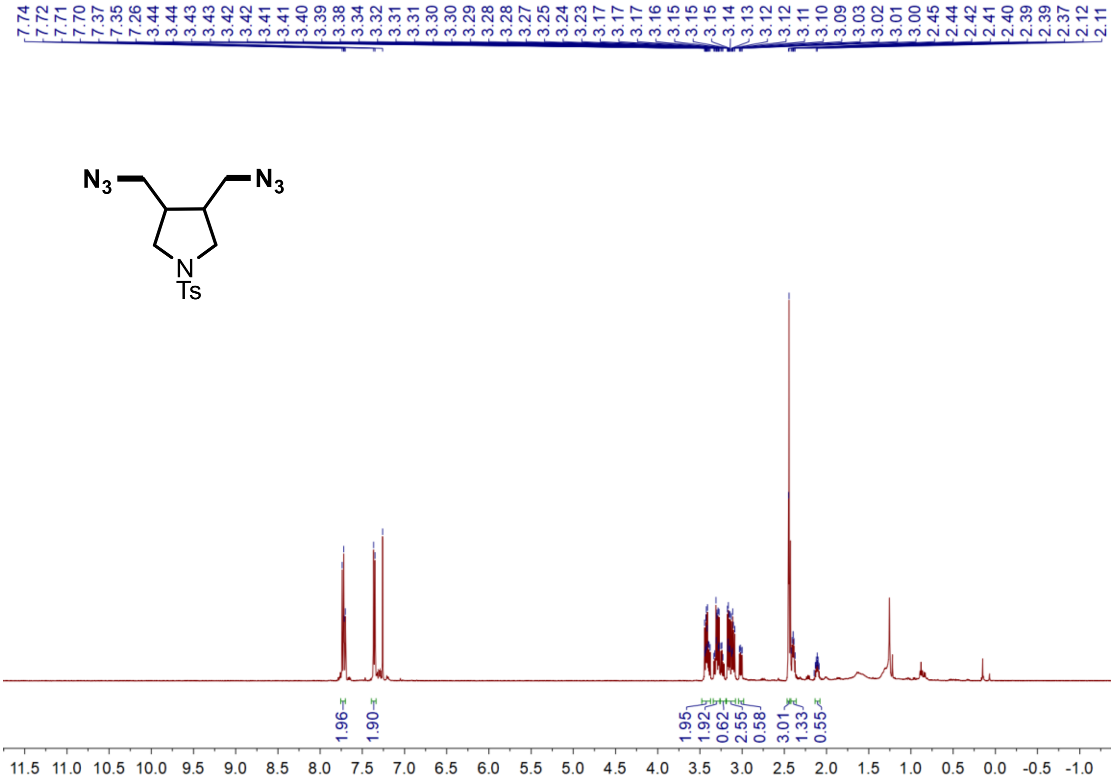

Supplementary Figure 84. ^{13}**C NMR** (101 MHz, $CDCl_3$, 298K) of **3,4-bis(azidomethyl)-1-tosylpyrrolidine**

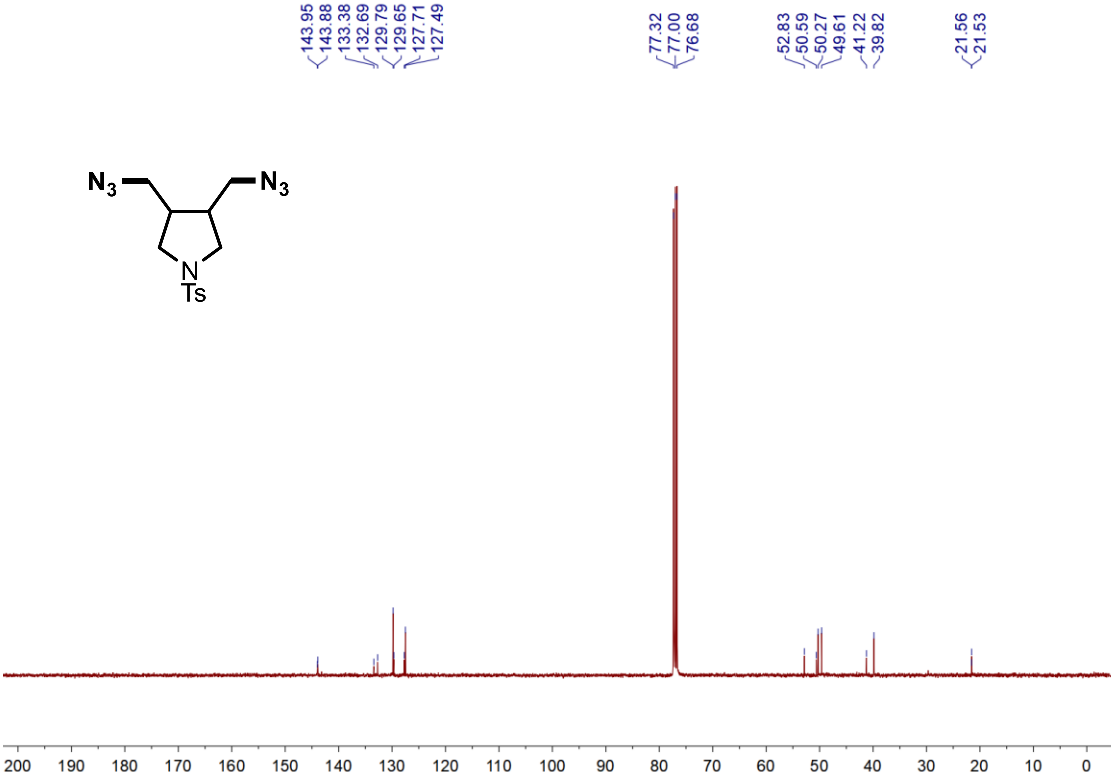

Supplement: Supplementary file 1 — Supplementary information [file 41467_2022_35344_MOESM1_ESM.pdf]
