# Supplementary material for: Iron-mediated ligand-to-metal charge transfer enables 1,2-diazidation of alkenes
Source: Nat Commun. 2022 Dec 23;13:7880. doi: 10.1038/s41467-022-35344-9 (PMC9789131; doi:10.1038/s41467-022-35344-9)
Supplement: Supplementary file 2 — Peer Review File [file 41467_2022_35344_MOESM2_ESM.pdf]

Peer Review File

Iron-mediated ligand-to-metal charge transfer enables 1,2-diazidation of alkenes

Open Access This file is licensed under a Creative Commons Attribution 4.0 International License, which permits use, sharing, adaptation, distribution and reproduction in any medium or format, as long as you give appropriate credit to the original author(s) and the source, provide a link to the Creative Commons license, and indicate if changes were made. In the cases where the authors are anonymous, such as is the case for the reports of anonymous peer reviewers, author attribution should be to 'Anonymous Referee' followed by a clear attribution to the source work. The images or other third party material in this file are included in the article's Creative Commons license, unless indicated otherwise in a credit line to the material. If material is not included in the article's Creative Commons license and your intended use is not permitted by statutory regulation or exceeds the permitted use, you will need to obtain permission directly from the copyright holder. To view a copy of this license, visit http://creativecommons.org/licenses/by/4.0/.

REVIEWER COMMENTS

Reviewer #1 (Remarks to the Author):

This manuscript is somewhat a little bit similar with that the editor asked this reviewer to review some times ago. Of course, it is just similar not the same.

In this manuscript, Muliang and coworkers have reported a light-induced, Fe-catalyzed 1,2-diazidation of alkenes. The reaction scope is good and the yields are moderate to good. The authors have tested many transiton metal catalysts and only Fe(NO3)3 can give the best results. It is so interestign that this can further accounts for the LMCT process. The 1,2-diazidation of alkenes, especially unactivated alkenes is important to the chemcial society.

This reviewer would like to strongly support its acceptance in NC after suitable minor revisions.

1. Cheap transition metal catalyzed 1,2-difucntionalization of unactivated alkenes are suggested to cite as Nat. Synth. 2022, 1, 475-486.

2. The authors should be more careful for the exclusion of carbon cation intermedaite. Maybe more experiments are welcome or compromised description for this.

3. Hoe about the low power lamp, such as 5 W?

4. The wavelength of used lamp should be provided in text and Supplemenatry Information.

Anway, the authors have made a good contribution for the selective 1,2-diazidation of unactivated alkenes. The products are synthetcialy useful and versatile in organic synthesis. It will gain much attention for the reaction mechanism about LMCT process. MInor revision are nedded before publication.

Reviewer #2 (Remarks to the Author):

The authors report an operationally simple and environmentally benign diazidation of an alkene using an iron complex to deliver an overall alkene diamination. Noteworthy in the present reaction is the implication of an azido radical, presumably generated from the iron via an LMCT process.

The authors mention that the radical rebound mechanism in the reported copper-catalyzed alkyl trifluoromethylation (ref 39-41) inspired the azido radical transfer to an alkyl radical in the present

reaction. This radical rebound of an iron complex is also well-known, especially in oxidation reactions of alkyl radicals. An azido radical transfer from an iron complex to an alkyl radical has been previously described (ACIE. 2021, 60, 12455 – not cited).

The strength of this manuscript is that we always need other methods to achieve desirable transformations and the existence of the electrochemical catalytic diazidation reported by Lin and Xia (refs 25 and 26) should not heavily detract from this work. There is, however, a different precedent that is not cited and that is more problematic: Honglie Bao's ACIE 2021, 60, 12455-12460. This latter manuscript reports the Fe catalyzed diazidation of styrene type olefins and mirrors much of the reactivity reported in this paper with the caveat that it is catalytic in Fe and does not need light.

On the whole, diazidation is useful and we always need more methods, but it would have been nice to see this rendered catalytic given that the state-of-the-art technology is catalytic (electrochemical diazidation) and the strongest precedent (Bao's work) is also catalytic, if one is to make the argument for acceptance to a journal like Nature Communications.

Minor issues:

1. The authors tried to use white LEDs instead of 40 W blue LEDs in the optimization. The white LEDs was phrased as "38 White LEDs" in the text (line 109 and Table 1, entry 7). Presumably, "W" (watt) was omitted after "38". If "38 White LEDs" is the name of a particular product, it should be changed to just "white LEDs".

2. The authors state the electronic and steric effects of substituents on the aromatic ring in 1,1-disubstituted alkenes were investigated in line 128 (Figure 2, 2a-f). The alkenes were actually 4-aryl-2-methyl-1-butenes, of which a substituent on the arene ring predictably could not influence the reactivity because the arene ring is not close to the double bond.

3. The picture depicting the scale-up in Figure 2 is completely unintelligible in my version.

4. The diastereomeric ratio should be shown in the reaction with the alkene having l-menthol structure (2j').

5. The iron complex in the present reaction should be reduced after the reaction and no longer catalytic. The phrase "iron photocatalysis" in line 185 is misleading.

6. Norcamphene is too simple to claim late-stage modification.

7. The same paper is cited on two different numbers (7, 20).

Response to the reviewers

We deeply appreciate the time you and the reviewers spent to evaluate this work, and the valuable suggestion from you and the reviewers, which significantly helped us to further improve the quality of our work. We managed to address all the comments made by the reviewers. The manuscript has been revised accordingly.

Our detailed responses are listed below:

<u>Response to Reviewer # 1</u>

Comment 1*: In this manuscript, Muliang and coworkers have reported a light-induced, Fe-catalyzed 1,2-diazidation of alkenes. The reaction scope is good and the yields are moderate to good. The authors have tested many transition metal catalysts and only $Fe(NO_3)_3$ can give the best results. It is so interesting that this can further accounts for the LMCT process. The 1,2-diazidation of alkenes, especially unactivated alkenes is important to the chemical society. This reviewer would like to strongly support its acceptance in NC after suitable minor revisions.*

Response to Comment 1: We appreciate Reviewer #1 for all the supportive comments.

Comment 2**:** *Cheap transition metal catalyzed 1,2-difucntionalization of unactivated alkenes are suggested to cite as Nat. Synth. 2022, 1, 475-486.*

Response to Comment 2: We thank Reviewer #1 for this suggestion. From the green and sustainable chemistry points of view, there is a longstanding interest in replacing the precious metals by more and cheaper earth-abundant elements. We have included the highly related literature in the updated references as ref 30 in the revised manuscript.

Comment 3: *The authors should be more careful for the exclusion of carbon cation intermedaite. Maybe more experiments are welcome or compromised description for this.*

Response to Comment 3: We thank Reviewer #1 for this comment and important suggestions. The intermolecular trapping experiments were performed with a large excess of nucleophiles such as H_2O and EtOH added into reaction system, however, the compounds trapped by nucleophiles could not be detected. A radical-polar crossover mechanism should be excluded. These trapping experiments have been added into revised SI.

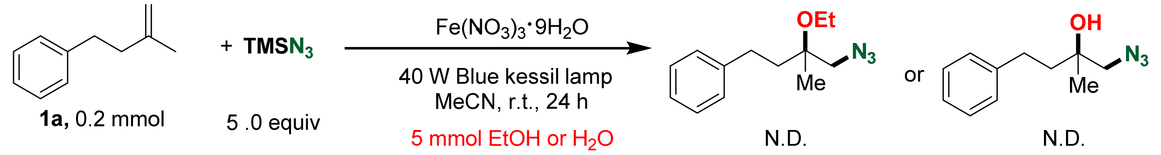

Comment 4*:* *How about the low power lamp, such as 5 W?*

Response to Comment 4: We thank Reviewer #1 for this comment. The use of 5 W blue light decreased the efficiency of the reaction, leading to inferior yield (56% yield).

Comment 5*: The wavelength of used lamp should be provided in text and Supplemenatry Information.*

Response to Comment 5: We thank Reviewer #1 for this comment. The wavelength of used lamp had been provided.

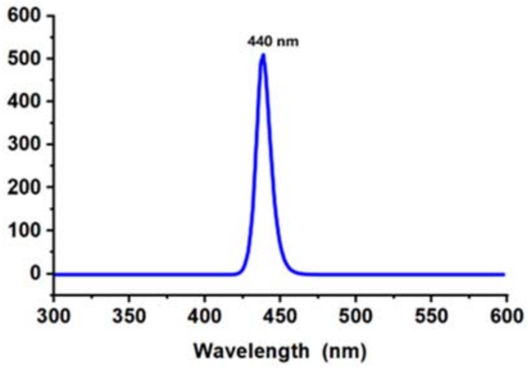

Figure S1. Emission spectra of the blue LED light source.

<u>Response to Reviewer # 2</u>

Comment 1*: The authors report an operationally simple and environmentally benign diazidation of an alkene using an iron complex to deliver an overall alkene diamination. Noteworthy in the present reaction is the implication of an azido radical, presumably generated from the iron via an LMCT process.*

Response to Comment 1: We thank Reviewer #2 for all the supportive comments.

Comment 2**:** *The authors mention that the radical rebound mechanism in the reported copper-catalyzed alkyl trifluoromethylation (ref 39-41) inspired the azido radical transfer to an alkyl radical in the present reaction. This radical rebound of an iron complex is also well-known, especially in oxidation reactions of alkyl radicals. An azido radical transfer from an iron complex to an alkyl radical has been previously described (ACIE. 2021, 60, 12455 – not cited). The strength of this manuscript is that we always need other methods to achieve desirable transformations and the existence of the electrochemical catalytic diazidation reported by Lin and Xia (refs 25 and 26) should not heavily detract from this work. There is, however, a different precedent that is not cited and that is more problematic: Honglie Bao's ACIE 2021, 60, 12455-12460. This latter manuscript reports the Fe catalyzed diazidation of styrene type olefins and mirrors much of the reactivity reported in this paper with the caveat that it is catalytic in Fe and does not need light. On the whole, diazidation is useful and we always need more methods, but it would have been nice to see this rendered catalytic given that the state-of-the-art technology is catalytic (electrochemical diazidation) and the strongest precedent (Bao's work) is also catalytic, if one is to make the argument for acceptance to a journal like Nature Communications.*

Response to Comment 2: We thank Reviewer #2 for these insightful and constructive comments.
In this work (*Angew. Chem. Int. Ed.* **60**, 12455–12460, (2021)), Bao's group developed iron-catalyzed radical azidation and diazidation of styrenes using external oxidants to regenerate iron catalyst or generate

a radical intermediate. Substrate scopes are limited to styrenes and external oxidants are required. This related literature in the updated references has been added as ref 21 in the revised manuscript.
The integration of electrochemistry with transition metal catalysis has significantly expanded the scope of organic electrosynthes. Lin as well as Xu et al. reported electrochemical catalytic diazidation (*Science* **357**, 575-579 (2017); *J. Am. Chem. Soc.* **144**, 11980-11985 (2022)), where precise control of the potential applied on the electrodes and the cell voltage was required to access an azide radical through continuous anodic oxidation.
With the growing appreciation of the significance and utility of these diazidation reactions, the need for efficient methods continues to inspire the development of new strategies, pathway and concepts. Compared to these developed oxidative reactions, ligand-to-metal charge transfer (LMCT) manifold has provided an attractive pathway to access radical intermediates without external oxidants, however, such a activation mode has comparatively been underexploited in the realm of synthetic organic chemistry.
Herein we reported that Fe-N_3-based complex generated from readily available Fe salts and azido sources could be easily photoexcited by visible-light irradiation and subsequently could undergo Fe(III)−N_3 homolysis to release an azide radical through LMCT process. Such a pathway to generate an azide radical is very desriable by avoiding to use strong oxidants. The generated azide radical will readily add to carbon–carbon bond to provide a carbon radical intermediate, followed by trapping of Fe(III)−N_3 complex, providing the desired diazides with a broad range of substrate scope. In addition, this method presents a very simple operation, just using a simple and cheap iron salt and azide source compared to screening of complex parameters in electrochemical catalysis or transition metal catalysis (expensive ligand or harsh reactions are required). The catalytic system is always appealing and we're investigating the possibility of this catalytic protocol in our lab.

***Comment 3*:** *Minor issues:*

1. *The authors tried to use white LEDs instead of 40 W blue LEDs in the optimization. The white LEDs was phrased as "38 White LEDs" in the text (line 109 and Table 1, entry 7). Presumably, "W" (watt) was omitted after "38". If "38 White LEDs" is the name of a particular product, it should be changed to just "white LEDs".*

   Corrected. Thank you for spotting this error. It has been changed into 38 W White LEDs.

2. *The authors state the electronic and steric effects of substituents on the aromatic ring in 1,1-disubstituted alkenes were investigated in line 128 (Figure 2, 2a-f). The alkenes were actually 4-aryl-2-methyl-1-butenes, of which a substituent on the arene ring predictably could not influence the reactivity because the arene ring is not close to the double bond.*

   We thank Reviewer #2 for this comment and this sentence has been changed to "1,1-disubstituted type of alkenes were investigated under the optimized reaction conditions".

3. *The picture depicting the scale-up in Figure 2 is completely unintelligible in my version.*

   We thank Reviewer #2 for this comment. We have put a new picture to show the scale-up reaction as shown below.

Scale up

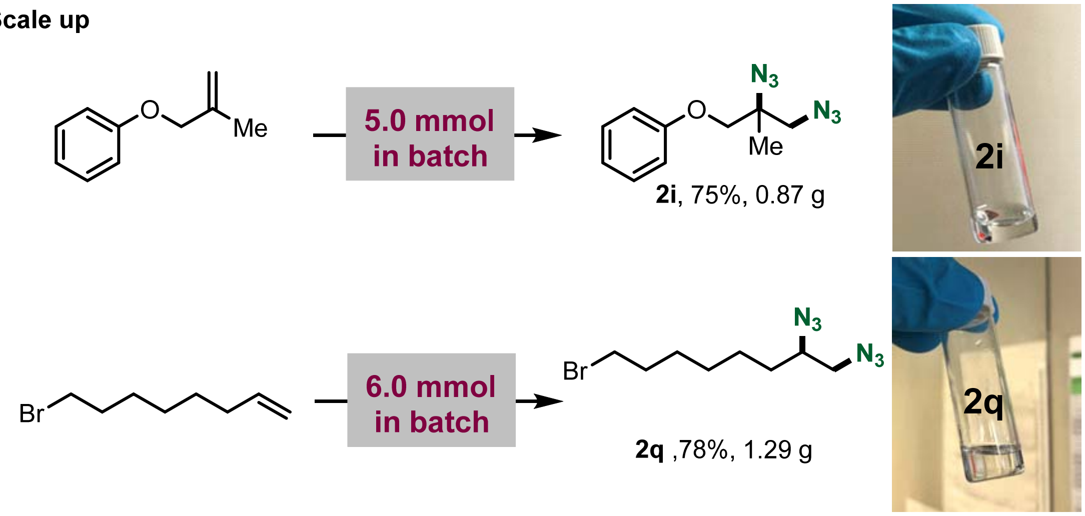

4. *The diastereomeric ratio should be shown in the reaction with the alkene having l-menthol structure (2j').*

   We thank Reviewer #2 for this comment and diastereomeric ratio (1/1) of **2j'** was determined by ^{1}H NMR analysis.

5. *The iron complex in the present reaction should be reduced after the reaction and no longer catalytic. The phrase "iron photocatalysis" in line 185 is misleading.*

   We thank Reviewer #2 for this comment and it has been changed to "iron-mediated LMCT mode"

6. *Norcamphene is too simple to claim late-stage modification.*

   We thank Reviewer #2 for this comment. Complex alkene derived from ibuprofen was suitable to deliver vicinal diazide product **2k'** in 64% yield and this example was added to late-stage modification part.

7. *The same paper is cited on two different numbers (7, 20).*

   Thank you for spotting this error and it has been corrected.

REVIEWERS' COMMENTS

Reviewer #1 (Remarks to the Author):

Muliang made a good revision for this work. This reviewer would like to recommend its publciation.

REVIEWERS' COMMENTS

Reviewer #1 (Remarks to the Author):

Reviewer #1 (Remarks to the Author):
Muliang made a good revision for this work. This reviewer would like to recommend its publciation.

Thank you very much.
